# Accounting for temporal variation and correlation in environmental DNA sampling can improve ecological inferences

**DOI:** 10.1002/eap.70290

**Published:** 2026-08-04

**Authors:** Ben C. Augustine, Patrick R. Hutchins, Margaret E. Hunter, Christopher M. Merkes, David S. Pilliod, Stephen F. Spear, Caitlin E. Beaver, Scott D. George, Devin N. Jones‐Slobodian, Adam J. Sepulveda

**Affiliations:** ^1^ U.S. Geological Survey Northern Rocky Mountain Science Center Bozeman Montana USA; ^2^ U.S. Geological Survey Wetland and Aquatic Research Center Gainesville Florida USA; ^3^ U.S. Geological Survey Upper Midwest Environmental Sciences Center La Crosse Wisconsin USA; ^4^ U.S. Geological Survey Forest and Rangeland Ecosystem Science Center Boise Idaho USA; ^5^ U.S. Geological Survey New York Water Science Center Troy New York USA

**Keywords:** hierarchical model, quantitative PCR, survey design, temporal correlation, time series

## Abstract

Environmental DNA (eDNA) concentration varies through space and time, and measurements collected close together are often correlated. Ignoring this dependence can inflate the rate of incorrect ecological inferences (Type I error rate). Although spatial correlation in eDNA has received considerable attention, temporal correlation has been less well studied. Statistical models and study designs that account for temporal correlation are increasingly important to understand time‐dependent effects in complex systems. We developed a hierarchical model that separates temporal ecological variation from variability stemming from sampling and laboratory processes and applied it to four single‐site eDNA time series collected over 17–24 days, three of which provided sufficient information for parameter estimation. We then used the empirically estimated parameter magnitudes in a simulation study to evaluate alternative temporal sampling designs that considered (1) how a fixed number of samples are allocated across different numbers of sampling times with different levels of temporal replication and (2) equally spaced versus cluster‐spaced sampling (short bursts separated by longer gaps). Across the three time series sufficient for analysis, we observed substantial sampling variability, temporal variability, and temporal correlation, although correlation was estimated imprecisely (large coefficient of variation). Simulations showed that when sampling intervals were shorter than the effective temporal correlation range, models that ignored temporal dependence produced inflated Type I error rates and frequently detected spurious temporal trends. Accounting for temporal correlation substantially reduced this inflated Type I error rate. Optimal sampling strategies depended on study objectives. Clustered sampling most effectively estimated temporal correlation. When temporal dependence was negligible, evenly spaced sampling maximized power to detect trends. Estimating sampling variability required concentrating effort into fewer sampling times with more replicates per time, whereas estimating temporal variance was most precise with intermediate levels of replication. Together, these results indicate that temporal dependence can strongly affect inference from quantitative eDNA time series when sampling intervals approach the correlation timescale. Designs that ignore this dependence risk inferring ecological change or difference where none exists. Our framework provides practical guidance for allocating sampling effort in temporally intensive eDNA monitoring and for interpreting trends from short time series.

## INTRODUCTION

Environmental DNA (eDNA) sampling offers unique advantages for aquatic species monitoring, as it can detect genetic traces of individuals captured in water samples. The eDNA concentration of samples can be measured to infer the presence of rare taxa and can serve as indicators of absolute abundance and population trends, all without visual observations (e.g., Jerde et al., 2011; Rourke et al., 2022; Takahara et al., 2012; Yates et al., 2019). However, eDNA sampling can result in false negative detections, and sampling variability (among water samples) in eDNA concentration can be large (Shelton et al., 2022; Yates et al., 2023). Therefore, intensive sampling may be required to achieve an acceptable cumulative detection probability or accurately estimate site eDNA concentrations in any one location (Yates et al., 2023). Given the expense of eDNA sampling and processing, efficient study designs and sampling strategies are important for maximizing the information per sample and limiting project costs.

Spatiotemporal patterns of eDNA concentration in the environment are of wide ecological interest and an area where sampling design is particularly important, especially in aquatic ecosystems where the environment is continuous in multiple dimensions: horizontal, vertical (i.e., depth), and temporal (e.g., flow, diel) (Mathieu et al., 2020). eDNA concentration in the environment may vary across space due to spatial patterns in ecological and hydrological factors that influence species distributions and eDNA production, fate, and transport (Carraro et al., 2018; Chen & Ficetola, 2019). Similarly, eDNA concentration at a particular location may vary over time, especially in relation to eDNA production from organisms, eDNA degradation (e.g., changes in UV, temperature), and eDNA transport (e.g., hydrologic effects; George et al., 2024; Jensen et al., 2022). In the presence of spatiotemporal variation, we often expect spatiotemporal correlation, meaning eDNA concentration measurements closer together in space and time to be more similar than those farther apart (Chen & Ficetola, 2019; Searcy et al., 2022). As a result, when sampling under these conditions, ecological inference may be unreliable if models fail to adequately account for spatiotemporal autocorrelation. In such cases, the residuals are no longer independent (Gimenez, 2024), which can lead to underestimated parameter uncertainty and an inflated Type I error rate (in a statistical sense, not to be confused with false positive eDNA detections; Millard et al., 1985). Spatial variability and autocorrelation are relatively well studied and have been considered in eDNA sampling designs (e.g., Carraro et al., 2018; Chen & Ficetola, 2019), but temporal variability and correlation have received relatively less attention (Beentjes et al., 2019; Jensen et al., 2022; Mathieu et al., 2020).

Temporal variation in eDNA concentration is often of intrinsic ecological interest, as it may indicate changes in the distribution, abundance, life history, and even behavior of the target species or the environmental conditions associated with these changes (Buxton et al., 2017; Erickson et al., 2016). Changes in environmental conditions can also influence rates at which eDNA degrades or is transported, which can confound temporal relationships between eDNA concentrations and the organisms that produce them. Repeated sampling at the same site over time can provide data that allow temporal variation in eDNA concentration to be related to ecological, physical, and hydrological factors (George et al., 2024) to better understand their independent effects. The advent of eDNA autosamplers that can more easily collect repeated samples over time and can be co‐located with other measurement instruments, like stream gages, could facilitate such sampling designs (e.g., George et al., 2024; Sepulveda, Birch, et al., 2020), but consideration of temporal correlation may be critical for reliable ecological inference. Finally, eDNA temporal variation is also relevant to the investigation of spatial variability. A site's eDNA concentration at a single point in time or multiple times over a short temporal window may not be representative of the site's eDNA concentration over longer periods of time. Moreover, if sites are sampled at different times, unmodeled temporal variability can be confounded with spatial variability (Beentjes et al., 2019).

In addition to spatiotemporal variation, eDNA sampling is characterized by potentially large sampling variability and measurement error (Augustine et al., 2024; Morrison et al., 2023; Shelton et al., 2022; Yates et al., 2023). These factors are inconsistently considered, despite the potential for this variation to be erroneously combined with ecological sources of variation of interest (Augustine et al., 2024; Mathieu et al., 2020). A particularly challenging source of measurement error stems from factors that reduce polymerase chain reaction (PCR) efficiency, such as inhibitors or reduced stochastic binding of primers/probes and polymerase to the target sequence, particularly at lower eDNA concentrations, leading to systematically underestimated eDNA concentrations (Sepulveda, Hutchins, et al., 2020; Zhao et al., 2016). Hierarchical models provide a means to better isolate ecologically relevant sources of spatiotemporal variation from the sampling, replication, and measurement processes (Augustine et al., 2024; Mathieu et al., 2020). If properly specified, hierarchical models also provide a means of estimating the concentrations of non‐detected replicates, removing the bias in quantitative measurements, and modeling undermeasurement (Augustine et al., 2024).

Our goal is to quantify the magnitude of temporal correlation in quantitative eDNA time series data and illustrate its consequences for ecological inference and sampling design. Because sampling variability and measurement processes can obscure temporal dynamics, we first describe a temporal hierarchical model (Berliner, 1996) that separates ecological temporal variation from sampling and laboratory observation processes. During model evaluation, we identified evidence of systematic undermeasurement in some datasets, likely due to PCR inhibition, and incorporated this observation process to avoid confounding measurement bias with ecological variation. We then apply this model to four empirical eDNA time series to estimate the magnitude of temporal variability and correlation across diverse aquatic systems. Using these empirically derived parameter magnitudes, we illustrate how ignoring temporal correlation affects inference, particularly false detection of temporal trends. Finally, we evaluate alternative temporal sampling designs to show how sampling effort can be allocated across time to support reliable ecological inference. Together, these results provide initial guidance for the design of eDNA monitoring programs, particularly when pilot data are limited and autosamplers are used to collect time series data.

## METHODS

### Field and lab methods

#### Approach

We conducted eDNA sampling across a diverse set of freshwater habitats, including small creeks in Idaho and Montana, an impoundment of a large river in Wisconsin, and an experimental pond in Florida (site‐level details are below). The target taxa at these sites ranged from relatively stationary mollusks to more mobile fishes, potentially resulting in distinct spatiotemporal patterns of eDNA concentrations.

Water samples were collected 4 days per week for 3 weeks, except at the Florida site where a hurricane disrupted one sampling day, which was simply repeated during the next sampling period. The frequency of daily sampling was randomly assigned, without duplication, each week to one of four levels: one survey event per day, two survey events per day 6 h apart, three survey events per day 4 h apart, and four survey events per day 3 h apart. Three samples (i.e., temporal replicates) were collected for the one‐ and two‐survey‐event levels; otherwise, one sample was collected. Sampling began at the same time each day and sampling weeks were consecutive, unless otherwise noted. All samples were collected from the shore or a dock to minimize contamination potential.

When possible, similar field and lab methods were used across sites, which included filter type, filtered volume, filter preservation, and elution volume. Field and lab negative controls were associated with all sampling events. These data sets are summarized in Table 1. Below, we briefly summarize the field and lab sampling methods for each site with further details in Appendix S1.

**TABLE 1:** Summaries of the empirical data sets from four sites, including the number of unique sampling times (unique times), the total number of samples (samples), the number of technical replicates per sample (*K*), the number of temporal replicates (replicate samples), the time difference between the first to last sampling time (total time), and the empirical detection probabilities at the replicate ($p^{\mathrm{rep}}$) and sample ($p^{\text{samp}}$) levels (one or more detected replicates).

Site	Unique times	Samples	*K*	Replicate samples	Total time (h)	$p^{\mathrm{rep}}$	$p^{\text{samp}}$
MT	30	48	5	18	537	0.58	0.75
WI	30	90	4	60	424	0.41	0.74
FL	22	72	5	50	589	0.82	0.94
ID	36	108	3	72	408	0.11	0.69

Abbreviations: FL, Florida; ID, Idaho; MT, Montana; WI, Wisconsin.

#### Site #1: Montana (MT)

We sampled Bozeman Creek from 19 September to 5 October 2022 near downtown Bozeman, MT. At this location, the creek has an elevation of 1470 m, width of 3 m, drainage area of 145 km^2^, and average water temperature and discharge across the 3‐week period was 11°C and 0.33 m^3^/s, respectively (Montana DNRC, 2022; U.S. Geological Survey, 2019). The eDNA target was the New Zealand mudsnail (*Potamopyrgus antipodarum*), an aquatic invasive species known to occur in this creek at very low densities. One‐liter water samples were collected, transported to the U.S. Geological Survey (USGS) Northern Rocky Mountain Science Center Laboratory (Bozeman, MT) on wet ice, and immediately filtered through 45 mm diameter, 1.2 μm polyethersulfone (PES) filters using sterile, single‐use polypropylene filter cups and a peristaltic pump. New Zealand mudsnail eDNA analysis used the assay described by Goldberg et al. (2013). Five quantitative PCR (qPCR) replicates were run for each sample on a BioRad CFX 96‐Touch or BioRad Opus thermal cycler (Hercules, CA) and all replicates were multiplexed with an internal positive control (IPC).

#### Site #2: Wisconsin (WI)

We sampled Lake Onalaska (an impoundment of the Mississippi and Black Rivers) near La Crosse, WI, within the timeframe of 25 July 2022–11 August 2022. At this location, the lake has an elevation of 194 m, width of 609 m, and an approximate drainage area of 163,169 km^2^ (U.S. Geological Survey, 2019). The eDNA target was northern pike (*Esox lucius*), a native fish species. At each time point, three 1 L water samples were collected simultaneously using a Smith‐Root eDNA sampler (Vancouver, WA) and filtered through 45 mm diameter, 1.2 μm PES filters using sterile, single use polypropylene filter cups. Northern pike eDNA analysis used the EluCOI assay published in Olsen et al. (2015). Four qPCR replicates were run for each sample on a BioRad CFX 96‐Touch thermal cycler (Hercules, CA) in the Upper Midwest Environmental Sciences Center laboratory (Onalaska, WI). We tested for PCR inhibition using the approach described in Lor et al. (2020).

#### Site #3: Florida (FL)

We sampled an experimental pond on the USGS Wetland and Aquatic Research Center campus (Gainesville, FL) within the timeframe of 7 November 2022–1 December 2022. At this location, the pond has an elevation of 50 m, a surface area of 3375 m^2^, a volume of 3.8 × 10^6^ L, and average water temperature across the 3 week period was 22°C (U.S. Geological Survey, 2019, Caitlin Beever, U.S. Geological Survey, oral communication, 2025). The eDNA species target was Grass Carp (*Ctenopharyngodon idella*), an aquatic invasive species that was placed in this pond for previous experiments. Sterile 1 L water bottles were filled, emptied, and filled again to collect from the subsurface of the pond. All samples, including negative field controls, were mixed thoroughly and 250 mL were filtered immediately in the USGS Wetland and Aquatic Research Center laboratory through 45 mm diameter, 1.2 μm PES filters, using sterilized PALL filter funnels (Port Washington, NY) and a peristaltic pump. Grass Carp eDNA analysis used the assay described by Hunter et al. (2017). Samples were tested in a multiplex reaction with an IPC on a Bio‐Rad QX200 Droplet Digital (dd) PCR System (Hercules, CA).

#### Site #4: Idaho (ID)

We sampled the Boise River from 2 to 20 August 2022 near downtown Boise, ID. At this location, the river has an elevation of 830 m, width of about 10 m and discharge of 21.67 m^3^/s (U.S. Geological Survey, 2023). The eDNA target was Floater (*Anodonta nuttalliana*), a freshwater mussel native to the Boise River that typically occurs at very low densities. We pumped three 1 L samples of water simultaneously through 45 mm diameter, 5 μm PES filters encased in self‐preserving filter housings using a Smith Root eDNA sampler (Vancouver, WA) with a 2 m sampling pole and trident filter holder. Individual filters were immediately placed into their pre‐labeled sterile storage bags and into a cooler. Filters were transported to the USGS Pacific Northwest Environmental DNA laboratory (Boise, ID) and stored at −20°C in a clean‐room facility within 1 h of collection. Floater eDNA analysis was tested in a multiplex reaction using the assay described in Rodgers et al. (2020) and TaqMan Exogenous Internal Positive Control (IPC) Reagent (ThermoFisher Scientific) on a BioRad CFX384 Touch thermal cycler (Hercules, CA). Three qPCR replicates were run for each sample.

### Methods: Modeling overview

Our modeling framework treats eDNA data collected at a single site over time as originating from one underlying eDNA concentration process, with variation introduced at multiple levels. At the ecological level, site‐level eDNA concentration describes the expected concentration at the site at a given time and reflects ecological and environmental processes operating through time. At the sampling level, the eDNA concentration of water samples collected at the same time varies around this site‐level concentration because eDNA is imperfectly mixed in aquatic environments. At the laboratory level, pipetting, PCR detection, and quantification introduce additional variability through the stochastic allocation of copies to replicates, amplification failure, and measurement error. By explicitly modeling these levels separately, we aim to isolate temporal ecological‐level variation in eDNA concentration from sampling‐ and laboratory‐level variability, enabling more reliable ecological inference and better‐informed temporal sampling designs. Our general approach is to use a temporal hierarchical model (Berliner, 1996), or equivalently, a spatial hierarchical model (Arab et al., 2008) in one dimension, tailored to accommodate the processes of pipetting, eDNA amplification, and measurement (Augustine et al., 2024).

### Methods: Data description

We consider eDNA data collected at a single site over a series of sampling times $t_{i}$ for i=1,…,I, where time is measured in hours. At time $t_{i}$, $J_{i}$ water samples are collected and each water sample is split into $K_{i,j}$ PCR replicates. For each replicate, we observe whether eDNA is detected and, if detected, a quantitative measurement of eDNA copy number. Non‐detections may arise because no target DNA was present in the replicate, amplification failed, or the measurement fell below a lower reporting threshold (Espe et al., 2022; McCall et al., 2014; Sherina et al., 2020). Indices and notation are further described in Appendix S2.

### Methods: Model description

#### Temporal mean structure

We model site‐level eDNA concentration as a latent ecological process evolving through time. Concentration is modeled on the natural log scale to ensure positivity and to reflect multiplicative ecological processes. Let $\mu_{i}^{\text{time}}$ denote the expected log concentration at time $t_{i}$. Then,

$$\log\left(\mu_{i}^{\text{time}}\right)=\beta_{0}+\beta_{1}t_{i},$$

where $\beta_{0}$ is the log concentration at the initial time and $\beta_{1}$ represents a linear temporal trend on the log scale. For the empirical data sets, we fixed $\beta_{1}=0$ because the short duration of the time series limited trend identifiability in the presence of temporal correlation. In the simulation study, $\beta_{1}$ was allowed to vary to evaluate trend detection.

#### Temporal variation and correlation

Next, site‐level eDNA concentration fluctuates through time and may exhibit temporal correlation. We represent these fluctuations by allowing realized log concentrations $C_{i}^{\text{time}}$ to deviate from the mean process and to be correlated across time:

$$\begin{array}{r} \log\left(C^{\text{time}}\right)˜\mathrm{MVN}\left(\log\left(\mu^{\text{time}}\right),\Sigma \right) \end{array},$$

where the covariance matrix Σ follows an exponential correlation structure,

$$\Sigma_{i,j}=\sigma^{{\text{time}}^{2}}\exp\left(-\phi |t_{i}-t_{j}|\right).$$

This model allows concentrations measured close together in time to be more similar than those measured farther apart, reflecting ecological persistence and environmental inertia in eDNA production, transport, and decay. The temporal variance parameter ${\left(\sigma^{\text{time}}\right)}^{2}$ controls the magnitude of temporal fluctuations in site‐level concentration, whereas the range parameter ϕ controls the rate at which temporal correlation decays with increasing time separation. For interpretation, we summarize temporal dependence with the effective range parameter *d*_0_, defined as the time separation beyond which temporal correlation becomes negligible (<0.05; Banerjee et al., 2015). This quantity is directly relevant for temporal sampling design because samples collected closer together than this scale provide partially redundant information and generally should not be treated as independent.

#### Sampling process

Water samples collected at the same time are subject to sampling variability because eDNA is imperfectly mixed in aquatic environments. We model this by allowing sample‐level log concentrations to vary around the realized site‐level concentration:

$$\begin{array}{r} \log\left(C_{i,j}^{\text{samp}}\right)˜\text{Normal}\left(\log\left(C_{i}^{\text{time}}\right),\sigma^{\text{samp}}\right) \end{array},$$

where $C_{i,j}^{\text{samp}}$ is the log concentration of sample *j* collected at time t_*i*_. and $\sigma^{\text{samp}}$ is the sampling SD. This component represents heterogeneity among discrete water samples and is distinct from downstream laboratory measurement error and from temporal variation in site‐level concentration. Separating sampling variability from temporal ecological variation is essential for interpreting short‐term fluctuations in eDNA concentration.

#### Pipetting process

After collection and extraction, each sample of extracted DNA is subsampled into multiple replicates of template DNA (usually 2–3 μL) onto a plate through pipetting for PCR amplification. Because eDNA molecules are discrete and generally at low concentration in solution (i.e., extracted eDNA is stored in ~10 μL of buffer solution that stabilizes DNA against degradation and maintains solubility), this subsampling introduces additional stochastic variation among replicates before amplification occurs. We model this process by assuming that eDNA copies are independently allocated to PCR replicates following a Poisson distribution:

$$N_{i,j,k}˜\text{Poisson}\left(\lambda_{i,j}\right),$$

where $N_{i,j,k}$ is the number of eDNA copies allocated to replicate *k* from sample *j* collected at time $t_{i}$, and

$$\lambda_{i,j}=C_{i,j}^{\text{samp}}\;v_{i,j},$$

where $v_{i,j}$ is a scaling factor determined by the volume of water filtered, elution volume, and aliquot volume used for PCR (Lance et al., 2017; see Appendix S2). These volumes may vary among samples and, if necessary, among replicates.

#### PCR amplification and measurement process

Given the number of eDNA copies allocated to each replicate during pipetting, we then model amplification and quantification as follows. Each PCR replicate undergoes amplification and, if amplification is successful, the number of eDNA copies is quantified. We consider two alternative observation models describing these laboratory processes. The first serves as a baseline model that assumes quantitative measurements from amplified replicates are unbiased. The second extends this baseline model to allow for systematic undermeasurement in a subset of replicates due to reduced PCR efficiency or inhibition. We describe the baseline observation model first, because it captures the essential features of PCR amplification and measurement and provides a natural reference point. We then introduce the undermeasurement model as an extension motivated by lack of fit in some empirical data sets.

#### Baseline amplification and measurement model

Under the baseline model, detection depends on whether at least one eDNA copy allocated during pipetting successfully amplifies. Even when copies are present, amplification can fail stochastically, particularly at low copy numbers. We therefore allow the probability of amplification to increase with the number of eDNA copies present in a replicate (Furlan et al., 2016). Replicates that fail to amplify yield non‐detections. Conditional on successful amplification, replicates yield quantitative measurements of eDNA copy number. These measurements are subject to replicate‐level measurement error but are assumed, under the baseline model, to be unbiased representations of the underlying copy number. Importantly, non‐detections under this model do not necessarily imply absence of eDNA in the original water sample. Non‐detections may arise because no copies were captured in the aliquot during pipetting or because amplification failed despite copies being present.

#### Extended model with stochastic undermeasurement

In some data sets, particularly those generated using qPCR, quantitative measurements from amplified replicates appear systematically lower than expected given the number of copies inferred to be present. This pattern is consistent with reduced PCR efficiency or inhibition and motivates an extension of the baseline observation model. Under the extended observation model, we allow a subset of amplified replicates to be systematically undermeasured. Undermeasurement reduces the expected quantitative measurement relative to the number of copies present in the replicate but does not affect whether amplification occurs. This extension retains the structure of the baseline model while allowing for additional laboratory bias in quantification.

We emphasize that the undermeasurement model does not alter the ecological process model or the sampling and pipetting processes described above. Its purpose is to assess whether allowing for systematic undermeasurement improves model fit and inference about absolute eDNA concentration. Full mathematical details of the amplification probabilities, measurement error distributions, censoring, and undermeasurement formulations are provided in Appendix S2.

### Empirical data analysis

We fit the model separately to the eDNA time series from our four study sites using Markov chain Monte Carlo (MCMC) in the Nimble software (Version 1.0.1; de Valpine et al., 2017) for program R (Version 4.0.5; R Core Team, 2021). For all sites, we fit the baseline observation model and for the MT, WI, and FL sites we additionally fit the extended observation model with undermeasurement, motivated by patterns consistent with reduced PCR efficiency in qPCR data. We did not fit the undermeasurement model to the ID site because detection probability and inferred site‐level concentration were extremely low, limiting identifiability of the additional observation‐process parameters.

For the empirical data sets, we fixed the temporal trend parameter $\beta_{1}=0$ because sampling durations were relatively short and did not show clear monotonic trends, and because estimating trends in the presence of temporal correlation was not supported by the available sample sizes. Lower reporting thresholds were set based on assay‐specific constraints. At the FL site, η was set equal to the limit of blank, defined as the upper 95% bound of measurements from blank samples (0.2524). At the MT, WI, and ID sites, η was set to the copy number corresponding to 50 cycles which was effectively 0 copies in all cases (0.0006, 0.0007, and 0.0006, respectively).

We compared observation models at MT, WI, and FL using conditional Watanabe Information Criteria (WAIC; Watanabe & Opper, 2010) and evaluated lack of fit using posterior predictive checks based on the observation‐model deviance. We report posterior modes and 95% highest posterior density (HPD) intervals. We also summarize the relative contributions of temporal, sampling, and laboratory variability using variance partitioning metrics. Additional details on model specifications, variance partitioning, priors, MCMC settings, and diagnostics are provided in Appendix S2.

### Simulation study

We conducted a simulation study to evaluate how temporal sampling design and modeling choices affect inference about eDNA concentration dynamics. Specifically, we asked: (1) which temporal sampling designs perform well when temporal correlation in eDNA concentration is negligible, (2) how inference is affected when samples are collected closer together in time than the effective range parameter *d*_0_, and temporal correlation is ignored, and (3) which design features are most important when temporal correlation is present and explicitly modeled. We considered three broad classes of ecological scenarios representing different patterns of eDNA concentration through time (Table 2). Two scenarios represented stable populations with relatively high or low mean concentrations, whereas a third scenario represented a population with low initial concentration that increased through time. These scenarios reflect common ecological situations encountered in eDNA monitoring, including stable populations with different abundance levels and populations undergoing an increase.

**TABLE 2:** Summaries of the trend scenarios from the simulation study.

Scenario	$\beta_{0}$	$\beta_{1}$	$\mu^{\text{time}}$	$\bar{\lambda }$	${\bar{p}}^{\mathrm{rep}}$	${\bar{p}}^{\text{samp}}$
S1	6.91	0.000	1000	18.8	0.92	1.00
S2	4.61	0.000	100	1.9	0.45	0.82
S3	3.22	0.001	25–220	1.6	0.38	0.73

*Note*: The trend scenarios are defined by $\beta_{0}$ and $\beta_{1}$ and the implied absolute site concentration, $\mu^{\text{time}}$, which varies through time in S3 only. For each scenario, the realized copy number, and empirical replicate‐level and sample‐level detection probabilities averaged across simulated data sets are $\hat{\lambda }$, ${\bar{p}}^{\mathrm{rep}}$, and ${\bar{p}}^{\text{samp}}$, respectively.

**FIGURE 1:**
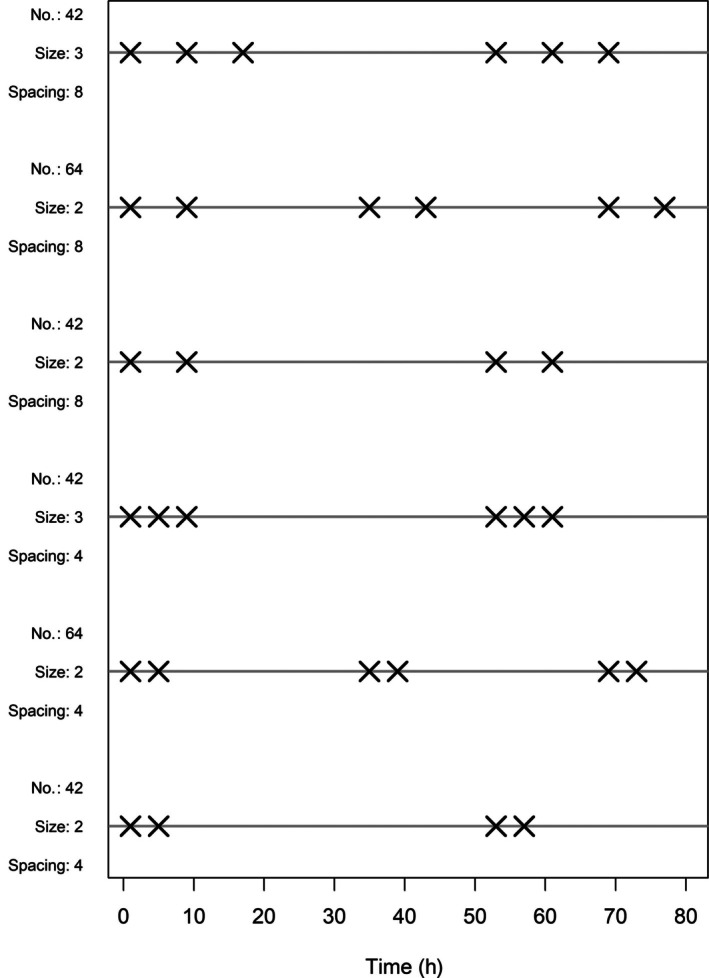
A depiction of the six cluster sampling designs that vary the cluster number (no), cluster size (size), and cluster spacing (spacing) over the first 80 h of the 2176 h in the simulated data sets.

To evaluate temporal sampling design, we compared evenly spaced sample collection through time with clustered sampling designs that combine short‐term replication with longer gaps between sampling periods (e.g., Hoeting et al., 2006; Irvine et al., 2007). All designs assumed a fixed total sampling effort of 142 samples (the capacity of the Monterey Bay Aquarium research Institute FIDO sampler minus two field blanks; Yamahara et al., 2025) distributed over a fixed deployment period, reflecting realistic logistical constraints of autonomous sampling platforms. Clustered designs were intended to provide information on short‐term variability and correlation while retaining coverage across the full deployment period. Sampling designs are summarized in Figure 1 and Table 3.

**TABLE 3:** Summaries of the design scenarios considered in the simulation study with time measured in hours.

Design scenario	Cluster no.	Cluster size	Sampling times	Replicate samples	Spacing within	Spacing between
Equal 1	8	1	8	134	.	272
Equal 2	16	1	16	126	.	136
Equal 3	32	1	32	110	.	68
Equal 4	64	1	64	78	.	34
Equal 5	128	1	128	14	.	17
Equal 6	142	1	142	0	.	15
Cluster 1	42	2	84	58	4	48
Cluster 2	64	2	128	14	4	30
Cluster 3	42	3	126	16	4	44
Cluster 4	42	2	84	58	8	44
Cluster 5	64	2	128	14	8	26
Cluster 6	42	3	126	16	8	36

*Note*: “Equal” scenarios have equally spaced sampling times and “Cluster” scenarios have equally spaced clusters with equally spaced sampling times within clusters. We provide the cluster number, cluster size (sampling times per cluster), and the number of sampling times (the product of cluster number and cluster size). We further provide the number of temporal replicate samples (replicate samples), and the within (spacing within) and between (spacing between) cluster spacing. All scenarios use a total of 142 samples.

We also varied the degree of temporal correlation in site‐level eDNA concentration. In low‐correlation scenarios, the effective range parameter was short relative to the minimum sampling interval, and in high‐correlation scenarios, the effective range was long relative to sampling intervals. For high‐correlation scenarios, we compared models that explicitly accounted for temporal correlation with models that ignored it, allowing us to quantify the consequences of model misspecification—inflated Type I error rates.

For each combination of ecological scenario, sampling design, and correlation scenario, we simulated multiple data sets and fit the corresponding models using the baseline observation model. We evaluated performance using three metrics: (1) precision of parameter estimates, (2) statistical power to detect temporal trends when present, and (3) Type I error rates for trend detection when no trend was present. Power and Type I error rates were defined based on whether 95% posterior intervals for the trend parameter excluded zero. We also examined interval coverage as an indicator of whether posterior uncertainty was underestimated, particularly in scenarios where temporal correlation was ignored. Detailed simulation parameter values, sampling schedules, computational settings, and example of simulated time series are provided in Appendix S3. Both the empirical data and simulation analyses are available in Augustine et al. (2026).

## RESULTS

### Empirical data analysis

The undermeasurement observation model was favored by WAIC for all three data sets where it was considered (MT, WI, and FL sites, Table 4), indicating improved predictive performance relative to the unbiased observation model. Posterior predictive checks did not indicate strong lack of fit at the overall level for either observation model at any site. However, at the sample level, the unbiased observation model showed substantially more extreme deviations at the MT site and, to a lesser extent, the WI site. These deviations were largely associated with very low quantitative measurements and were greatly reduced under the undermeasurement observation model. At the FL site, both models showed little evidence of lack of fit. No inhibition was detected in the lab at the WI or FL sites, but 11 samples indicated inhibition at the MT site, four of which were also identified as poorly fitting in the unbiased measurement model by the posterior predictive checks. Detailed Bayesian *p*‐values and sample‐level diagnostics are provided in Appendix S4: Table S4 and the graphical goodness‐of‐fit assessment is in Appendix S4: Figure S1.

**TABLE 4:** Parameter point estimates for the three empirical data sets from the null (M1) and undermeasurement (M2) models with Watanabe Information Criteria (WAIC) summaries.

	MT		WI		FL	
	M1	M2	M1	M2	M1	M2
$\sigma^{y}$	1.58	0.27	1.10	0.37	0.08	0.08
$\beta_{0}$	5.23	6.10	4.21	4.48	6.90	6.89
$p^{\mathrm{amp}}$	1.00	0.53	0.80	0.59	0.36	0.41
$p^{\mathrm{mix}}$	.	1.00	.	0.03	.	0.99
$\psi_{0}$	.	−2.82	.	−6.46	.	−1.37
$\psi_{1}$	.	0.21	.	0.21	.	3.50
ϕ	0.017	0.011	0.197	0.233	0.013	0.023
$d_{0}$	176	273	15	13	231	130
$\sigma^{\text{samp}}$	0.62	0.79	0.61	0.53	0.93	0.94
$\sigma^{\text{time}}$	0.73	0.72	1.01	0.96	0.88	0.87
VPC rep	0.22	0.06	0.20	0.20	0.01	0.01
VPC samp	0.39	0.64	0.34	0.28	0.72	0.72
VPC time	0.39	0.31	0.46	0.52	0.27	0.27
WAIC	769.31	309.52	657.43	460.05	−295.50	−343.40
lppd	−358.49	−38.51	−253.43	−115.45	307.83	339.55
pWAIC	26.17	116.26	75.29	114.57	160.08	167.85

*Note*: Point estimates are posterior modes except for the variance partition coefficients (VPCs), which are posterior means to ensure they sum to one. $\beta_{0}$ is the intercept on the natural log scale. We report the WAIC, the log pointwise predictive density (lppd) and the effective number of parameters (pWAIC). The effective range, $d_{0}$ is derived from ϕ and is in units of hours. Interval estimates are provided in Appendix S4: Tables S1 and S2.

Abbreviations: FL, Florida; MT, Montana; WI, Wisconsin.

Parameter estimates from the undermeasurement model indicated substantial undermeasurement at the MT site, moderate undermeasurement at the WI site, and minimal undermeasurement at the FL site (Table 4). At the MT site, all replicates were estimated to be undermeasured $\left({\hat{p}}^{\mathrm{mix}}=1.00\right)$ and the expected measurement of a replicate with one copy was estimated to be 0.06, which increased slowly with the number of copies in a replicate. At the WI site, undermeasurement was inferred for a smaller subset of replicates $\left({\hat{p}}^{\mathrm{mix}}=0.03\right)$ and the expected measurement of a replicate with one copy was 0.002, also increasing slowly as copy number increases. At the FL site, all replicates were estimated to be undermeasured $\left({\hat{p}}^{\mathrm{mix}}=1.00\right)$ and the expected measurement of a replicate with one copy was 0.02, but the thinning rate increased very fast with the number of copies in a replicate–a replicate with two copies would be undermeasured by 11% and a replicate with three copies would effectively not be undermeasured. For reference, there were only two replicates that measured less than three copies. These patterns are visualized in Appendix S4: Figure S2. Accounting for undermeasurement led to lower estimates of amplification probability and measurement error, and higher estimates of site‐level concentration at the MT and WI sites, whereas parameter estimates at the FL site were largely unchanged.

The MT, WI, and FL sites were estimated to have considerable temporal variability, sampling variability, and temporal correlation, although the temporal correlation parameter ϕ was weakly identifiable and was estimated imprecisely (Table 4, Appendix S4: Tables S1 and S2). The lower bounds of the 95% CI for ϕ at the MT and WI sites were nearly the same as their respective prior lower bounds (Appendix S4: Tables S1 and S2), indicating that these time series may have been too short to estimate ϕ without positive bias given this level of uncertainty. Variance partitioning indicated that sampling variability contributed more to overall variation than replication variability at the MT and FL sites, whereas variance components at the WI site could not be clearly distinguished due to uncertainty (Appendix S4: Tables S1 and S2). Overall uncertainty in variance components was high across sites.

**FIGURE 2:**
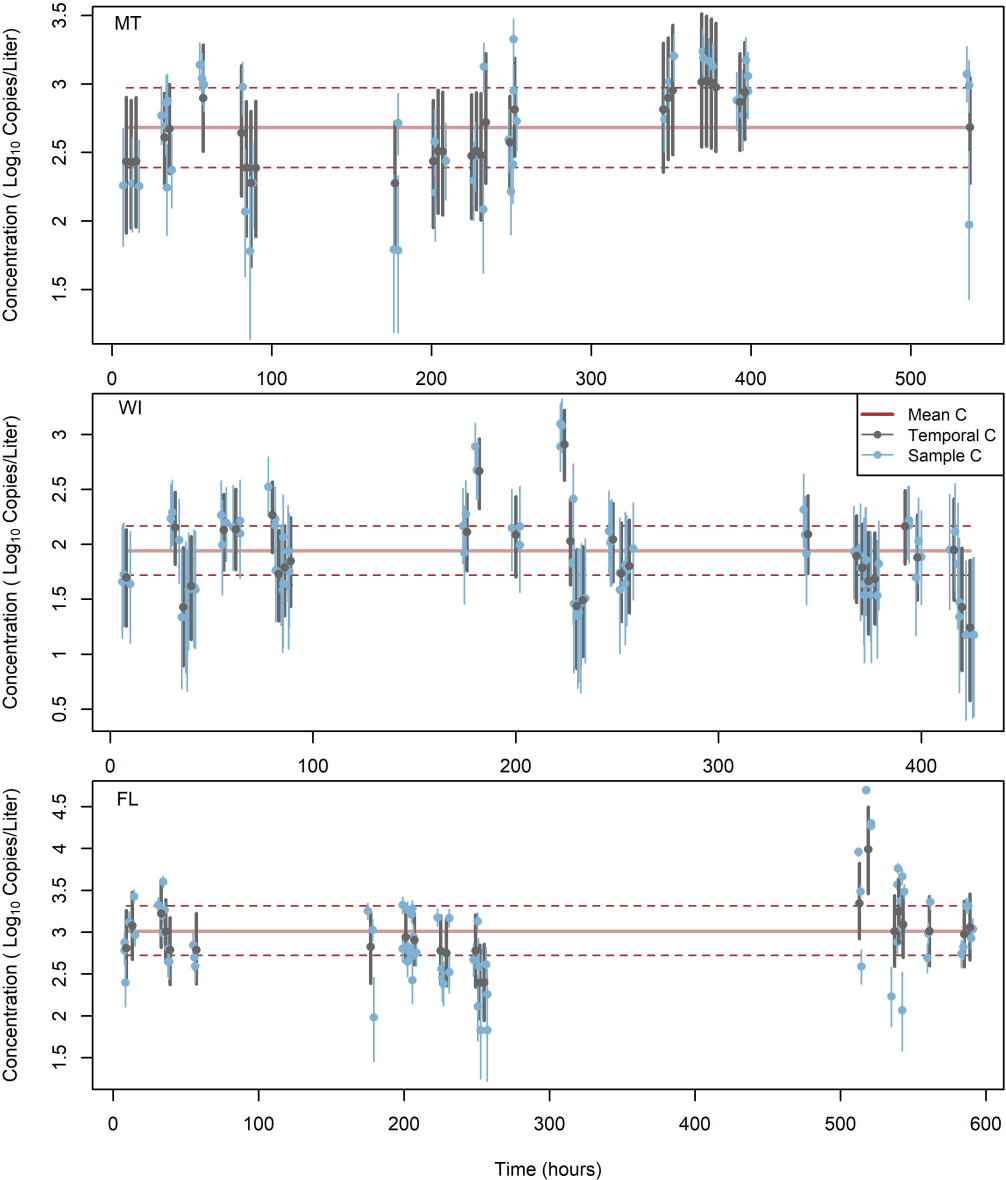
Plots of the estimated expected site concentration (Mean C), the temporal concentration at sampled times (Temporal C), and the concentration of each sample (Sample C) for the Montana (MT), Wisconsin (WI), and Florida (FL) empirical data sets. Point estimates are posterior modes and interval estimates are 95% highest posterior density (HPD) intervals. The analogous plot for the Idaho site is in Appendix S4.

At the ID site, the ϕ parameter was unidentifiable and the temporal and sampling SDs were estimated near zero, although the 95% CI upper bounds did not rule out considerable variation (Appendix S4: Table S3). We attribute the imprecision of parameter estimates for this data set to the low estimated site concentration (1.8 copies/L) resulting in a low detection probability and sparse quantitative measurements. The largest source of variation at the ID site was estimated to be the replication variability (Appendix S4: Table S3), at least in part due to the low site concentration (see Appendix S2: Variance Partitioning). However, we believed that this model was overparameterized for this data set and did not consider the estimates to be reliable. The estimated log_10_ expected site concentration, temporal concentrations at the measured times, and the sample concentrations can be visualized in Figure 2 (MT, WI, and FL sites) and Appendix S4: Figure S3 (ID site).

### Simulation study

#### Design considerations for negligible temporal correlation

**FIGURE 3:**
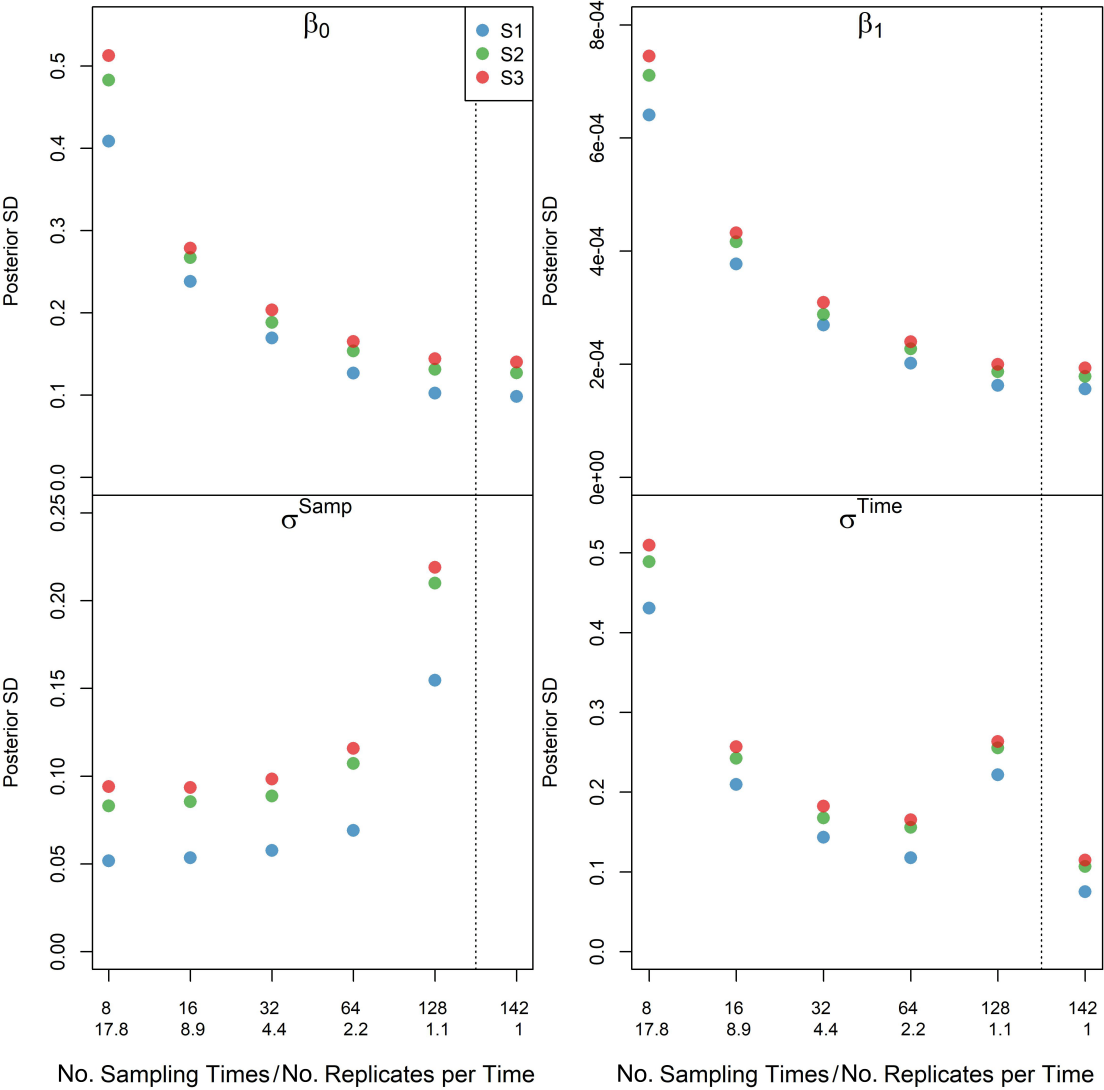
Simulation study: Posterior SDs (averaged over 100 simulated data sets per scenario) for site concentration parameters, $\beta_{0}$ and $\beta_{1}$, and the sampling and temporal SD parameters, $\sigma^{\text{samp}}$ and $\sigma^{\text{time}}$, from the equally spaced design scenarios where $d_{0}=10$ (low temporal correlation) and ϕ is not modeled. The vertical dotted line separates the scenarios with 142 sample times, no temporal replicates, and with the sampling variance pooled with the estimated temporal variance.

**FIGURE 4:**
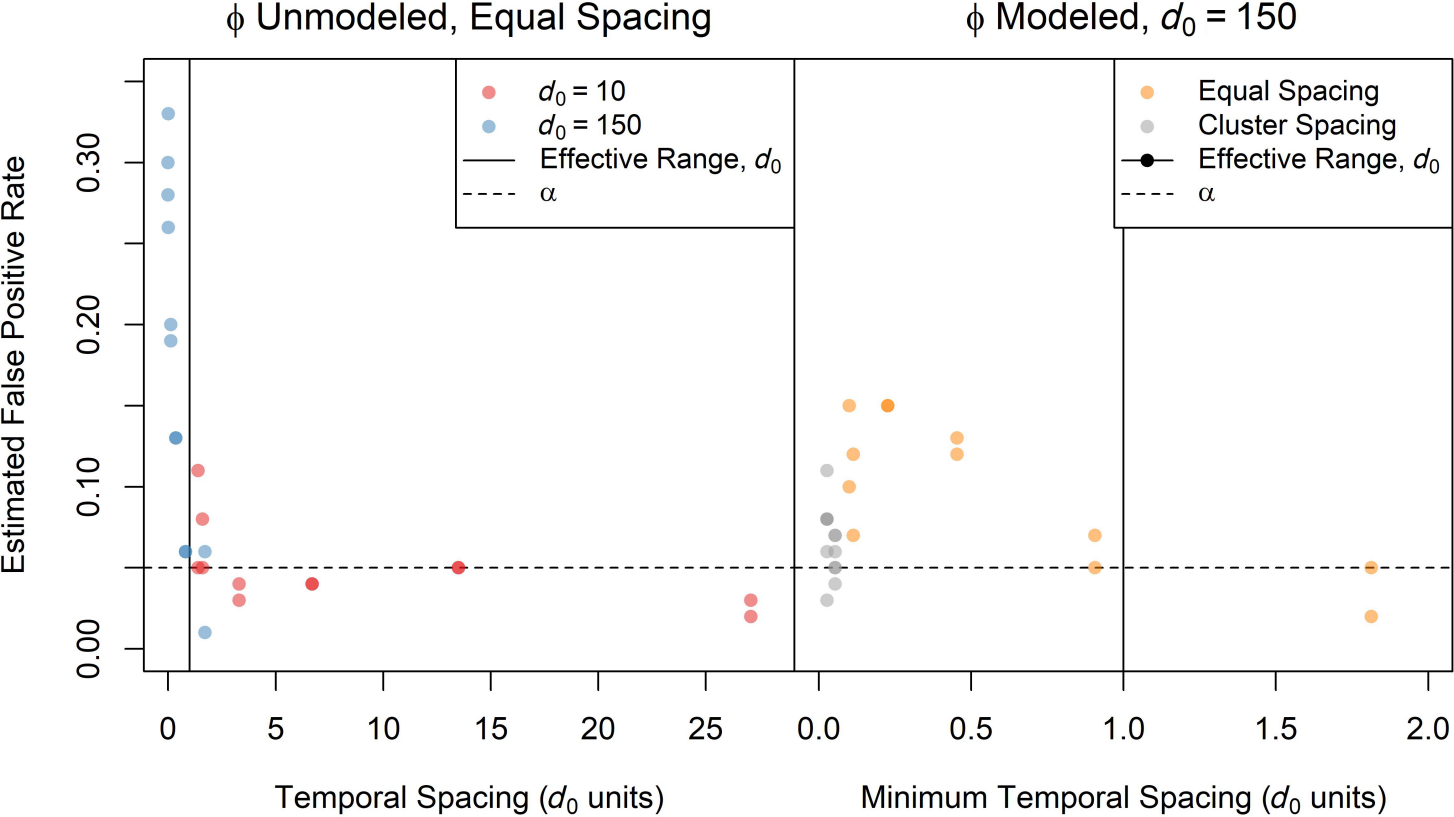
Simulation study: Estimated Type I error rates as a function of temporal spacing in units of the effective range, $d_{0}$. On the left are the scenarios where ϕ was not modeled, which we only considered for the equally spaced design scenarios. These scenarios included two levels of $d_{0}$, 10 and 150 h with 1 $d_{0}$ unit, indicated with a vertical black line, being the spacing where temporal correlation is approximately negligible. The target frequentist Type I error rate, α was 0.05. On the right are the equally spaced and cluster scenarios where ϕ was modeled, which we only considered for $d_{0}=150$.

**FIGURE 5:**
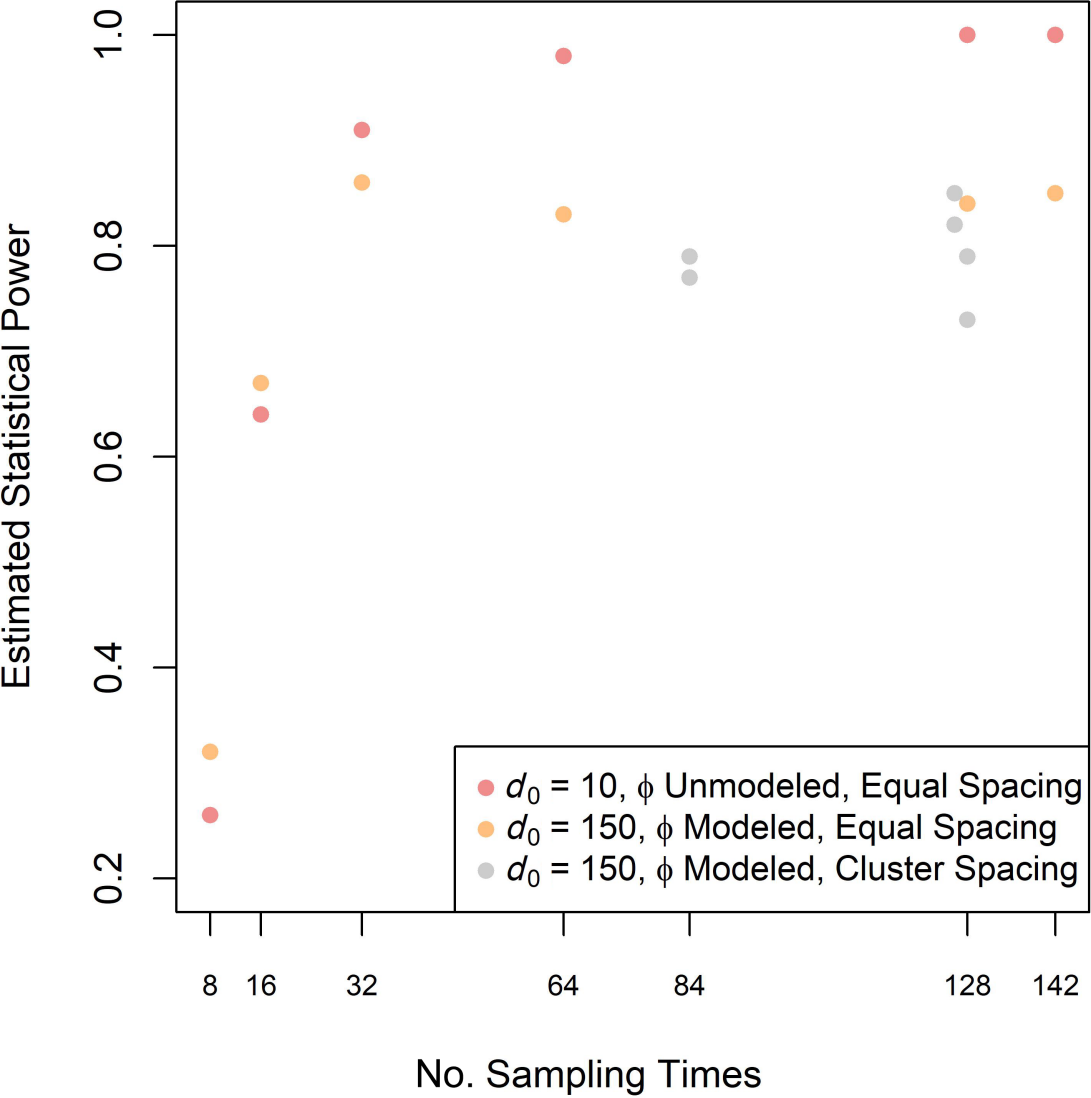
Simulation study: Estimated statistical power for trend scenario S3 where (1) temporal correlation was low ($d_{0}=10$) and not modeled because equally spaced times were negligibly correlated, (2) temporal correlation was high ($d_{0}=150$) and modeled for equally spaced designs, and (3) temporal correlation was high and modeled for cluster spaced designs.

In general, we found that spreading the samples over more sampling times increased the precision of the trend parameter estimates, as measured by their posterior SDs, but with diminishing returns (Figure 3, Appendix S5: Table S4). As a caveat, the trend parameter estimates from the equally spaced scenario with 142 sampling times and no temporal replication were similarly precise as the analogous scenario with 128 sampling times and limited temporal replication (Figure 3, Appendix S5: Table S4). Type I error rates were roughly nominal, except perhaps scenario S1 with 142 sampling times where the temporal spacing was closest together and approaching the effective range (estimated FPR of 0.11, Figure 4, Appendix S5: Table S1). Statistical power in the S3 trend scenario also increased as the number of sampling times increased (Figure 5; Appendix S5: Table S1). There were diminishing returns in this relationship, but the statistical power was nearly 1.0 x 64 sampling times, leaving little room for improvement with more sampling times.

By contrast, the sampling SD $\sigma^{\text{samp}}$ was estimated more precisely when the samples were allocated to fewer sampling times, implying more temporal replication (Figure 3, Appendix S5: Table S4). Among scenarios with temporal replication (<142 sampling times), the temporal SD $\sigma^{\text{time}}$ was estimated most precisely with intermediate numbers of sampling times and levels of temporal replication (Figure 3, Appendix S5: Table S5). The most precise estimate of $\sigma^{\text{time}}$ was in the scenario with 142 sampling times and no temporal replication, where we did not partition the temporal and sampling variances. The relative bias of parameter estimates excluding variance components was generally less than ±3%, and relative bias for variance components was generally less than ±5% (Appendix S5: Table S3). For all parameters, coverage was roughly nominal (Appendix S5: Table S5).

#### Effect of temporal correlation on inference

As described above, when the sampling times were spaced further apart than $d_{0}$ as they were in all equally spaced scenarios with $d_{0}=10$, the temporal correlation was negligible and inference was not strongly affected. Type I error rates were roughly nominal (Figure 4) and coverage was roughly nominal for all parameters (Appendix S5: Table S5). Conversely, when the temporal spacing was less than $d_{0}$ as in all equally spaced scenarios with $d_{0}=150$, except for the subscenario with eight sampling times, the Type I error rate was inflated (Figure 4) and coverage was less than nominal for $\beta_{0}$ and $\beta_{1}$ (Appendix S5: Table S5).

Modeling the temporal correlation considerably reduced the Type I error rate, though it was still somewhat higher than nominal in some scenarios (Figure 4, Appendix S5: Table S2). The Type I error rates were closer to nominal in the cluster spacing scenarios compared to the equally spaced scenarios when the spacing was less than 0.5 $d_{0}$ (Figure 4). Modeling temporal correlation also reduced statistical power (Figure 5, Appendix S5: Table S2), and in the presence of temporal correlation, statistical power was similar in all designs with 32 or more sampling times (Figure 5, Appendix S5: Tables S1 and S2). Modeling the temporal correlation also increased the coverage of $\beta_{0}$ and $\beta_{1}$ to closer to nominal, with a few exceptions (Appendix S5: Table S5 vs. Table S8).

#### Design considerations for non‐negligible temporal correlation

**FIGURE 6:**
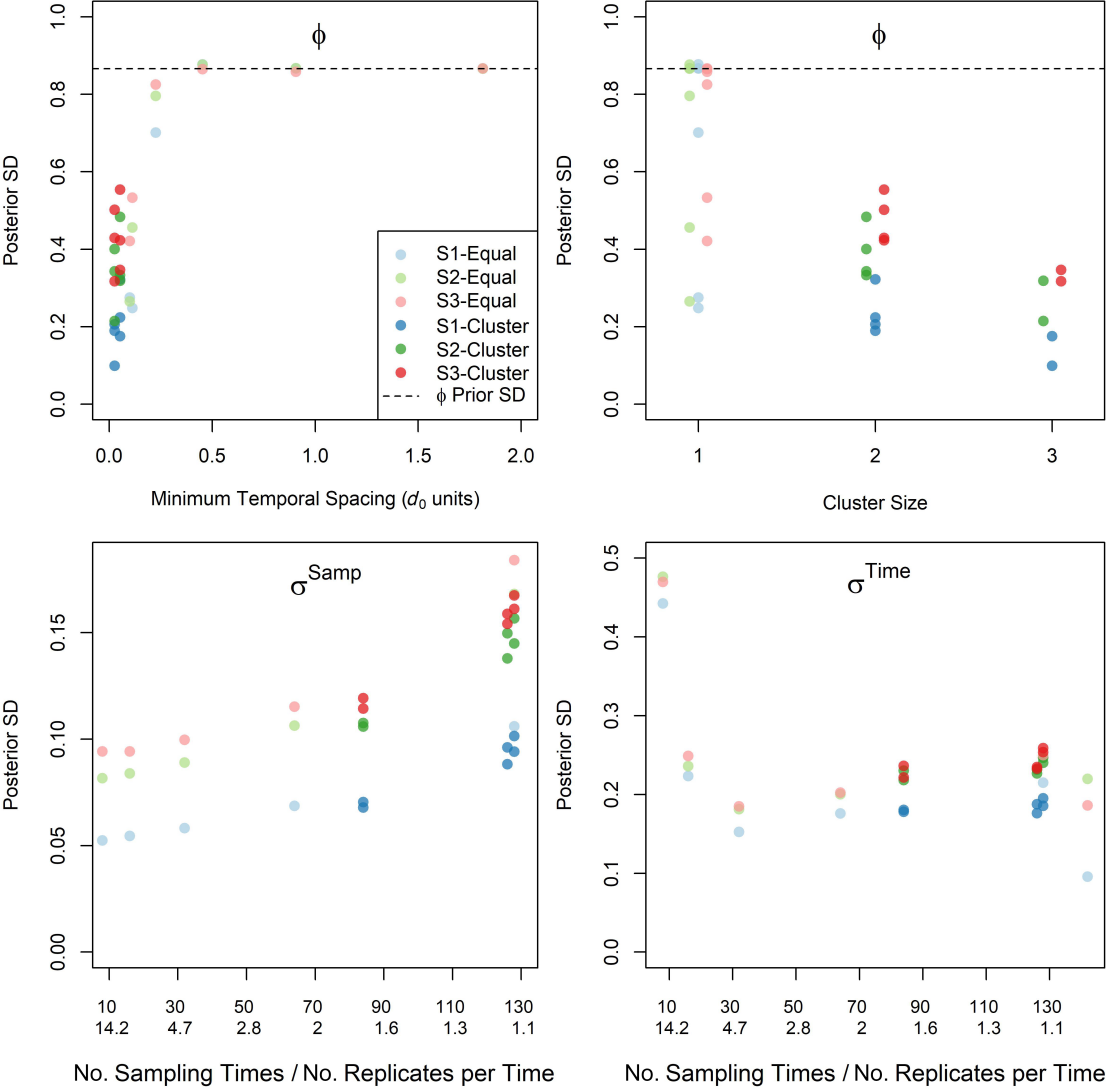
Simulation study: Posterior SDs (averaged over 100 simulated data sets per scenario) for temporal correlation parameter ϕ, and sampling and temporal SD parameters, $\sigma^{\text{samp}}$ and $\sigma^{\text{time}}$ as a function of design factors and trend scenarios (S1–S3). We plot the posterior SDs of ϕ versus the minimum cluster spacing in $d_{0}$ units (top left) and versus cluster size (number of sampling times per cluster, top right). We indicate the prior SD with a dotted line. We plot $\sigma^{\text{samp}}$ and $\sigma^{\text{time}}$ posterior SDs versus the mean number temporal replicates per sampling time and total number of samples.

When temporal correlation was non‐negligible and we modeled it, the statistical power increased with the number of sampling times up to 32, beyond which the power was relatively stable around 0.8 (Figure 5, Appendix S5: Table S2). Unlike scenarios with negligible temporal correlation, increasing the number of sampling times over 32 did not improve the precision of the $\beta_{0}$ and $\beta_{1}$ estimates as measured by the posterior SD (Appendix S5: Table S7). However, similar to negligible temporal correlation scenarios, the precision of the sampling SD $\sigma^{\text{samp}}$ was greater in scenarios with the fewest sampling times and the most temporal replication, and the precision of the temporal SD $\sigma^{\text{time}}$ was highest in scenarios with intermediate numbers of sampling times and temporal replication (Figure 6, Appendix S5: Table S7).

We were unable to estimate the temporal correlation parameter, ϕ, without modest to severe positive bias (32%–7499%, Appendix S5: Table S6) due to weak identifiability in at least some simulated data sets across all scenarios. However, we were able to largely reduce the greater than nominal modeled Type I error rates even when estimating ϕ with substantial positive bias (Figure 4, Appendix S5: Table S2). The precision of our ϕ estimates as measured by the posterior SD increased as the minimum temporal spacing decreased, although it is not clear whether the precision would continue to increase at minimum temporal spacings less than the smallest we considered (1 h) or if we already reached a lower asymptote (Figure 6, Appendix S5: Table S7). When the minimum temporal spacing in $d_{0}$ units was greater than 0.5 $d_{0}$, the posterior SD was estimated to be roughly equivalent to the prior SD, indicating a near complete lack of identifiability (Figure 6, Appendix S5: Table S7). We also found that ϕ was estimated more precisely as the number of sampling times per cluster (cluster size) increased, although this effect was modest (Figure 6, Appendix S5: Table S7). Finally, ϕ was estimated less precisely as the average site concentration (average of $\mu^{\text{time}}$) in the trend scenarios decreased from 1000 copies/L in S1, to 100 in S2, and 89.7 in S3 (Figure 6, Appendix S5: Table S7) and with less bias in the trend scenario S1 compared to S2 and S3 (Appendix S5: Table S6).

## DISCUSSION

There is a growing call to expand the scale and scope of eDNA monitoring to support national and international conservation efforts (Goodwin et al., 2024). This expansion will likely involve more temporally intensive sampling than in the past, given increasing evidence that extensive sampling is often necessary to achieve reasonable precision in eDNA concentration estimates due to substantial sampling and measurement variability (Shelton et al., 2022; Yates et al., 2023). To meet this increasing demand, we expect eDNA autosamplers (George et al., 2024; Sepulveda, Birch, et al., 2020) to become an increasingly important tool because they can be programmed to collect samples at any time of the day and thus reduce or free up human labor for other tasks (Sepulveda, Birch, et al., 2020). Regardless of sampling method, we need appropriate statistical models to ensure ecological inferences are reliable. The modeling framework we developed here and the study design guidance provide a step in this direction.

To our knowledge, we provide the first empirical estimates of the variance component parameters of eDNA sampling in the presence of temporal correlation. This information is vital for understanding the relative magnitudes of sources of ecological variation and correlation and for study design. Our data sets with 48–108 total samples were not sufficient to produce precise estimates of these parameters, particularly the temporal correlation parameter ϕ. Nonetheless, there appears to be substantial sampling and temporal variation at all three sites and substantial temporal correlation at the MT and WI sites. With respect to temporal variation, the temporal site concentration point estimates ranged roughly from one‐half to a full order of magnitude, depending on the data set (Figure 2). We may have actually underestimated the magnitude of temporal correlation (analogously, overestimated ϕ) at the MT and WI sites due to the constraints we placed on the prior for ϕ so that ϕ and $\sigma^{\text{time}}$ were jointly identifiable given the total length of the survey, because the 95% CI lower bounds for ϕ were equal to the prior lower bounds.

The sampling variation was estimated to be a larger contributor to the total variance than the temporal variance at the MT and FL sites. This high sampling variance limits the precision of ecological parameter estimates and reduces our ability to distinguish among competing ecological hypotheses (i.e., during model selection). In addition, the relatively large sampling variance makes estimating the variance components parameters less accurate and precise (large nugget‐to‐sill ratio; Irvine et al., 2007). We attribute this large sampling variance to the fact that eDNA particles are not well mixed in water bodies (or are clumped; Yates et al., 2023), and furthermore, cell bound or free eDNA masses can vary in size (Snyder et al., 2023; Yates et al., 2023). We hypothesize that substantial sampling variation contributes to the inconsistent field results when relating eDNA concentration to organismal abundance or biomass (Sepulveda et al., 2021; Yates et al., 2019).

In our simulation study, we found that estimating site‐level concentration means or trends, as well as temporal variation and correlation parameters, is challenging given the empirical magnitude of sampling variability and the sample sizes we considered. However, incorporating temporal correlation into the model improved the reliability of detecting concentration trends by reducing the Type I error rate, particularly when using clustered sampling designs.

Consistent with previous work, cluster designs better estimated the temporal correlation parameter (Delmelle, 2021; Hoeting et al., 2006; Irvine et al., 2007). Conversely, when temporal correlation is low or samples can be spaced farther apart than the effective range parameter (when temporal correlation is effectively negligible), equally spaced sampling designs provide greater statistical power to detect concentration trends.

Ultimately, the optimal design depends on the specific objective of the survey and the true parameter values, particularly the variance components. We expect that both temporal variability and correlation are hard to predict in advance, as they are unlikely to remain stationary even over short windows of time as we assumed. Therefore, pilot studies may be necessary to inform appropriate study design. A precautionary strategy may be to anticipate that temporal correlation may be nonnegligible, use a clustered design, and then use these data to inform adaptive sampling strategies. Including at least some temporal replication—multiple samples collected at approximately the same time—can help better partition temporal and sampling variance.

To keep our analyses tractable, we did not consider temporal covariates, such as discharge or turbidity (George et al., 2024; Morrison et al., 2023), that could help explain the temporal variation and correlation in eDNA concentration. If these are included in an appropriately specified model, the inflated Type I error rates in high temporal correlation scenarios may be reduced, depending on the remaining magnitudes of temporal variation and correlation. For example, Morrison et al. (2023) found that environmental covariates were able to explain half of the daily variability in eDNA samples, and covariates may also explain the temporal correlation. If nonnegligible temporal correlation remains after controlling for available covariates, inflated Type I error rates may still occur for both eDNA concentration trends and covariate effects. Therefore, study designs should enable acceptable estimation of ϕ even when temporal covariates are available.

We observed evidence of poor goodness‐of‐fit for the three data sets we assessed (MT, WI, and FL sites) as judged by samples with smaller posterior predictive, sample‐level Bayesian *p*‐values and graphical goodness‐of‐fit plots. Lack of fit was pronounced at the MT site and moderate at the WI site (both qPCR datasets), whereas it was minor for the ddPCR dataset from FL and only evident when two or fewer copies were in a replicate. We hypothesized this lack of fit was due to systematic undermeasurement and WAIC favored the undermeasurement models for all three data sets. The undermeasurement models estimated substantially higher site and sample concentrations for the two qPCR data sets as expected, but the difference was very minor for the ddPCR data set (Appendix S4: Figure S2). One possible explanation for these results is that undermeasurement in the qPCR datasets stemmed from reduced efficiency relative to the standard curve, which was calibrated using sterile water with a composition different from field samples that may contain inhibitory compounds (Ruijter et al., 2021; Sepulveda, Hutchins, et al., 2020). Our lab inhibition tests did find evidence of inhibition in 11 of the 48 samples at MT where the lack‐of‐fit of the unbiased measurement model was strongest.

The undermeasurement models likely rely too strongly on simplistic and hard to verify parametric assumptions for unbiased parameter estimation and reliable inference. For example, inhibition or other sources of undermeasurement are likely more variable than our assumption that replicates with the same number of copies are equally likely to be undermeasured and by the same average magnitude. Further, the constraint of only being able to subject a single aliquot to PCR once prevents the statistical replication that would facilitate more complicated models of inhibition and more statistical power to detect lack‐of‐fit. However, the unbiased measurement model will also not provide reliable inference in the presence of substantial undermeasurement, so we chose to use the parameter estimates from the undermeasurement models for the simulation analysis.

Our analysis of empirical datasets indicates that temporal variation and correlation in eDNA concentrations across diverse aquatic environments can be substantial. Furthermore, our simulation study demonstrates that when samples are collected too close together in time, Type I error rates can be substantially inflated beyond the nominal level. Under these conditions, ecologists are more likely to draw incorrect conclusions about temporal trends in eDNA concentration or the role of specific temporal covariates in explaining eDNA concentration variation over time. Given the difficulty of estimating temporal variation and correlation parameters in the presence of large sampling variance (Irvine et al., 2007), even with 142 total samples, accounting for temporal correlation in practice may be prohibitively expensive for many projects. Ultimately, quantitative eDNA measurements (copy number and concentration) carry very little information per sample in the presence of large environmental and measurement variability, and variation in efficiencies can cause some eDNA sample concentrations to be undermeasured more than others. Binary detection data contain even less information per sample under the same conditions, making the use of temporal models even more challenging (see Appendix S6 for a comparison). Given these challenges, temporal covariates that explain temporal variability and correlation should be recorded when possible and incorporated into models for quantitative and binary eDNA data (e.g., George et al., 2024; Morrison et al., 2023). Even so, an unknown magnitude of temporal variation and correlation may remain, so inferences should be interpreted with the understanding that Type I error rates–the probability of concluding there is an effect when none is present–may still be inflated.

## CONFLICT OF INTEREST STATEMENT

The authors declare no conflicts of interest.

## Supporting information


Appendix S1:

Appendix S2:

Appendix S3:

Appendix S4:

Appendix S5:

Appendix S6:

## Data Availability

Data and code (Augustine et al., 2026) are available in Dryad at https://doi.org/10.5061/dryad.g4f4qrg19.
