# Supplementary material for: Accounting for temporal variation and correlation in environmental DNA sampling can improve ecological inferences
Source: Ecol Appl. 2026 Aug 4;36(5):e70290. doi: 10.1002/eap.70290 (PMC13438698; doi:10.1002/eap.70290)
Supplement: Supplementary file 1 [file EAP-36-e70290-s001.pdf]

# Appendix S6

# Accounting for temporal variation and correlation in environmental DNA sampling can improve ecological inferences

Ben C. Augustine, Patrick R. Hutchins, Margaret E. Hunter, Christopher M. Merkes, David S. Pilliod, Stephen F. Spear, Caitlin E. Beaver, Scott D. George, Devin N. Jones-Slobodian, Adam J. Sepulveda

*Ecological Applications*

Disclaimer: Any use of trade, firm, or product names is for descriptive purposes only and does not imply endorsement by the U.S. Government

## Binary Data

Here, we provide an exploration of an observation process that uses the binary detection data only.

## Methods - Binary Data

We define $y^{det}_{i,j,k}$ to be the detection outcome, taking value 1 if detected and 0 otherwise. Previously, Furlan et al. (2016) used the following assumptions we use in our model for quantitative data to estimate sample concentrations from binary data:

$$N_{i,j,k} \sim \text{Poisson}(\lambda_{i,j})$$

$$p^{N}_{i,j,k} = 1 - (1 - p^{amp})^{N_{i,j,k}}$$

with $p^{amp}$ estimated from dilution assays. However, we expect $p^{amp}$ estimated from dilution assays using sterile water to be higher than that of field samples, and any failed detections due to censoring instead of failed amplification would introduce negative bias. Therefore, we consider a

less ambitious model that only considers that nondetections arise when zero copies are allocated to a replicate. The replicate-level detection probability follows from the assumed Poisson allocation of copies across replicates

$$p_{i,j,k}^{det} = 1 - \exp(-\lambda_{i,j})$$

and the observation model is Bernoulli:

$$y_{i,j,k}^{det} \sim \text{Bernoulli}(p_{i,j,k}^{det}).$$

This model will underestimate concentrations when $p^{amp} < 1$, but can provide unbiased estimates of the concentration trend if the allocation of 0 copies to a replicate and failed amplification are the only source of nondetections ($\eta = 0$). In this situation, the expected number of copies that will amplify is $\lambda'_{i,j} = \lambda_{i,j} p^{amp}$, the ratio of the expected values of counts and abundance does not change as concentration changes, and we can estimate the rate of change without bias (Link and Sauer 1998). Note, this result does not hold if nondetections may also occur due to a lower bound on possible measurements ($\eta > 0$) or if the model for amplification deviates from equation 7.

For binary data, we considered both the low and high correlation scenarios where $\phi$ was not modeled for equally spaced design scenarios to compare to the same quantitative data scenarios. We also considered the high correlation scenario where we model $\phi$ for cluster design scenarios. Because trend scenario S1 produced very uninformative detection data with a detection probability very close to 1.0, we did not consider it in the high correlation, cluster design scenarios.

## Results - Binary vs. Quantitative Data

We found that under our assumptions about how nondetections arise, models using the binary data provided similar parameter estimates and inferences about the temporal trend as models using the quantitative data, except for trend scenario S1 where detection probability was nearly 1.0, leaving very little information about model parameters in the binary data. In scenarios with negligible temporal correlation, the estimates of the trend parameters from binary data, $\beta_0$ and $\beta_1$, in trend scenarios S2 and S3 were nearly as precise as those from quantitative data, but the sampling and temporal standard deviation parameters were estimated with a greater loss of precision over the estimates from the quantitative data (Figure S1, Table S4). The estimates from the binary data also had similarly low levels of bias in trend scenarios S2 and S3, except for the site concentration intercept, $\beta_0$, which was increasingly underestimated as the site concentrations decline from trend scenario S1 - S3, due to the increasing occurrence of failed amplification events (Figure S2, Table S3). Regarding inference, in low temporal correlation scenarios, the binary data models had similar statistical power in trend scenario S3 compared to the quantitative data models, roughly nominal Type I error rates when the temporal spacing was greater than the effective range, and inflated Type I error rates when the temporal spacing was less than the effective range, though less so than seen using the quantitative data (Figure S3, Table S1).

In scenarios with non-negligible temporal correlation ($d_0 = 150$) where it is modeled, the binary data models had similar statistical power in trend scenario S3 compared to quantitative data models, and similarly low Type I error rates in trend scenario S2 (Figure S3, Table S2). Similar

to the negligible temporal correlation scenarios, the estimates of the trend parameters, $\beta_0$ and $\beta_1$ were of similar precision to those from the quantitative data (Table S4), the sampling and temporal standard devation parameters and the temporal correlation parameter were estimated with less precision (Table S4), and the concentration intercept, $\beta_0$ was estimated with negative bias (Table S3).

In sum, the binary data provides similar inference about trends in concentration when there is no lower bound on measurement ($\eta = 0$) and when concentration is in a range where detection probability is not very close to 0 or 1, but concentration is systematically underestimated proportional to the per copy amplification probability, $p^{amp}$. Even when $\eta > 0$, we expect inference about $\beta_1$ to reliably indicate the direction in which concentration is changing, but $\beta_1$ will no longer correspond exactly to the exponential rate of growth in concentration. This model is arguably more ecologically realistic than multiscale occupancy (Dorazio and Erickson 2018; Stratton, Sepulveda, and Hoegh 2020) that is the current state of the art for eDNA detection data. Quantitative data provided more precise estimates of the variance components, though precision from binary data could be increased by increasing the number of technical replicates per sample (we considered 5 in the simulation study and our empirical data sets used 4-5).

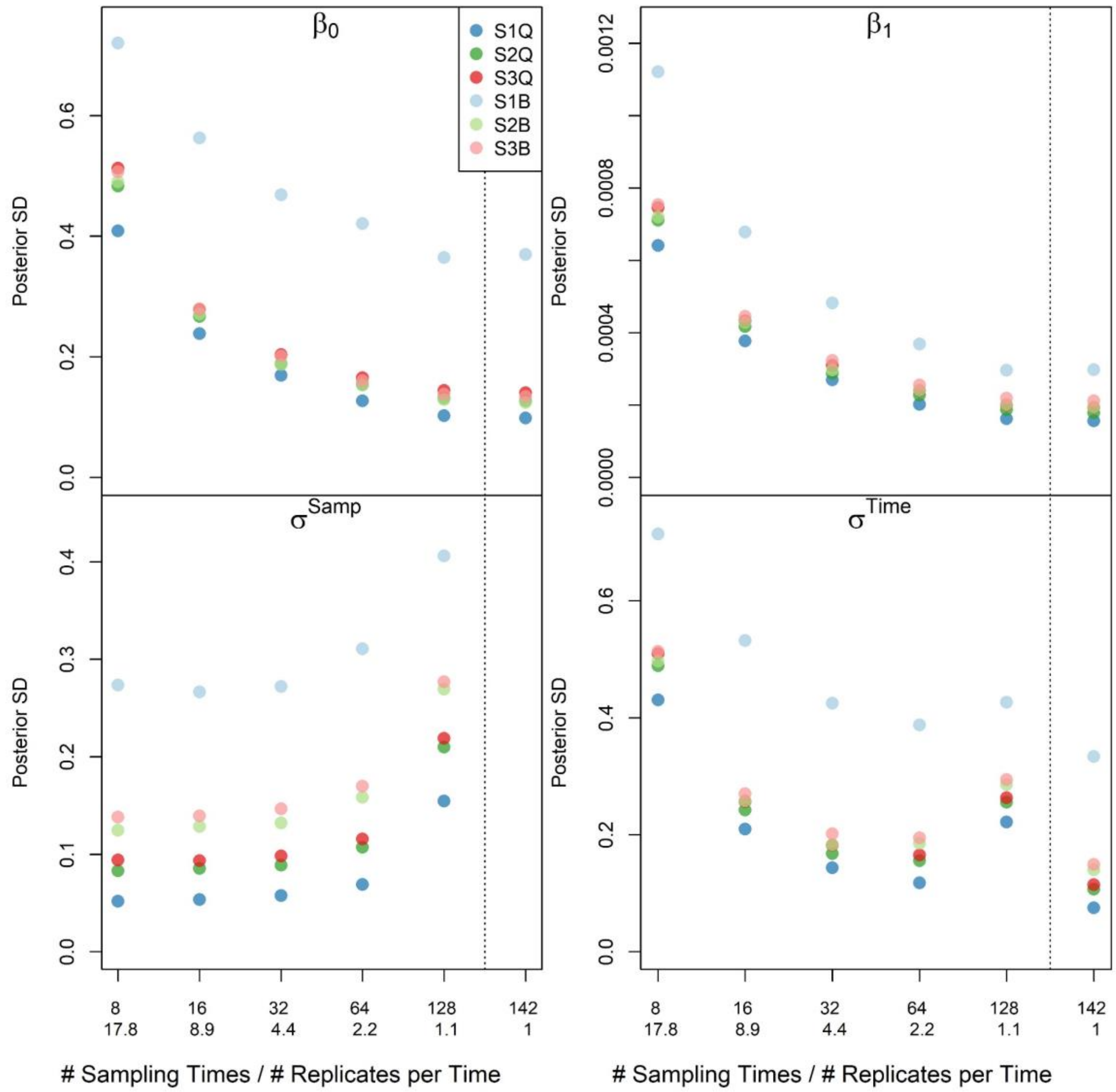


**Figure S1**. *Posterior standard deviations (averaged over 100 simulated data sets per scenario) for site concentration parameters, $\beta_0$ and $\beta_1$, and the sampling and temporal standard deviation parameters, $\sigma^{samp}$ and $\sigma^{time}$, from the equally spaced design scenarios where $\mathrm{d}_0 = 10$ (low temporal correlation) and $\phi$ is not modeled. Scenarios using quantitative data are labeled with "Q" and scenarios with binary data are labeled with "B". The vertical dotted line separates the scenarios with 142 sample times, no temporal replicates, and with the sampling variance pooled with the estimated temporal variance.*

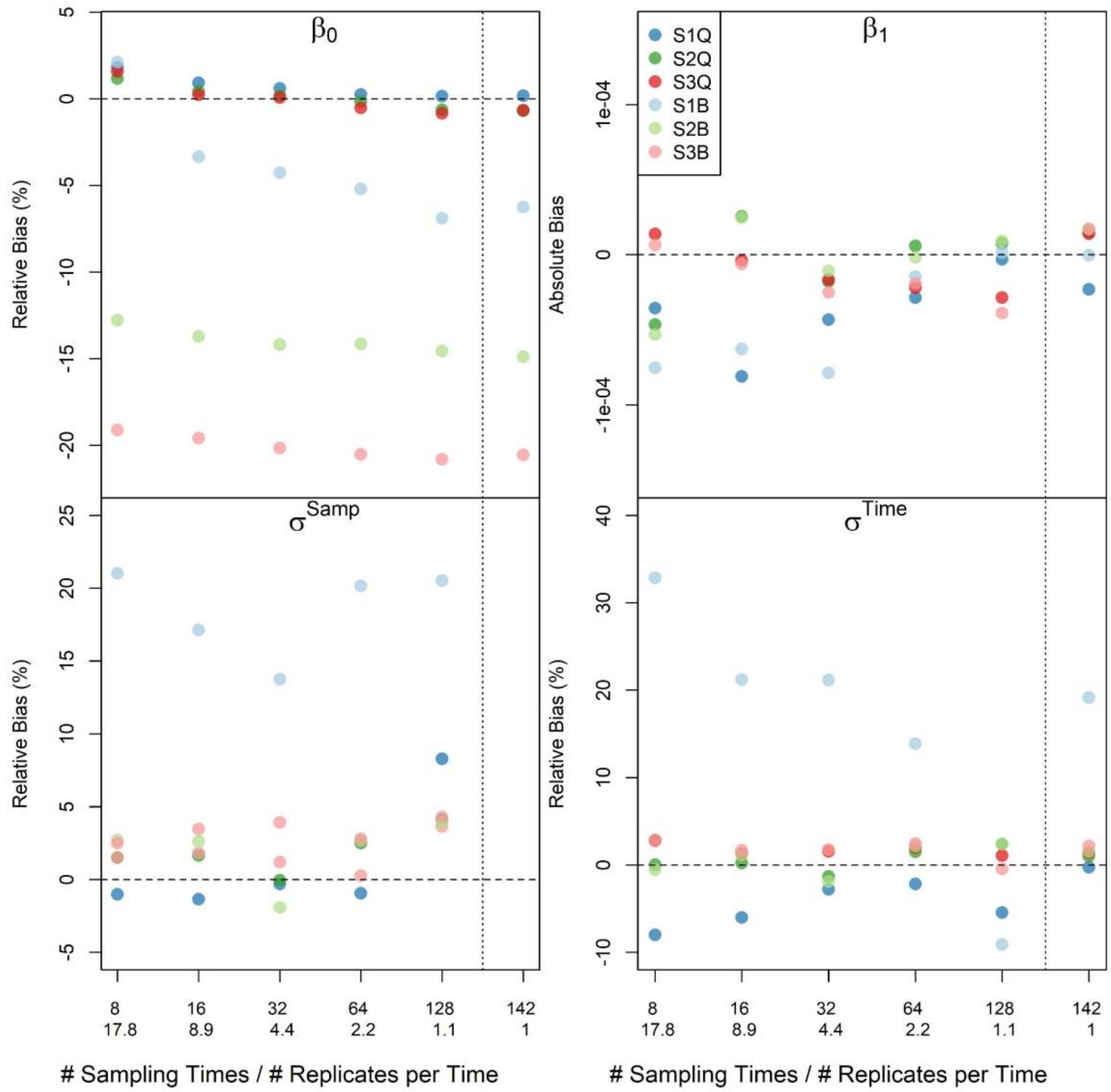


**Figure S2**. *Bias for site concentration parameters, $\beta_0$ and $\beta_1$, and the sampling and temporal standard deviation parameters, $\sigma^{samp}$ and $\sigma^{time}$, from the equally spaced design scenarios where $d_0 = 10$ (low temporal correlation) and $\phi$ is not modeled. Scenarios using quantitative data are labeled with "Q" and scenarios with binary data are labeled with "B". The vertical dotted line separates the scenarios with 142 sample times, no temporal replicates, and with the sampling variance pooled with the estimated temporal variance. Bias is presented as relative bias except for $\beta_1$, which is presented as absolute bias to avoid dividing by zero in trend scenarios S1 and S2. For $\beta_1$ +/- 1$e^{-4}$ corresponds to 10% of the simulated value in the increasing trend scenario, S3.*

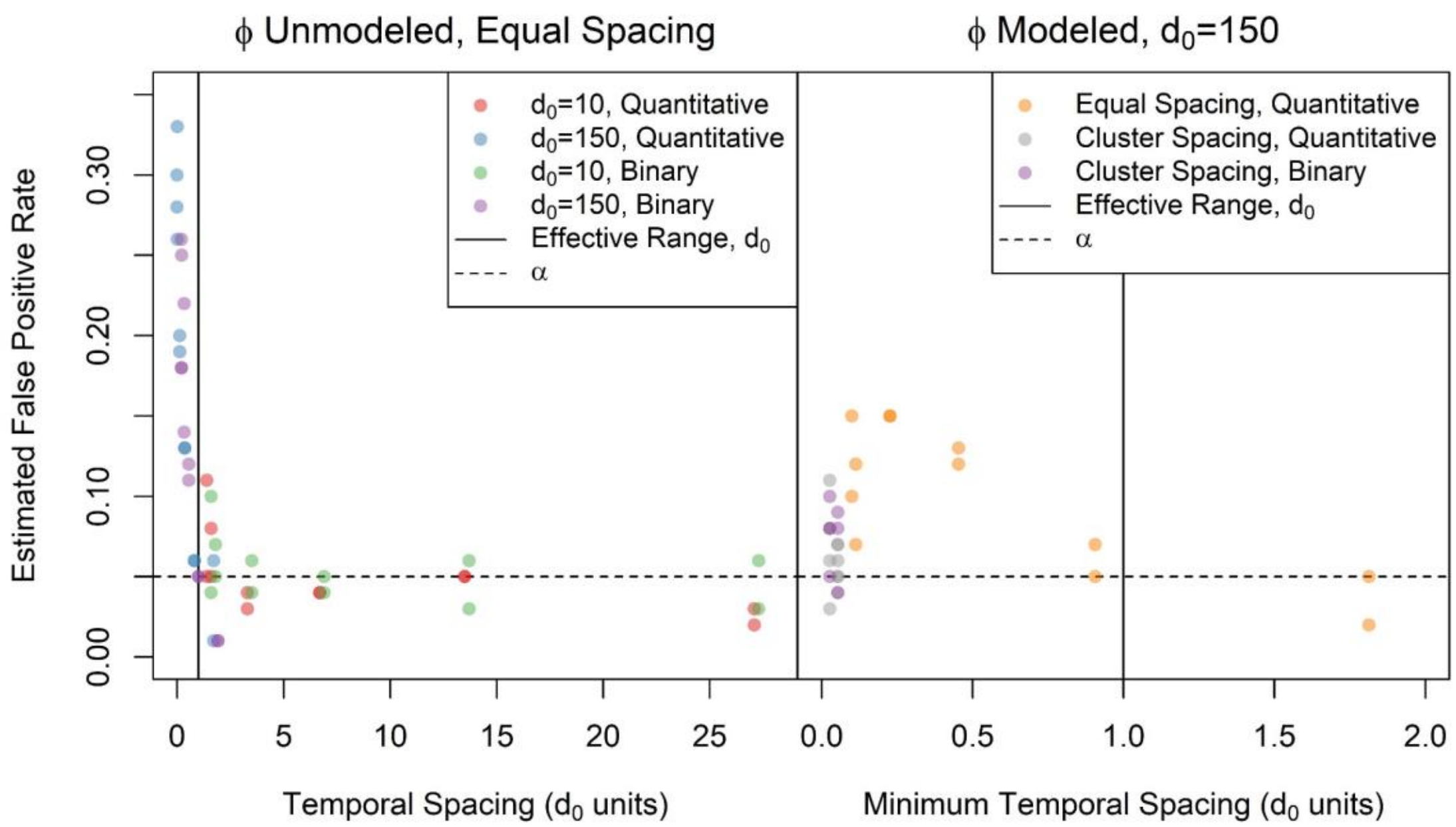


**Figure S3**. *Estimated Type I error rates as a function of temporal spacing in units of the effective range,* $d_0$. *On the left are the scenarios where* $\phi$ *was not modeled, which we only considered for the equally spaced design scenarios. These scenarios included two levels of* $d_0$, *10 and 150 hours with 1* $d_0$ *unit, indicated with a vertical black line, being the spacing where temporal correlation is approximately negligible. The target frequentist Type I error rate,* $\alpha$ *was 0.05. On the right are the scenarios where* $\phi$ *was modeled, which we only considered for* $d_0 = 150$. *Equally spaced design scenarios were not considered for binary data.*

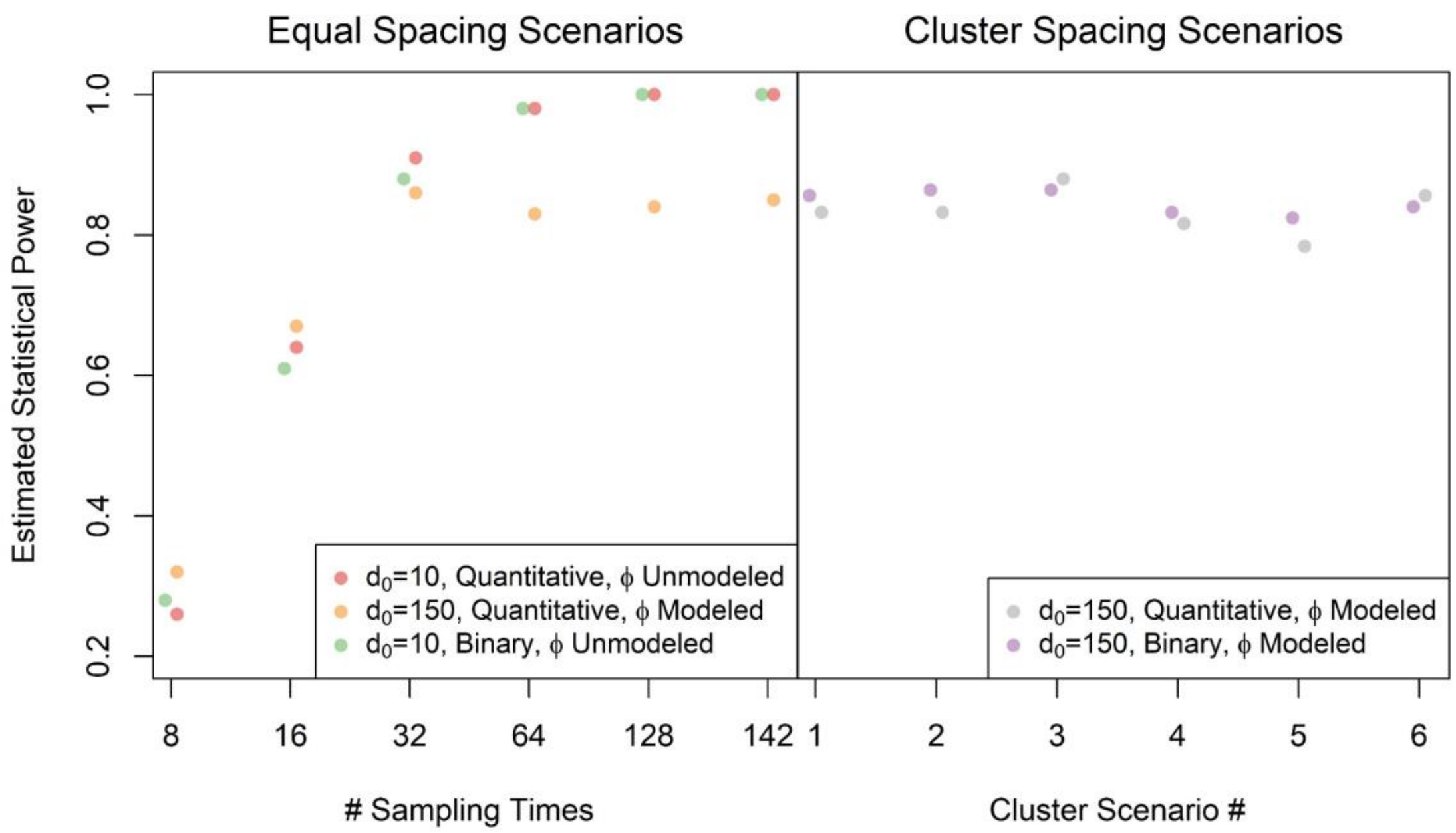


**Figure S4**. *Estimated statistical power for trend scenario S3 excluding scenarios with high temporal correlation ($d_0 = 150$) where temporal correlation was not modeled. On the left are equal spacing scenarios and on the right are cluster spacing scenarios.*

**Table S1**. *Estimated Type I error rate (FPR) for stable trend scenarios (S1 and S2) and statistical power (Power) for increasing trend scenarios (S3) from the equally spaced design scenarios. Trend scenarios are further subdivided by $d_0$ scenarios ($d_0$ Level) determining the level of temporal correlation, scenarios where we ignore or model the temporal correlation (Model $\phi$), data type scenarios where we use quantitative (Q) or binary (B) data, and scenarios varying the number of sampling times. We did not consider scenarios for binary data where we modeled $\phi$ in equally spaced design scenarios.*

| Trend Scenario | FPR/ Power | $d_0$ Level | Model $\phi$ | # Sampling Times 8 | 16 | 32 | 64 | 128 | 142 |
|---|---|---|---|---|---|---|---|---|---|
| S1 | FPR | 10 | No | 0.06 | 0.03 | 0.04 | 0.04 | 0.07 | 0.04 |
| S1 | FPR | 150 | No | 0.01 | 0.05 | 0.12 | 0.14 | 0.18 | 0.18 |
| S2 | FPR | 10 | No | 0.03 | 0.06 | 0.05 | 0.06 | 0.05 | 0.10 |
| S2 | FPR | 150 | No | 0.01 | 0.05 | 0.11 | 0.22 | 0.25 | 0.26 |
| S3 | Power | 10 | No | 0.28 | 0.61 | 0.88 | 0.98 | 1.00 | 1.00 |
| S3 | Power | 150 | No | 0.31 | 0.71 | 0.87 | 0.94 | 0.98 | 0.96 |

**Table S2**. *Estimated Type I error rate (FPR) for stable trend scenarios (S1 and S2) and statistical power (Power) for increasing trend scenarios (S3) across cluster design scenarios. The cluster design parameters for scenarios enumerated 1-6 can be found in Table 3. Quantitative data is indicated with "Q" and binary data with "B".*

| Trend | FPR/ | Cluster Scenario # |
|---|---|---|

| Scenario | Power | 1 | 2 | 3 | 4 | 5 | 6 |
|---|---|---|---|---|---|---|---|
| S2 | FPR | 0.10 | 0.08 | 0.05 | 0.08 | 0.04 | 0.09 |
| S3 | Power | 0.82 | 0.83 | 0.83 | 0.79 | 0.78 | 0.80 |

**Table S3**. *Relative bias of model parameters in each simulation scenario with binary data, equally spaced sampling times, and the temporal correlation parameter $\phi$ not modeled. S1 indicates higher, stable concentration, S2 indicates lower, stable concentration, and S3 indicates lower, increasing concentration. "L" indicates the scenario with lower temporal correlation ($d_0 = 10$) and "H" indicates the scenario with higher temporal correlation ($d_0 = 150$). Sampling time (design) scenarios are 8, 16, 32, 64, 128, and 142. Scenarios with 142 sampling times did not estimate the sampling standard deviation and the temporal standard deviation estimate is compared to the standard deviation corresponding to the pooled sampling and temporal variance. Relative bias for $\beta_1$ is not reported because the true value was 0.*

| Scenario | $\beta_0$ | $\beta_1$ | $\sigma^{samp}$ | $\sigma^{time}$ |
|---|---|---|---|---|
| S1LB-8 | 2.12 | . | 21.01 | 32.85 |
| S1LB-16 | -3.35 | . | 17.14 | 21.19 |
| S1LB-32 | -4.27 | . | 13.75 | 21.17 |
| S1LB-64 | -5.20 | . | 20.15 | 13.87 |
| S1LB-128 | -6.89 | . | 20.52 | -9.10 |
| S1LB-142 | -6.26 | . | . | 19.16 |
| S2LB-8 | -12.77 | . | 2.72 | -0.53 |
| S2LB-16 | -13.73 | . | 2.61 | 1.14 |
| S2LB-32 | -14.19 | . | -1.92 | -1.89 |
| S2LB-64 | -14.15 | . | 2.67 | 2.15 |
| S2LB-128 | -14.57 | . | 3.92 | 2.40 |
| S2LB-142 | -14.90 | . | . | 1.54 |
| S3LB-8 | -19.13 | 0.64 | 2.50 | 2.75 |
| S3LB-16 | -19.59 | -0.63 | 3.47 | 1.70 |
| S3LB-32 | -20.16 | -2.51 | 3.92 | 1.74 |
| S3LB-64 | -20.53 | -1.93 | 2.79 | 2.45 |
| S3LB-128 | -20.82 | -3.88 | 4.28 | -0.46 |
| S3LB-142 | -20.56 | 1.69 | . | 2.17 |
| S1HQ-8 | -0.61 | . | 19.78 | 44.76 |
| S1HQ-16 | -4.18 | . | 20.07 | 26.89 |
| S1HQ-32 | -6.37 | . | 14.28 | 13.48 |
| S1HQ-64 | -5.84 | . | 15.11 | 12.50 |
| S1HQ-128 | -6.11 | . | 13.31 | 3.07 |
| S1HQ-142 | -7.13 | . | . | 10.38 |
| S2HQ-8 | -12.10 | . | 2.65 | 3.59 |
| S2HQ-16 | -13.26 | . | 1.44 | -1.62 |
| S2HQ-32 | -14.05 | . | -0.02 | -3.16 |
| S2HQ-64 | -14.30 | . | 0.47 | -4.06 |

| | | | | |
|---|---|---|---|---|
| S2HQ-128 | -14.54 | . | -0.25 | -5.18 |
| S2HQ-142 | -14.17 | . | . | -1.79 |
| S3HQ-8 | -19.03 | 6.28 | 1.73 | -3.20 |
| S3HQ-16 | -20.30 | 1.77 | 1.51 | -2.98 |
| S3HQ-32 | -20.74 | 1.44 | 3.63 | -3.51 |
| S3HQ-64 | -21.67 | 3.02 | 1.61 | -4.42 |
| S3HQ-128 | -21.07 | -0.20 | 4.01 | -6.23 |
| S3HQ-142 | -22.05 | -1.49 | . | -0.50 |

**Table S4**. *Posterior standard deviations of model parameters in each simulation scenario with binary data, equally spaced sampling times, and the temporal correlation parameter $\phi$ not modeled. S1 indicates higher, stable concentration, S2 indicates lower, stable concentration, and S3 indicates lower, increasing concentration. "L" indicates the scenario with lower temporal correlation ($\mathrm{d}_0 = 10$) and "H" indicates the scenario with higher temporal correlation ($\mathrm{d}_0 = 150$). Sampling time (design) scenarios are 8, 16, 32, 64, 128, and 142. Scenarios with 142 sampling times did not estimate the sampling standard deviation and the temporal standard deviation estimate is compared to the standard deviation corresponding to the pooled sampling and temporal variance. Relative bias for $\beta_1$ is not reported because the true value was 0.*

| Scenario | $\beta_0$ | $\beta_1$ | $\sigma^{samp}$ | $\sigma^{time}$ |
|---|---|---|---|---|
| S1LB-8 | 0.7201 | 0.0011 | 0.2736 | 0.7141 |
| S1LB-16 | 0.5627 | 0.0007 | 0.2666 | 0.5319 |
| S1LB-32 | 0.4684 | 0.0005 | 0.2721 | 0.4249 |
| S2LB-64 | 0.4208 | 0.0004 | 0.3108 | 0.3879 |
| S2LB-128 | 0.3645 | 0.0003 | 0.4059 | 0.4265 |
| S2LB-142 | 0.3697 | 0.0003 | . | 0.3337 |
| S3LB-8 | 0.4901 | 0.0007 | 0.1247 | 0.4964 |
| S3LB-16 | 0.2706 | 0.0004 | 0.1284 | 0.2580 |
| S3LB-32 | 0.1871 | 0.0003 | 0.1322 | 0.1830 |
| S1LB-64 | 0.1530 | 0.0002 | 0.1585 | 0.1853 |
| S1LB-128 | 0.1290 | 0.0002 | 0.2693 | 0.2857 |
| S1LB-142 | 0.1242 | 0.0002 | . | 0.1400 |
| S2LB-8 | 0.5066 | 0.0008 | 0.1382 | 0.5135 |
| S2LB-16 | 0.2790 | 0.0004 | 0.1395 | 0.2702 |
| S2LB-32 | 0.2011 | 0.0003 | 0.1466 | 0.2016 |
| S3LB-64 | 0.1600 | 0.0003 | 0.1697 | 0.1948 |
| S3LB-128 | 0.1378 | 0.0002 | 0.2768 | 0.2944 |
| S3LB-142 | 0.1337 | 0.0002 | . | 0.1492 |
| S1HQ-8 | 0.7387 | 0.0012 | 0.2504 | 0.7272 |
| S1HQ-16 | 0.5619 | 0.0007 | 0.2505 | 0.5345 |
| S1HQ-32 | 0.4471 | 0.0005 | 0.2633 | 0.3984 |
| S1HQ-64 | 0.4096 | 0.0004 | 0.3011 | 0.3722 |
| S1HQ-128 | 0.3780 | 0.0003 | 0.4049 | 0.4329 |

| | | | | |
|---|---|---|---|---|
| S1HQ-142 | 0.3608 | 0.0003 | . | 0.3187 |
| S2HQ-8 | 0.5081 | 0.0007 | 0.1245 | 0.5076 |
| S2HQ-16 | 0.2640 | 0.0004 | 0.1271 | 0.2511 |
| S2HQ-32 | 0.1854 | 0.0003 | 0.1333 | 0.1837 |
| S2HQ-64 | 0.1464 | 0.0002 | 0.1562 | 0.1817 |
| S2HQ-128 | 0.1260 | 0.0002 | 0.2601 | 0.2779 |
| S2HQ-142 | 0.1213 | 0.0002 | . | 0.1375 |
| S3HQ-8 | 0.4812 | 0.0007 | 0.1394 | 0.4919 |
| S3HQ-16 | 0.2684 | 0.0004 | 0.1398 | 0.2632 |
| S3HQ-32 | 0.1951 | 0.0003 | 0.1477 | 0.1994 |
| S3HQ-64 | 0.1563 | 0.0003 | 0.1699 | 0.1942 |
| S3HQ-128 | 0.1338 | 0.0002 | 0.2766 | 0.2931 |
| S3HQ-142 | 0.1331 | 0.0002 | . | 0.1479 |

**Table S5**. *Coverage (95%) of model parameters in each simulation scenario with binary data, equally spaced sampling times, and the temporal correlation parameter $\phi$ not modeled. S1 indicates higher, stable concentration, S2 indicates lower, stable concentration, and S3 indicates lower, increasing concentration. "L" indicates the scenario with lower temporal correlation ($d_0 = 10$) and "H" indicates the scenario with higher temporal correlation ($d_0 = 150$). Sampling time (design) scenarios are 8, 16, 32, 64, 128, and 142. Scenarios with 142 sampling times did not estimate the sampling standard deviation and the temporal standard deviation estimate is compared to the standard deviation corresponding to the pooled sampling and temporal variance. Relative bias for $\beta_1$ is not reported because the true value was 0.*

| Scenario | $\beta_0$ | $\beta_1$ | $\sigma^{samp}$ | $\sigma^{time}$ |
|---|---|---|---|---|
| S1LB-8 | 0.86 | 0.94 | 0.90 | 0.84 |
| S1LB-16 | 0.83 | 0.97 | 0.92 | 0.90 |
| S1LB-32 | 0.77 | 0.96 | 0.90 | 0.93 |
| S2LB-64 | 0.75 | 0.96 | 0.90 | 0.94 |
| S2LB-128 | 0.69 | 0.93 | 0.96 | 0.97 |
| S2LB-142 | 0.73 | 0.96 | . | 0.87 |
| S3LB-8 | 0.81 | 0.97 | 0.95 | 0.95 |
| S3LB-16 | 0.39 | 0.94 | 0.94 | 0.94 |
| S3LB-32 | 0.03 | 0.95 | 0.99 | 0.95 |
| S1LB-64 | 0.01 | 0.94 | 0.85 | 0.94 |
| S1LB-128 | 0.00 | 0.95 | 0.93 | 0.98 |
| S1LB-142 | 0.00 | 0.90 | . | 0.98 |
| S2LB-8 | 0.79 | 0.99 | 0.97 | 0.95 |
| S2LB-16 | 0.38 | 0.98 | 0.93 | 0.97 |
| S2LB-32 | 0.11 | 0.95 | 0.92 | 0.95 |
| S3LB-64 | 0.00 | 0.91 | 0.93 | 0.95 |
| S3LB-128 | 0.00 | 0.94 | 0.94 | 0.94 |
| S3LB-142 | 0.00 | 0.93 | . | 0.96 |

| | | | | |
|---|---|---|---|---|
| S1HQ-8 | 0.91 | 0.99 | 0.89 | 0.79 |
| S1HQ-16 | 0.87 | 0.95 | 0.91 | 0.88 |
| S1HQ-32 | 0.71 | 0.88 | 0.88 | 0.93 |
| S1HQ-64 | 0.70 | 0.86 | 0.94 | 0.92 |
| S1HQ-128 | 0.65 | 0.82 | 0.91 | 0.96 |
| S1HQ-142 | 0.61 | 0.82 | . | 0.96 |
| S2HQ-8 | 0.88 | 0.99 | 0.95 | 0.96 |
| S2HQ-16 | 0.36 | 0.95 | 0.93 | 0.96 |
| S2HQ-32 | 0.07 | 0.89 | 0.93 | 0.97 |
| S2HQ-64 | 0.04 | 0.78 | 0.93 | 0.91 |
| S2HQ-128 | 0.00 | 0.75 | 0.98 | 0.88 |
| S2HQ-142 | 0.02 | 0.74 | . | 0.93 |
| S3HQ-8 | 0.74 | 0.95 | 0.95 | 0.95 |
| S3HQ-16 | 0.37 | 0.93 | 0.95 | 0.96 |
| S3HQ-32 | 0.12 | 0.92 | 0.91 | 0.95 |
| S3HQ-64 | 0.01 | 0.77 | 0.95 | 0.92 |
| S3HQ-128 | 0.02 | 0.83 | 0.95 | 0.92 |
| S3HQ-142 | 0.02 | 0.70 | . | 0.91 |

**Table S6**. *Relative bias of model parameters in each simulation scenario with binary data and the temporal correlation parameter $\phi$ modeled. S2 indicates lower, stable concentration, and S3 indicates lower, increasing concentration (S1 not considered). "H" indicates the scenario with higher temporal correlation ($d_0 = 150$; scenarios with $d_0 = 10$ and $\phi$ modeled were not considered). Cluster design scenarios are C1-6, described in Table 3. Relative bias for $\beta_1$ is not reported because the true value was 0.*

| Scenario | $\beta_0$ | $\beta_1$ | $\phi$ | $\sigma^{samp}$ | $\sigma^{time}$ |
|---|---|---|---|---|---|
| S2HQ-C1 | -14.33 | . | 269.36 | -4.14 | 2.01 |
| S2HQ-C2 | -14.52 | . | 341.05 | -7.08 | 5.67 |
| S2HQ-C3 | -14.30 | . | 225.95 | -10.22 | 10.19 |
| S2HQ-C4 | -13.92 | . | 297.58 | -3.13 | 3.17 |
| S2HQ-C5 | -14.40 | . | 250.19 | -4.45 | 11.76 |
| S2HQ-C6 | -14.09 | . | 228.83 | -11.03 | 8.23 |
| S3HQ-C1 | -19.66 | 7.60 | 568.73 | -3.03 | 1.09 |
| S3HQ-C2 | -19.77 | 5.04 | 320.90 | -5.96 | 6.84 |
| S3HQ-C3 | -19.63 | 8.12 | 393.02 | -10.38 | 6.87 |
| S3HQ-C4 | -19.58 | 6.42 | 365.76 | -0.09 | 1.89 |
| S3HQ-C5 | -20.15 | 2.95 | 211.32 | -6.50 | 8.37 |
| S3HQ-C6 | -19.81 | 5.96 | 234.71 | -9.93 | 11.35 |

**Table S7**. *Posterior standard deviations of model parameters in each simulation scenario with binary data and the temporal correlation parameter $\phi$ modeled. S2 indicates lower, stable concentration, and S3 indicates lower, increasing concentration (S1 not considered). "H" indicates the scenario with higher temporal correlation ($d_0 = 150$; scenarios with $d_0 = 10$ and $\phi$ modeled were not considered). Cluster design scenarios are C1-6, described in Table 3. Relative bias for $\beta_1$ is not reported because the true value was 0.*

| Scenario | $\beta_0$ | $\beta_1$ | $\phi$ | $\sigma^{samp}$ | $\sigma^{time}$ |
|---|---|---|---|---|---|
| S2HQ-C1 | 0.2592 | 0.0004 | 0.5442 | 0.1597 | 0.2450 |
| S2HQ-C2 | 0.2398 | 0.0003 | 0.4743 | 0.2138 | 0.2579 |
| S2HQ-C3 | 0.2442 | 0.0004 | 0.3557 | 0.2073 | 0.2455 |
| S2HQ-C4 | 0.2663 | 0.0004 | 0.5862 | 0.1640 | 0.2462 |
| S2HQ-C5 | 0.2658 | 0.0004 | 0.4326 | 0.2208 | 0.2778 |
| S2HQ-C6 | 0.2473 | 0.0004 | 0.4612 | 0.2116 | 0.2576 |
| S3HQ-C1 | 0.2336 | 0.0003 | 0.6514 | 0.1770 | 0.2388 |
| S3HQ-C2 | 0.2390 | 0.0004 | 0.5500 | 0.2338 | 0.2789 |
| S3HQ-C3 | 0.2337 | 0.0004 | 0.4606 | 0.2255 | 0.2611 |
| S3HQ-C4 | 0.2430 | 0.0004 | 0.6999 | 0.1793 | 0.2521 |
| S3HQ-C5 | 0.2411 | 0.0004 | 0.5654 | 0.2414 | 0.2787 |
| S3HQ-C6 | 0.2503 | 0.0004 | 0.4803 | 0.2356 | 0.2728 |

**Table S8**. *Coverage (95%) of model parameters in each simulation scenario with binary data and the temporal correlation parameter $\phi$ modeled. S2 indicates lower, stable concentration, and S3 indicates lower, increasing concentration (S1 not considered). "H" indicates the scenario*

*with higher temporal correlation ($d_0 = 150$; scenarios with $d_0 = 10$ and $\phi$ modeled were not considered). Cluster design scenarios are C1-6, described in Table 3. Relative bias for $\beta_1$ is not reported because the true value was 0.*

| Scenario | $\beta_0$ | $\beta_1$ | $\phi$ | $\sigma^{samp}$ | $\sigma^{time}$ |
|---|---|---|---|---|---|
| S2HQ-C1 | 0.26 | 0.90 | 0.94 | 0.94 | 0.96 |
| S2HQ-C2 | 0.29 | 0.92 | 0.96 | 0.91 | 0.95 |
| S2HQ-C3 | 0.33 | 0.95 | 0.98 | 0.93 | 0.97 |
| S2HQ-C4 | 0.37 | 0.92 | 0.95 | 0.94 | 0.97 |
| S2HQ-C5 | 0.34 | 0.96 | 0.95 | 0.93 | 0.94 |
| S2HQ-C6 | 0.27 | 0.91 | 0.93 | 0.87 | 0.95 |
| S3HQ-C1 | 0.20 | 0.86 | 0.85 | 0.88 | 0.94 |
| S3HQ-C2 | 0.27 | 0.91 | 0.90 | 0.95 | 0.98 |
| S3HQ-C3 | 0.31 | 0.85 | 0.96 | 0.94 | 0.94 |
| S3HQ-C4 | 0.27 | 0.84 | 0.89 | 0.92 | 0.93 |
| S3HQ-C5 | 0.29 | 0.91 | 0.91 | 0.95 | 0.96 |
| S3HQ-C6 | 0.34 | 0.92 | 0.96 | 0.94 | 0.95 |

# References

Dorazio, Robert M, and Richard A Erickson. 2018. "Ednaoccupancy: An r Package for Multiscale Occupancy Modelling of Environmental DNA Data." *Molecular Ecology Resources* 18 (2): 368–80.

Furlan, Elise M, Dianne Gleeson, Christopher M Hardy, and Richard P Duncan. 2016. "A Framework for Estimating the Sensitivity of eDNA Surveys." *Molecular Ecology Resources* 16 (3): 641–54.

Link, William A, and John R Sauer. 1998. "Estimating Relative Abundance from Count Data." *Austrian Journal of Statistics* 27 (1&2): 83–97.

Stratton, Christian, Adam J Sepulveda, and Andrew Hoegh. 2020. "Msocc: Fit and Analyse Computationally Efficient Multi-Scale Occupancy Models in r." *Methods in Ecology and Evolution* 11 (9): 1113–20.
