# Supplementary material for: Accounting for temporal variation and correlation in environmental DNA sampling can improve ecological inferences
Source: Ecol Appl. 2026 Aug 4;36(5):e70290. doi: 10.1002/eap.70290 (PMC13438698; doi:10.1002/eap.70290)
Supplement: Supplementary file 2 [file EAP-36-e70290-s002.pdf]

Appendix S5

Accounting for temporal variation and correlation in environmental DNA sampling can improve ecological inferences

Ben C. Augustine, Patrick R. Hutchins, Margaret E. Hunter, Christopher M. Merkes, David S. Pilliod, Stephen F. Spear, Caitlin E. Beaver, Scott D. George, Devin N. Jones-Slobodian,| Adam J. Sepulveda

Ecological Applications

Disclaimer: Any use of trade, firm, or product names is for descriptive purposes only and does not imply endorsement by the U.S. Government

Supplementary Simulation Results

Table S1. *Estimated Type I error rate (FPR) for stable trend scenarios (S1 and S2) and statistical power (Power) for increasing trend scenarios (S3) from the equally spaced design scenarios. Trend scenarios are further subdivided by d_0 scenarios (d_0 Level) determining the level of temporal correlation, scenarios where we ignore or model the temporal correlation (Model ϕ), and scenarios varying the number of sampling times. We did not consider scenarios for binary data where we modeled ϕ in equally spaced design scenarios.*

Trend Scenario	FPR/ Power	d_0 Level	Model ϕ	# Sampling Times 8	16	32	64	128	142
S1	FPR	10	No	0.02	0.05	0.04	0.03	0.08	0.05
S1	FPR	150	No	0.06	0.06	0.13	0.20	0.33	0.28
S1	FPR	150	Yes	0.05	0.07	0.12	0.15	0.07	0.15
S2	FPR	10	No	0.03	0.05	0.04	0.04	0.05	0.11
S2	FPR	150	No	0.01	0.05	0.11	0.22	0.25	0.26
S2	FPR	150	Yes	0.02	0.05	0.13	0.15	0.12	0.10
S3	Power	10	No	0.26	0.64	0.91	0.98	1.00	1.00
S3	Power	150	No	0.31	0.70	0.86	0.94	0.98	0.98
S3	Power	150	Yes	0.32	0.67	0.86	0.83	0.84	0.85

Table S2. *Estimated Type I error rate (FPR) for stable trend scenarios (S1 and S2) and statistical power (Power) for increasing trend scenarios (S3) across cluster design scenarios. The cluster design parameters for scenarios enumerated 1-6 can be found in Table 3.*

Trend	FPR/	Cluster Scenario #					
Scenario	Power	1	2	3	4	5	6
S1	FPR	0.08	0.08	0.11	0.05	0.07	0.07
S2	FPR	0.08	0.06	0.03	0.06	0.04	0.05
S3	Power	0.79	0.79	0.85	0.77	0.73	0.82

Table S3. *Relative bias of model parameters in each simulation scenario, equally spaced times, and the temporal correlation parameter ϕ not modeled. S1 indicates higher, stable concentration, S2 indicates lower, stable concentration, and S3 indicates lower, increasing concentration. "L" indicates the scenario with lower temporal correlation ($d_0 = 10$) and "H" indicates the scenario with higher temporal correlation ($d_0 = 150$). Sampling time (design) scenarios are 8, 16, 32, 64, 128, and 142. Scenarios with 142 sampling times did not estimate the sampling standard deviation and the temporal standard deviation estimate is compared to the standard deviation corresponding to the pooled sampling and temporal variance. Relative bias for β_1 is not reported because the true value was 0.*

Scenario	β_0	β_1	σ^y	p^{amp}	σ^{samp}	σ^{time}
S1LQ-8	1.81	.	0.17	1.95	-1.01	-7.99
S1LQ-16	0.92	.	-0.46	0.97	-1.36	-6.00
S1LQ-32	0.59	.	-0.36	1.70	-0.31	-2.79
S1LQ-64	0.25	.	0.68	1.99	-0.94	-2.16
S1LQ-128	0.15	.	-0.44	1.88	8.28	-5.45
S1LQ-142	0.17	.	-1.17	0.05	.	-0.27
S2LQ-8	1.17	.	-0.44	2.78	1.51	0.03
S2LQ-16	0.43	.	-0.59	1.64	1.65	0.22
S2LQ-32	0.18	.	-0.67	1.40	-0.05	-1.32
S2LQ-64	-0.16	.	0.48	2.59	2.50	1.51
S2LQ-128	-0.64	.	-0.37	2.53	4.11	2.38
S2LQ-142	-0.69	.	-0.08	1.76	.	1.00
S3LQ-8	1.58	1.38	-0.09	1.07	1.52	2.79
S3LQ-16	0.22	-0.39	-1.00	3.29	1.83	1.27
S3LQ-32	0.06	-1.66	-1.61	1.88	1.20	1.57
S3LQ-64	-0.53	-2.19	1.67	2.56	0.26	1.98
S3LQ-128	-0.84	-2.86	-0.64	2.55	3.65	1.09
S3LQ-142	-0.67	1.40	1.12	3.10	.	1.41
S1HQ-8	0.98	.	-0.72	0.70	-0.77	-1.00
S1HQ-16	0.39	.	-0.54	0.68	-0.09	0.94
S1HQ-32	0.17	.	0.71	0.28	0.14	-3.88

S1HQ-64	0.08	.	-0.45	1.67	-0.88	-3.63
S1HQ-128	-0.20	.	0.16	0.84	2.45	-5.80
S1HQ-142	-0.11	.	0.43	2.88	.	-3.09
S2HQ-8	2.37	.	-0.22	1.12	-0.15	1.88
S2HQ-16	0.66	.	-0.26	3.34	1.84	-1.98
S2HQ-32	0.35	.	0.53	0.70	-1.06	-2.74
S2HQ-64	-0.14	.	-0.22	1.73	1.86	-5.30
S2HQ-128	-0.50	.	-0.23	2.68	1.80	-3.63
S2HQ-142	0.00	.	0.63	1.93	.	-0.71
S3HQ-8	0.98	5.55	-0.23	2.19	1.79	-4.20
S3HQ-16	0.05	1.17	-0.24	2.49	0.70	-2.67
S3HQ-32	-0.90	1.43	0.97	3.19	2.53	-2.97
S3HQ-64	-1.06	2.91	-0.55	0.56	1.87	-4.65
S3HQ-128	-0.68	1.29	0.27	1.18	1.51	-4.40
S3HQ-142	-1.74	-1.37	0.12	1.69	.	-0.34

Table S4. *Posterior standard deviations of model parameters in each simulation scenario, equally spaced sampling times, and the temporal correlation parameter ϕ not modeled. S1 indicates higher, stable concentration, S2 indicates lower, stable concentration, and S3 indicates lower, increasing concentration. "L" indicates the scenario with lower temporal correlation ($d_0 = 10$) and "H" indicates the scenario with higher temporal correlation ($d_0 = 150$). Sampling time (design) scenarios are 8, 16, 32, 64, 128, and 142. Scenarios with 142 sampling times did not estimate the sampling standard deviation and the temporal standard deviation estimate is compared to the standard deviation corresponding to the pooled sampling and temporal variance. Relative bias for β_1 is not reported because the true value was 0.*

Scenario	β_0	β_1	σ^y	p^{amp}	σ^{samp}	σ^{time}
S1LQ-8	0.4086	0.0006	0.0151	0.0432	0.0517	0.4304
S1LQ-16	0.2382	0.0004	0.0150	0.0422	0.0535	0.2096
S1LQ-32	0.1693	0.0003	0.0150	0.0426	0.0576	0.1435
S2LQ-64	0.1267	0.0002	0.0151	0.0426	0.0691	0.1177
S2LQ-128	0.1022	0.0002	0.0150	0.0421	0.1546	0.2217
S2LQ-142	0.0983	0.0002	0.0150	0.0413	.	0.0751
S3LQ-8	0.4828	0.0007	0.0205	0.0413	0.0830	0.4888
S3LQ-16	0.2669	0.0004	0.0205	0.0406	0.0855	0.2424
S3LQ-32	0.1883	0.0003	0.0203	0.0401	0.0887	0.1676
S1LQ-64	0.1535	0.0002	0.0206	0.0407	0.1073	0.1558
S1LQ-128	0.1312	0.0002	0.0204	0.0408	0.2099	0.2554
S1LQ-142	0.1270	0.0002	0.0206	0.0407	.	0.1069
S2LQ-8	0.5127	0.0007	0.0219	0.0472	0.0941	0.5095
S2LQ-16	0.2784	0.0004	0.0215	0.0460	0.0935	0.2569
S2LQ-32	0.2035	0.0003	0.0211	0.0450	0.0983	0.1824
S3LQ-64	0.1651	0.0002	0.0221	0.0452	0.1158	0.1654

S3LQ-128	0.1440	0.0002	0.0213	0.0449	0.2189	0.2633
S3LQ-142	0.1401	0.0002	0.0216	0.0452	.	0.1147
S1HQ-8	0.4360	0.0007	0.0151	0.0415	0.0524	0.4521
S1HQ-16	0.2542	0.0004	0.0150	0.0413	0.0545	0.2240
S1HQ-32	0.1674	0.0003	0.0151	0.0413	0.0582	0.1425
S1HQ-64	0.1256	0.0002	0.0151	0.0424	0.0694	0.1186
S1HQ-128	0.1005	0.0002	0.0151	0.0415	0.1485	0.2057
S1HQ-142	0.0955	0.0002	0.0151	0.0426	.	0.0729
S2HQ-8	0.4912	0.0007	0.0206	0.0401	0.0815	0.4904
S2HQ-16	0.2607	0.0004	0.0204	0.0410	0.0837	0.2353
S2HQ-32	0.1870	0.0003	0.0208	0.0399	0.0889	0.1660
S2HQ-64	0.1478	0.0002	0.0204	0.0406	0.1078	0.1546
S2HQ-128	0.1281	0.0002	0.0205	0.0411	0.2035	0.2498
S2HQ-142	0.1248	0.0002	0.0207	0.0407	.	0.1043
S3HQ-8	0.4757	0.0007	0.0219	0.0474	0.0942	0.4827
S3HQ-16	0.2700	0.0004	0.0218	0.0465	0.0941	0.2492
S3HQ-32	0.1991	0.0003	0.0216	0.0458	0.0999	0.1813
S3HQ-64	0.1615	0.0002	0.0213	0.0448	0.1170	0.1678
S3HQ-128	0.1409	0.0002	0.0216	0.0448	0.2185	0.2633
S3HQ-142	0.1409	0.0002	0.0215	0.0455	.	0.1142

Table S5. *Coverage (95%) of model parameters in each simulation scenario, equally spaced sampling times, and the temporal correlation parameter ϕ not modeled. S1 indicates higher, stable concentration, S2 indicates lower, stable concentration, and S3 indicates lower, increasing concentration. "L" indicates the scenario with lower temporal correlation ($d_0 = 10$) and "H" indicates the scenario with higher temporal correlation ($d_0 = 150$). Sampling time (design) scenarios are 8, 16, 32, 64, 128, and 142. Scenarios with 142 sampling times did not estimate the sampling standard deviation and the temporal standard deviation estimate is compared to the standard deviation corresponding to the pooled sampling and temporal variance. Relative bias for β_1 is not reported because the true value was 0.*

Scenario	β_0	β_1	σ^y	p^{amp}	σ^{samp}	σ^{time}
S1LQ-8	0.96	0.98	0.94	0.93	0.90	1.00
S1LQ-16	0.94	0.95	0.96	0.94	0.90	0.98
S1LQ-32	0.93	0.96	0.91	0.95	0.90	0.97
S2LQ-64	0.97	0.97	0.93	0.93	0.92	0.95
S2LQ-128	0.96	0.92	0.93	0.97	0.91	0.93
S2LQ-142	0.95	0.95	0.92	0.98	.	0.94
S3LQ-8	0.97	0.97	0.99	0.92	0.95	0.98
S3LQ-16	0.96	0.95	0.96	0.96	0.93	0.93
S3LQ-32	0.92	0.96	0.98	0.94	0.99	0.96
S1LQ-64	0.97	0.96	0.92	0.97	0.90	0.94
S1LQ-128	0.90	0.95	0.96	0.98	0.92	0.96

S1LQ-142	0.96	0.89	0.96	0.96	.	0.95
S2LQ-8	0.97	0.96	0.95	0.93	0.94	0.95
S2LQ-16	0.99	0.99	0.95	0.92	0.95	0.97
S2LQ-32	0.93	0.94	0.93	0.95	0.97	0.94
S3LQ-64	0.96	0.93	0.91	0.95	0.92	0.96
S3LQ-128	0.91	0.94	0.96	0.95	0.92	0.98
S3LQ-142	0.95	0.90	0.97	0.92	.	0.96
S1HQ-8	0.96	0.94	0.92	0.94	0.97	0.97
S1HQ-16	0.99	0.94	0.98	0.93	0.93	0.96
S1HQ-32	0.94	0.87	0.95	0.94	0.92	0.90
S1HQ-64	0.82	0.80	0.94	0.94	0.93	0.90
S1HQ-128	0.66	0.67	0.97	0.97	0.94	0.87
S1HQ-142	0.66	0.72	0.98	0.95	.	0.89
S2HQ-8	0.98	0.99	0.95	0.98	0.95	0.98
S2HQ-16	0.97	0.94	0.95	0.94	0.92	0.97
S2HQ-32	0.95	0.87	0.95	0.96	0.96	0.95
S2HQ-64	0.83	0.81	0.95	0.93	0.96	0.90
S2HQ-128	0.78	0.74	0.91	0.91	0.97	0.93
S2HQ-142	0.77	0.70	0.94	0.93	.	0.91
S3HQ-8	0.99	0.92	0.95	0.92	0.95	0.96
S3HQ-16	0.94	0.92	0.95	0.93	0.98	0.95
S3HQ-32	0.89	0.89	0.97	0.96	0.95	0.96
S3HQ-64	0.85	0.80	0.98	0.94	0.94	0.91
S3HQ-128	0.82	0.81	0.97	0.93	0.93	0.85
S3HQ-142	0.78	0.71	0.92	0.92	.	0.92

Table S6. *Relative bias of model parameters in each simulation scenario with the temporal correlation parameter ϕ modeled. S1 indicates higher, stable concentration, S2 indicates lower, stable concentration, and S3 indicates lower, increasing concentration. "H" indicates the scenario with higher temporal correlation ($d_0 = 150$; scenarios with $d_0 = 10$ and ϕ modeled were not considered). Sampling time (design) scenarios are 8, 16, 32, 64, 128, and 142 for equally spaced scenarios. Scenarios with 142 sampling times did not estimate the sampling standard deviation and the temporal standard deviation estimate is compared to the standard deviation corresponding to the pooled sampling and temporal variance. Cluster design scenarios are C1-6, described in Table 3. Relative bias for β_1 is not reported because the true value was 0.*

Scenario	β_0	β_1	σ^y	p^{amp}	ϕ	σ^{samp}	σ^{time}
S1HQ-8	1.11	.	-0.61	0.62	7482.69	-0.89	-1.05
S1HQ-16	0.43	.	-0.50	0.49	6177.58	-0.18	0.63
S1HQ-32	0.17	.	0.69	0.17	5500.74	0.15	-4.14
S1HQ-64	0.21	.	-0.51	1.66	1022.31	-0.78	-2.31
S1HQ-128	0.11	.	0.17	0.91	32.21	-1.24	2.03
S1HQ-142	-0.15	.	-1.81	4.58	251.49	.	-0.04

S2HQ-8	2.04	.	-0.11	1.28	7427.04	0.02	1.77
S2HQ-16	0.66	.	-0.15	3.39	7317.41	1.60	-1.91
S2HQ-32	0.38	.	0.56	0.66	6116.47	-0.84	-2.49
S2HQ-64	-0.02	.	0.03	1.97	1317.65	1.48	-3.81
S2HQ-128	-0.12	.	-0.12	2.61	164.01	-2.98	4.66
S2HQ-142	0.09	.	0.83	1.75	215.51	.	2.88
S3HQ-8	0.92	5.92	-0.52	2.17	7272.90	1.87	-4.64
S3HQ-16	-0.08	1.60	-0.26	2.44	7499.26	0.69	-3.01
S3HQ-32	-0.73	1.13	1.04	3.29	5938.81	2.74	-2.93
S3HQ-64	-0.82	4.05	-0.63	0.88	2007.26	1.13	-3.28
S3HQ-128	0.03	1.09	0.13	1.28	502.13	-8.75	3.65
S3HQ-142	-1.38	-1.61	0.30	1.77	395.65	.	0.87
S1HQ-C1	0.51	.	-0.21	2.07	73.61	-1.95	-1.85
S1HQ-C2	0.29	.	-0.83	0.91	88.67	-3.83	-0.20
S1HQ-C3	0.60	.	-0.28	1.58	100.52	-8.10	2.13
S1HQ-C4	0.45	.	-0.003	0.98	69.90	-2.33	-3.25
S1HQ-C5	0.21	.	-0.10	0.43	63.13	-4.72	2.15
S1HQ-C6	0.40	.	-0.69	2.08	51.83	-3.77	1.58
S2HQ-C1	-0.30	.	-0.66	3.10	189.93	-4.95	0.23
S2HQ-C2	-0.34	.	-0.39	1.84	378.67	-3.53	3.30
S2HQ-C3	0.11	.	1.00	1.46	139.84	-6.79	6.02
S2HQ-C4	0.07	.	-0.43	2.60	231.08	-1.74	1.00
S2HQ-C5	-0.38	.	-0.44	3.02	126.74	-3.40	7.56
S2HQ-C6	-0.02	.	-2.31	3.32	164.98	-9.07	7.63
S3HQ-C1	0.20	5.97	-0.46	3.24	282.99	-3.56	2.55
S3HQ-C2	0.45	4.30	0.39	1.83	323.12	-0.47	4.36
S3HQ-C3	0.26	8.52	1.16	2.86	214.22	-7.29	6.96
S3HQ-C4	0.74	6.78	0.01	1.71	233.06	0.78	0.48
S3HQ-C5	0.45	1.96	2.18	1.54	127.64	-5.85	5.12
S3HQ-C6	0.43	5.84	0.45	2.38	161.75	-8.23	8.25

Table S7. *Posterior standard deviations of model parameters in each simulation scenario with the temporal correlation parameter ϕ modeled. S1 indicates higher, stable concentration, S2 indicates lower, stable concentration, and S3 indicates lower, increasing concentration. "H" indicates the scenario with higher temporal correlation ($d_0 = 150$; scenarios with $d_0 = 10$ and ϕ modeled were not considered). Sampling time (design) scenarios are 8, 16, 32, 64, 128, and 142 for equally spaced scenarios. Scenarios with 142 sampling times did not estimate the sampling standard deviation and the temporal standard deviation estimate is compared to the standard deviation corresponding to the pooled sampling and temporal variance. Cluster design scenarios are C1-6, described in Table 3. Relative bias for β_1 is not reported because the true value was 0.*

Scenario	β_0	β_1	σ^y	p^{amp}	ϕ	σ^{samp}	σ^{time}
S1HQ-8	0.4239	0.0007	0.0152	0.0416	0.8666	0.0524	0.4423
S1HQ-16	0.2557	0.0004	0.0150	0.0414	0.8664	0.0545	0.2232
S1HQ-32	0.1838	0.0003	0.0151	0.0413	0.8769	0.0582	0.1525
S1HQ-64	0.2190	0.0003	0.0151	0.0424	0.7009	0.0686	0.1758
S1HQ-128	0.2605	0.0004	0.0151	0.0416	0.2482	0.1060	0.2149
S1HQ-142	0.1477	0.0002	0.0146	0.0432	0.2753	.	0.0957
S2HQ-8	0.4624	0.0007	0.0206	0.0401	0.8658	0.0816	0.4765
S2HQ-16	0.2618	0.0004	0.0204	0.0411	0.8671	0.0839	0.2361
S2HQ-32	0.2160	0.0003	0.0208	0.0400	0.8762	0.0890	0.1812
S2HQ-64	0.2220	0.0003	0.0204	0.0406	0.7959	0.1063	0.2003
S2HQ-128	0.2600	0.0003	0.0204	0.0411	0.4559	0.1682	0.2478
S2HQ-142	0.5240	0.0005	0.0233	0.0414	0.2653	.	0.2198
S3HQ-8	0.4428	0.0007	0.0219	0.0475	0.8665	0.0942	0.4696
S3HQ-16	0.2743	0.0004	0.0217	0.0465	0.8580	0.0942	0.2489
S3HQ-32	0.2063	0.0003	0.0216	0.0458	0.8645	0.0997	0.1848
S3HQ-64	0.2164	0.0003	0.0213	0.0448	0.8251	0.1152	0.2023
S3HQ-128	0.2391	0.0003	0.0216	0.0447	0.5330	0.1840	0.2498
S3HQ-142	0.3083	0.0003	0.0271	0.0462	0.4215	.	0.1861
S1HQ-C1	0.2302	0.0003	0.0150	0.0428	0.2064	0.0679	0.1803
S1HQ-C2	0.2228	0.0003	0.0151	0.0418	0.1899	0.0941	0.1855
S1HQ-C3	0.2214	0.0003	0.0150	0.0429	0.0991	0.0882	0.1760
S1HQ-C4	0.2229	0.0003	0.0150	0.0418	0.3223	0.0704	0.1781
S1HQ-C5	0.2351	0.0003	0.0150	0.0415	0.2235	0.1014	0.1951
S1HQ-C6	0.2245	0.0003	0.0149	0.0426	0.1758	0.0961	0.1876
S2HQ-C1	0.2584	0.0004	0.0203	0.0412	0.4006	0.1058	0.2182
S2HQ-C2	0.2762	0.0004	0.0204	0.0408	0.3431	0.1449	0.2400
S2HQ-C3	0.2681	0.0004	0.0209	0.0406	0.2146	0.1379	0.2267
S2HQ-C4	0.2741	0.0004	0.0203	0.0408	0.4832	0.1076	0.2303
S2HQ-C5	0.2688	0.0004	0.0205	0.0414	0.3333	0.1567	0.2464
S2HQ-C6	0.2615	0.0004	0.0198	0.0408	0.3186	0.1497	0.2315
S3HQ-C1	0.2542	0.0004	0.0212	0.0454	0.5017	0.1144	0.2215
S3HQ-C2	0.2768	0.0004	0.0215	0.0448	0.4293	0.1612	0.2589

S3HQ-C3	0.2530	0.0004	0.0215	0.0450	0.3174	0.1541	0.2326
S3HQ-C4	0.2702	0.0004	0.0215	0.0448	0.5539	0.1192	0.2363
S3HQ-C5	0.2654	0.0004	0.0222	0.0446	0.4231	0.1675	0.2537
S3HQ-C6	0.2530	0.0004	0.0214	0.0448	0.3467	0.1588	0.2346

Table S8. *Coverage (95%) of model parameters in each simulation scenario with the temporal correlation parameter ϕ modeled. S1 indicates higher, stable concentration, S2 indicates lower, stable concentration, and S3 indicates lower, increasing concentration. "H" indicates the scenario with higher temporal correlation ($d_0 = 150$; scenarios with $d_0 = 10$ and ϕ modeled were not considered). Sampling time (design) scenarios are 8, 16, 32, 64, 128, and 142 for equally spaced scenarios. Scenarios with 142 sampling times did not estimate the sampling standard deviation and the temporal standard deviation estimate is compared to the standard deviation corresponding to the pooled sampling and temporal variance. Cluster design scenarios are C1-6, described in Table 3. Relative bias for β_1 is not reported because the true value was 0.*

Scenario	β_0	β_1	σ^y	p^{amp}	ϕ	σ^{samp}	σ^{time}
S1HQ-8	0.97	0.95	0.92	0.93	0.40	0.96	0.97
S1HQ-16	0.99	0.93	0.99	0.94	0.36	0.93	0.96
S1HQ-32	0.94	0.88	0.94	0.95	0.69	0.92	0.90
S1HQ-64	0.90	0.85	0.95	0.94	0.85	0.93	0.91
S1HQ-128	0.93	0.93	0.96	0.97	0.95	0.96	0.97
S1HQ-142	0.89	0.85	0.96	0.90	0.03	.	0.92
S2HQ-8	0.97	0.98	0.95	0.98	0.36	0.94	0.97
S2HQ-16	0.97	0.95	0.94	0.93	0.48	0.94	0.97
S2HQ-32	0.97	0.87	0.96	0.96	0.60	0.96	0.93
S2HQ-64	0.84	0.85	0.95	0.94	0.86	0.95	0.95
S2HQ-128	0.91	0.88	0.92	0.91	0.95	0.96	0.95
S2HQ-142	0.92	0.90	0.95	0.95	0.38	.	0.94
S3HQ-8	0.97	0.93	0.95	0.92	0.31	0.97	0.97
S3HQ-16	0.94	0.91	0.93	0.93	0.36	0.97	0.96
S3HQ-32	0.90	0.89	0.97	0.96	0.59	0.94	0.96
S3HQ-64	0.91	0.82	0.98	0.96	0.79	0.96	0.93
S3HQ-128	0.93	0.92	0.96	0.93	0.92	0.91	0.89
S3HQ-142	0.89	0.91	0.93	0.92	0.52	.	0.96
S1HQ-C1	0.89	0.92	0.95	0.96	0.97	0.96	0.97
S1HQ-C2	0.88	0.92	0.96	0.99	0.98	0.95	0.99
S1HQ-C3	0.88	0.89	0.96	0.93	0.94	0.94	0.98
S1HQ-C4	0.85	0.95	0.94	0.97	0.99	0.93	0.96
S1HQ-C5	0.92	0.93	0.98	0.97	0.94	0.97	0.92
S1HQ-C6	0.90	0.93	0.96	0.95	0.97	0.94	0.96
S2HQ-C1	0.88	0.92	0.89	0.88	0.98	0.93	0.94
S2HQ-C2	0.97	0.94	0.98	0.96	0.95	0.95	0.92
S2HQ-C3	0.92	0.97	0.95	0.91	0.98	0.96	0.95

S2HQ-C4	0.93	0.94	0.95	0.89	0.97	0.95	0.99
S2HQ-C5	0.88	0.96	0.97	0.96	0.94	0.95	0.97
S2HQ-C6	0.91	0.95	0.93	0.93	0.94	0.93	0.95
S3HQ-C1	0.89	0.91	0.93	0.93	0.90	0.97	0.94
S3HQ-C2	0.90	0.91	0.97	0.95	0.94	0.96	0.95
S3HQ-C3	0.91	0.89	0.94	0.92	0.97	0.96	0.97
S3HQ-C4	0.92	0.89	0.97	0.97	0.94	0.98	0.94
S3HQ-C5	0.95	0.91	0.94	0.96	0.93	0.92	0.94
S3HQ-C6	0.95	0.90	0.95	0.94	0.98	0.95	0.92
