# Supplementary material for: Accounting for temporal variation and correlation in environmental DNA sampling can improve ecological inferences
Source: Ecol Appl. 2026 Aug 4;36(5):e70290. doi: 10.1002/eap.70290 (PMC13438698; doi:10.1002/eap.70290)
Supplement: Supplementary file 3 [file EAP-36-e70290-s003.pdf]

Appendix S3

Accounting for temporal variation and correlation in environmental DNA sampling can improve ecological inferences

Ben C. Augustine, Patrick R. Hutchins, Margaret E. Hunter, Christopher M. Merkes, David S. Pilliod, Stephen F. Spear, Caitlin E. Beaver, Scott D. George, Devin N. Jones-Slobodian, Adam J. Sepulveda

Ecological Applications

Disclaimer: Any use of trade, firm, or product names is for descriptive purposes only and does not imply endorsement by the U.S. Government

Expanded Simulation Methods

Trend Scenarios. We considered three trend scenarios, S1: a species with a higher mean concentration (1000 copies/liter) that does not change over time, S2: a species with a lower mean concentration (100 copies/liter) that does not change through time, and S3: a species with a lower starting concentration (25 copies/liter) that increases through time (hourly and daily rate of exponential increase of 0.001 and 0.024, respectively).

Sampling Design Scenarios. We considered two sets of design scenarios, one with equally spaced sampling times and a second with cluster sampling (e.g., Irvine, Gitelman, and Hoeting 2007; Hoeting et al. 2006). For all design scenarios, we considered that a fixed number of samples (142) could be collected over a fixed period of time (2170 hours or roughly 3 months) across different numbers of sampling times, with each number of sampling times providing a different level of temporal replication. We chose 142 samples because this is the sampling capacity of the Monterey Bay Aquarium research Institute FIDO sampler if 1 field blank is collected at the beginning and end of the deployment (Yamahara et al. 2025). For the equally spaced design scenarios, samples were allocated across 8, 16, 32, 64, 128, or 142 equally spaced times starting at time 1. In scenarios with fewer than 142 sampling times, we allocated the samples equally across sampling times with the remaining samples after equal allocation assigned to the selected sampling times at random. For example, with 128 sampling times, each sampling time was allocated 1 sample and the remaining 14 samples were allocated to the 128 sampling times at random.

For cluster designs, we selected equally spaced times for the beginning of each cluster and selected sampling times within each cluster at equal subintervals. We considered six combinations of cluster number, cluster size (number of samples per cluster), and within cluster spacing. For example, in our first cluster design with 42 clusters with two samples each, there are 84 total sampling times, and after allocating 1 sample to these 84 sampling times, there are 58 unassigned samples, which are the temporal replicate samples that we allocate at random to these 84 sampling times. Then, the sampling times of the first sample in each cluster are spaced 48 hours apart and the second sample within a cluster is collected 4 hours after the first. In both equal- and cluster-spaced designs, we expected that the sampling variance would be better estimated with fewer sampling times and thus more temporal replication, but temporal trend, variance, and correlation would be better estimated with more sampling times until the sampling variance is no longer well estimated due to sparse temporal replication.

Temporal Correlation Scenarios. We considered three correlation scenarios in terms of the effective range d_0 and whether or not we modeled the temporal correlation. To model temporal correlation, we used the process model in that considers temporal dependence to fit the models, and to ignore it, we used the process model assuming temporal independence. We specified a lower correlation scenario, C1, with $d_0 = 10$ hours, or equivalently, $\phi = 0.3$, where all equally spaced scenarios had negligible temporal correlation given the minimum sampling time spacing of 15 hours. We did not model the temporal correlation in C1, since it was negligible, and we did not consider cluster designs because we did not need to estimate ϕ. Scenarios C2 and C3 both had higher temporal correlation, with $d_0 = 150$ hours, or equivalently, $\phi = 0.02$. In C2, we accounted for the temporal correlation, and in C3 we ignored it to quantify the magnitude that the Type I error rate is inflated.

Parameter Values and Model Fitting. For each scenario, we simulated 100 data sets and fit the models corresponding to each correlation scenario (whether or not ϕ is modeled) using observation model 1 that does not consider undermeasurement. We simulated data sets using the following parameter values, which were the averages of the point estimates from the empirical data sets (excluding the ID site) from the undermeasurement observation models: $\sigma^{samp} = 0.75$, $\sigma^{time} = 0.85$, $p^{amp} = 0.52$, $\sigma^{y} = 0.24$. In addition, we set $\eta = 0$ as the best-case scenario. These values for the nugget and partial sill yield a nugget-to-sill ratio of 0.44, which we expect is large enough for reliable estimation to be challenging based on results from Irvine, Gitelman, and Hoeting (2007).

For scenarios modeling ϕ, we use the process model incorporating temporal correlation, and when not modeling ϕ, we use the process model in that assumes temporal independence. As a caveat, for the sampling scenario with 142 sampling times, we modeled temporal variation only because the sampling standard deviation was not identifiable with no temporal replication. In this scenario, we expected the temporal variance to be estimated as the combination of sampling and temporal variance, that is, $\sqrt{(\sigma^{samp})^2 + (\sigma^{time})^2}$ (the variance of the sum of two normally-distributed random variables is the sum of the variances; Casella and Berger 2024).

For each data set and model, we ran 1 chain of 50,000 iterations, thinned by 4 iterations, and discarded 4,000 pre-thinned iterations. We used posterior modes for point estimates and 95%

HPD intervals for interval estimates. We computed Type I error rates in ecological scenarios S1 and S2 defined as the proportion of data sets where the 95% HPD interval for β_1 did not cover 0, and we computed statistical power in ecological scenario S3 defined as the proportion of data sets where the 95% HPD lower bound of β_1 was greater than 0. Although we do not necessarily advocate for binary decision rules, we argue that statistical power correlates with other measures used to infer evidence for covariate effects, and inflated Type I error rates when using generally uninformative priors indicate posterior variances are being underestimated (e.g., when ignoring temporal correlation). We also use less than nominal 95% coverage to indicate that posterior variances are being underestimated.

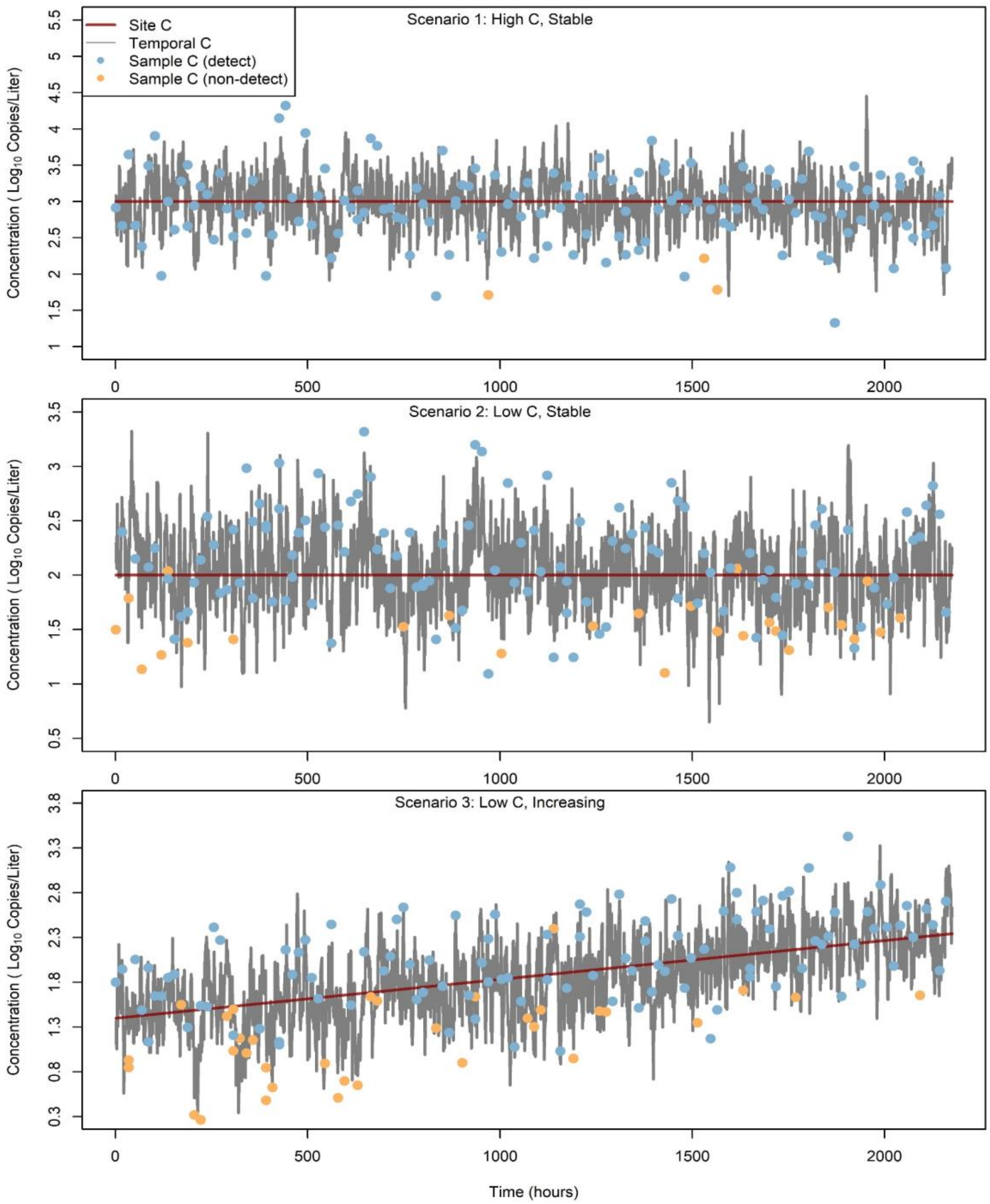

Figure S1. *Plots of a randomly chosen simulated data set from trend scenarios S1-3 with 128 sampling times and lower temporal correlation ($d_0 = 10$), depicting the expected site concentration (Site C), the temporal concentrations at sampled times (Temporal C), and the sample concentrations (Sample C) on the $\log_{10}$ scale. Samples are distinguished between detected samples (at least 1 detected technical replicate) in blue and undetected samples (0 detected technical replicates) in gold, with samples having higher concentrations being more likely to be detected.*

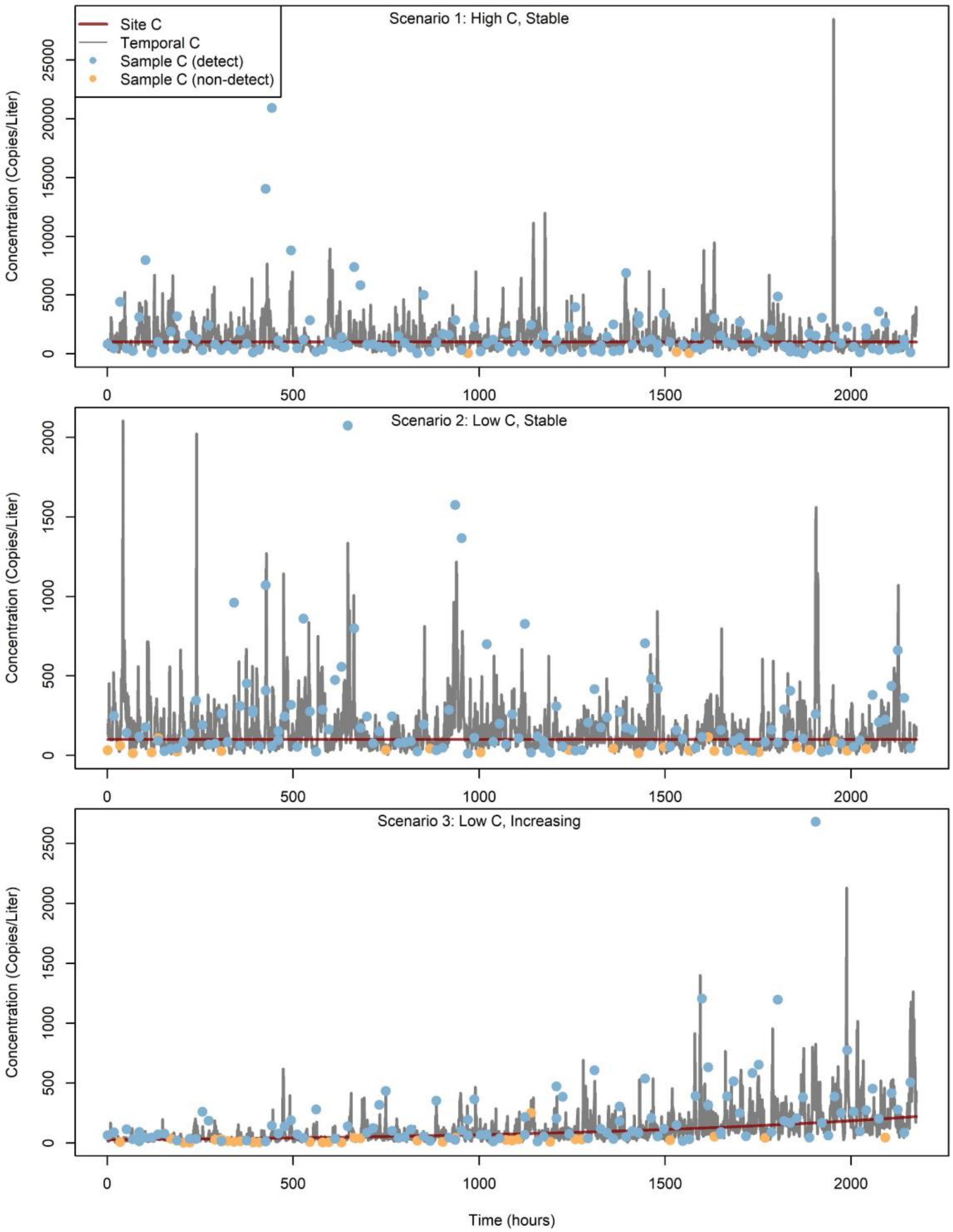

Figure S2. *Reproduction of Figure 1 with concentration plotted on the absolute instead of* log_{10} *scale.*

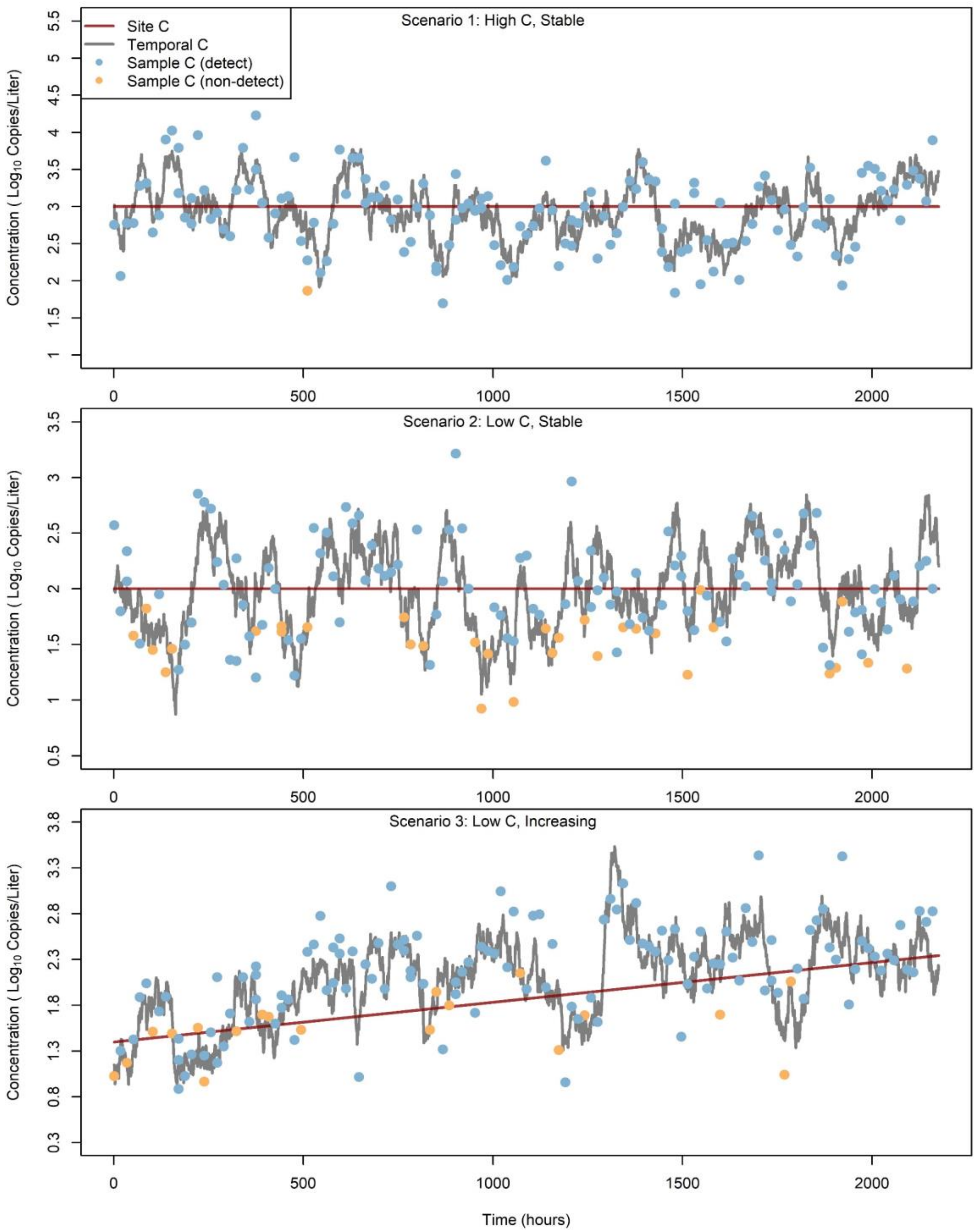

Figure S3. *Plots of a randomly chosen simulated data set from trend scenarios S1-3 with 128 sampling times and higher temporal correlation ($d_0 = 150$), depicting the expected site concentration (Site C), the temporal concentrations at sampled times (Temporal C), and the sample concentrations (Sample C) on the $\log_{10}$ scale. Samples are distinguished between detected samples (at least 1 detected technical replicate) in blue and undetected samples (0 detected technical replicates) in gold, with samples having higher concentrations being more likely to be detected.*

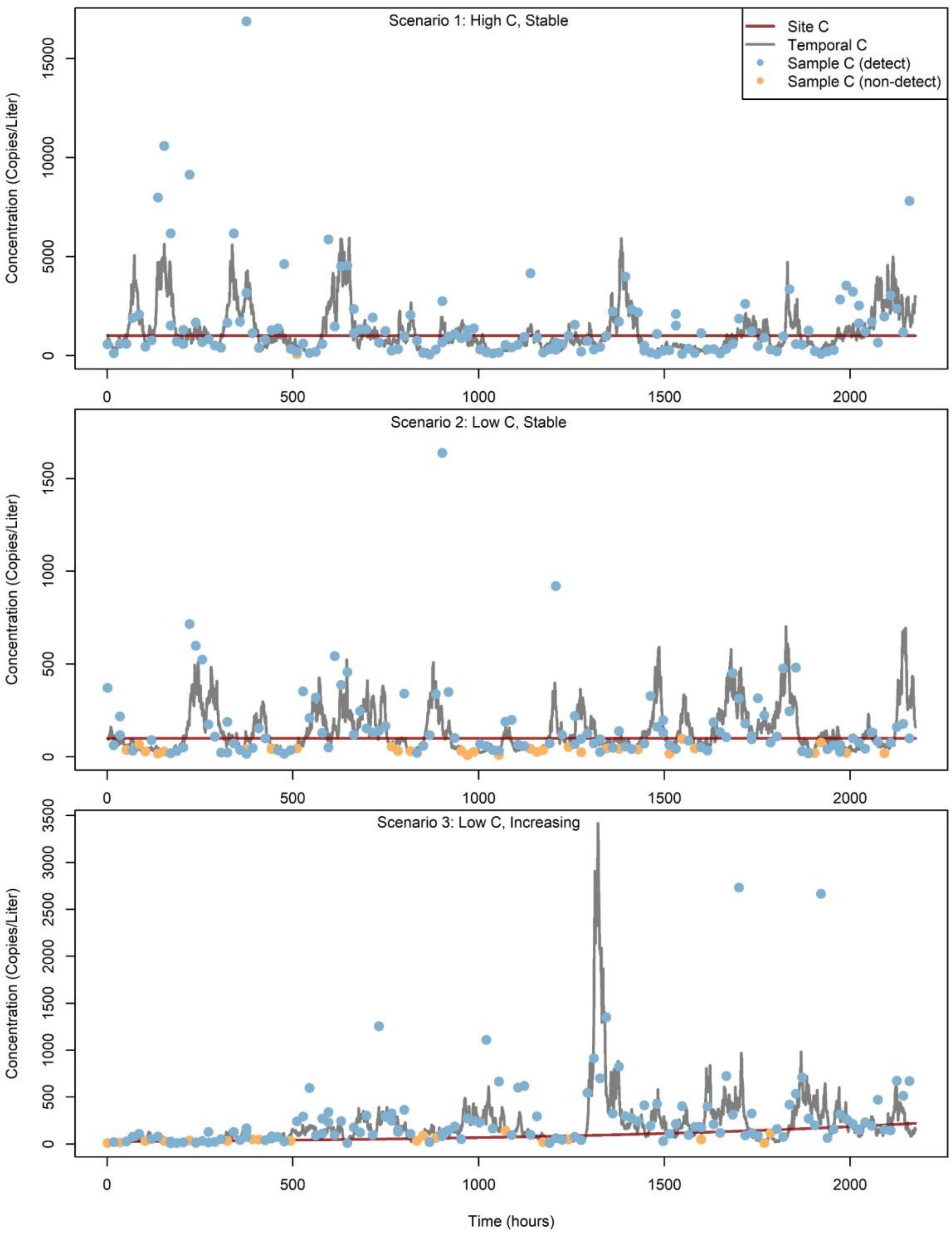

Figure S4. *Reproduction of Figure 3 with concentration plotted on the absolute instead of* log_{10} *scale.*

References

Casella, George, and Roger Berger. 2024. *Statistical Inference*. CRC Press.

Hoeting, Jennifer A, Richard A Davis, Andrew A Merton, and Sandra E Thompson. 2006. "Model Selection for Geostatistical Models." *Ecological Applications* 16 (1): 87–98.

Irvine, Kathryn M, Alix I Gitelman, and Jennifer A Hoeting. 2007. "Spatial Designs and Properties of Spatial Correlation: Effects on Covariance Estimation." *Journal of Agricultural, Biological, and Environmental Statistics* 12: 450–69.

Yamahara, K.M., Allan, E.A., Robidart, J., Wilson, W.H., Birch, J.M., Craw, P., Edson, E., Engstrom, I.B., Fukuba, T., Govindarajan, A.F. and Martins, A.M., 2025. A state-of-the-art review of aquatic eDNA sampling technologies and instrumentation: Advancements, challenges, and future prospects. *Environmental DNA*, *7*(4), p.e70170.
