# Supplementary material for: Accounting for temporal variation and correlation in environmental DNA sampling can improve ecological inferences
Source: Ecol Appl. 2026 Aug 4;36(5):e70290. doi: 10.1002/eap.70290 (PMC13438698; doi:10.1002/eap.70290)
Supplement: Supplementary file 4 [file EAP-36-e70290-s004.pdf]

Appendix S2

Accounting for temporal variation and correlation in environmental DNA sampling can improve ecological inferences

Ben C. Augustine, Patrick R. Hutchins, Margaret E. Hunter, Christopher M. Merkes, David S. Pilliod, Stephen F. Spear, Caitlin E. Beaver, Scott D. George, Devin N. Jones-Slobodian, Adam J. Sepulveda

Ecological Applications

Disclaimer: Any use of trade, firm, or product names is for descriptive purposes only and does not imply endorsement by the U.S. Government

Supplementary Statistical Methods

Nondetection-induced Bias

Before describing our model, we will describe the challenges of estimating unbiased eDNA concentrations in the presence of nondetections that we want to accommodate with our modeling approach. Nondetections do not necessarily imply the absence of eDNA; rather, eDNA may be present but fail to amplify in quantitative or digital droplet PCR (qPCR or ddPCR) technical replicates (hereafter replicates) due to stochastic amplification failure (Furlan et al. 2016) or a minimum possible measurement (e.g., maximum cycle number or limit of blank). The consequences of nondetections for inferred eDNA concentrations depend critically on how detection and quantification are modeled. If nondetections are treated as zero concentrations within a single observation model, eDNA concentration will be underestimated because true but undetected copies are ignored (McCall et al. 2014; Sherina et al. 2020). One approach that has been used to correct for amplification failure and/or lower censoring is multiple imputation (McCall et al. 2014; Sherina et al. 2020); however, this approach assumes that there are always copies allocated to every replicate, which may not be true with very low sample concentrations when we may commonly allocate 0 copies to replicates.

Models that condition measurement on detection can accommodate replicates with no allocated copies (e.g., Shelton et al. 2019, Bylemans et al. 2025) but introduce a new bias in the conditioned measurement model. Specifically, imperfect detection induces selection bias in the measurement model because replicates with lower copy numbers are less likely to be observed and measured. Conditioning measurement on detection implies that at least one copy was present, increasing the expected value of measured replicates relative to the unconditional mean. Failure to account for this selection process results in positive bias in estimated eDNA concentrations (See Figure S1). To our knowledge, the only model that accounts for these selection processes is that of Augustine et al. (2024) that models the selection processes themselves—copy allocation to replicates, amplification as a function of replicate copy number, and lower measurement censoring. Therefore, we adopt this general framework.

Data Description

We consider a survey at a single site where we collect multiple eDNA samples at times t_i for $i = 1, \dots, I$, with time measured in hours. We further consider that $j = 1, \dots, J_i$ samples are collected at time t_i where $J_i > 1$ implies that there is temporal replication at time t_i. Sample j collected at time t_i is subjected to $K_{i,j}$ replicates. Each replicate produces detection data, and the detected replicates produce quantitative measurements of the number of eDNA copies they contain. We define $y_{i,j,k}^{det}$ as the detection outcome, taking the value 1 if eDNA was detected and 0 otherwise, and we define $y_{i,j,k}^{q}$ as the measured eDNA copy number, taking a continuous positive value if eDNA is detected and 0 otherwise.

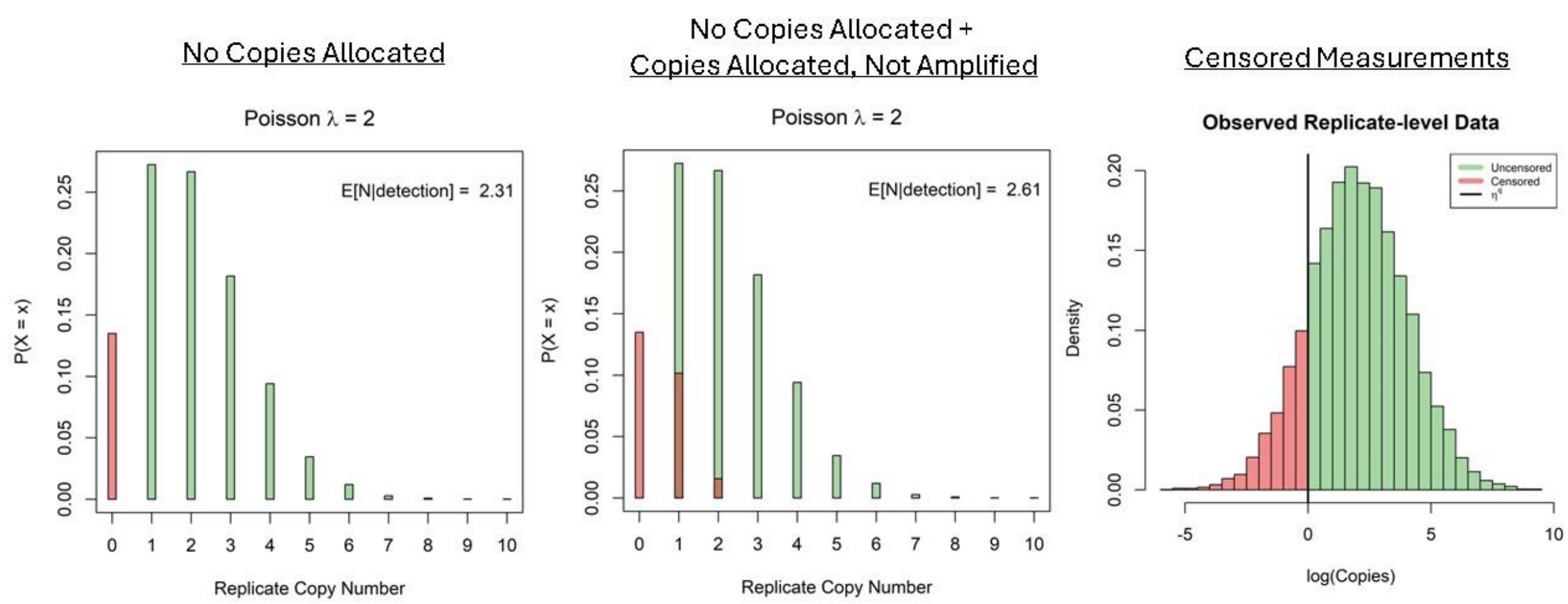

Figure S1. *Selection bias induced by nondetection. Consider a water sample where the mean copy number in each replicates is 2. If we condition on detection in a scenario where we consider that if 0 copies are allocated to a replicate, they cannot be measured, the expected measured concentration would be 2.31, an overestimate. If we further consider that not all allocated copies are amplified, the expected measured concentration would be 2.61 (given*

parameters used for simulation). Finally, if the smallest measurements are then censored, say with a limit of blank, even more positive bias is introduced.

Model Description

Temporal Mean Structure. We assume that our data were generated by a hierarchical model (Wikle 2003; Royle and Dorazio 2008) with an ecological process model describing 1) the concentration of eDNA at the site as a function of time and 2) the variability in the concentration of collected samples that arises from sampling imperfectly mixed water. Then, an observation process, conditioned on the ecological process, describes how the eDNA in collected samples is detected and quantified via PCR. This model is an example of a temporal hierarchical model (Berliner 1996), or equivalently, a spatial hierarchical model (Arab, Hooten, and Wikle 2008) in one dimension. We begin with a model for the mean trend at one site on the scale of (natural) log concentration where concentration is the number of eDNA copies per liter:

$$\log(\boldsymbol{\mu}^{time}) = \mathbf{X}\boldsymbol{\beta}$$

Here, $\boldsymbol{\mu}^{time}$ is a vector of length I, containing the expected site concentrations at all sampling times, $X_i = (1, t_i)$, and $\boldsymbol{\beta} = (\beta_0, \beta_1)$, with β_0 being the log concentration at time 0, and β_1 being the rate of increase in log concentration per hour. Note, this is a continuous time exponential growth model (Gotelli 1998) for expected concentration:

$$\mu_i^{time} = \exp(\beta_0)\exp(\beta_1 t_i)$$

with $\exp(\beta_0)$ being the starting concentration and β_1 being the exponential rate of growth in concentration per hour.

Temporal Variation and Correlation. Next, we model temporal variation and correlation following:

$$\log(\mathbf{C}^{time}) \sim \text{MVN}(\log(\boldsymbol{\mu}^{time}), \boldsymbol{\Sigma})$$

where MVN is the multivariate normal distribution, $\mathbf{C}^{time}$ is a vector of length I containing the realized site concentrations at all sampling times, $\Sigma_{i,j} = (\sigma^{time})^2$ for $i = j$, $\Sigma_{i,j} = \exp(-\phi l_{i,j})(\sigma^{time})^2$ for $i \neq j$, and $l_{i,j}$ is the time difference between times t_i and t_j. ${C^{time}}_i$ can be interpreted as the site-level mean eDNA concentration that would be obtained from a perfectly mixed water sample collected at time t_i.

Sampling Process. We assume that sampling variation follows a normal distribution on the log scale centered around the realized site concentration at time t_i on the log scale:

$$\log(C_{i,j}^{samp}) \sim \text{Normal}(\log(C_i^{time}), \sigma^{samp})$$

where σ^{samp} is the sampling standard deviation describing the variation in the log concentration of water samples collected at the same time (or a very short window of time in which the concentration does not change). The sampling variance reflects variability among discrete water samples and is distinct from laboratory measurement error, which is modeled in the observation process below. Note that this model constitutes a hierarchical specification of an exponential

covariance function, where $(\sigma^{time})^2$ is the partial sill, $(\sigma^{samp})^2$ is the nugget, $(\sigma^{time})^2 + (\sigma^{samp})^2$ is the sill, ϕ is the decay parameter, $d = 1/\phi$ is the range, and $d_0 \approx 3/\phi$ is the effective range, beyond which the temporal or spatial correlation is negligible (<0.05; Banerjee, Carlin, and Gelfand 2015). The effective range, in hour units, will be an important consideration for temporal sampling designs, since temporal correlation can generally be ignored if samples are collected farther apart in time than the effective range (Banerjee, Carlin, and Gelfand 2015). Also of interest is the nugget-to-sill ratio, $\frac{(\sigma^{samp})^2}{(\sigma^{time})^2+(\sigma^{samp})^2}$, because the estimation of the temporal variance and correlation has been found to be more accurate and precise when the sampling variation is smaller relative to the temporal variation (Irvine, Gitelman, and Hoeting 2007).

Finally, for simulation analyses, we consider a simplified ecological process model that ignores temporal correlation in order to evaluate the consequences of misspecifying temporal dependence:

$$\log(C_i^{time}) \sim \text{Normal}(\log(\mu_i^{time}), \sigma^{time}).$$

Pipetting Process. We assume eDNA copies are allocated to replicates following

$$N_{i,j,k} \sim \text{Poisson}(\lambda_{i,j})$$

where the expected number of copies $\lambda_{i,j} = C_{i,j}^{samp} v_{i,j}$. Without subscripts, $v = \frac{v^S v^T}{v^E}$, where v^E is the elution volume in microliters, v^T is the aliquot volume in microliters, and v^S is the volume of the water sample in liters (Lance et al. 2017). These volumes may vary between samples, and a k index can be added if the volume of the aliquot varies between replicates.

PCR amplification and measurement process. We consider two alternative observation models describing the laboratory processes of amplification and measurement, one assuming copy number measurements are unbiased (the null model) and a second assuming that they are stochastically undermeasured (Sepulveda et al. 2020; Augustine et al. 2024). Observation Model 1 serves as a baseline model against which departures from unbiased quantification can be assessed and is expected to perform poorly when stochastic undermeasurement is present. The second observation model was considered to address poor goodness-of-fit of the first observation model for our field data sets.

Baseline amplification and measurement model. We distinguish stochasticity arising from allocation of copies to replicates from stochasticity in DNA amplification and measurement, which are modeled as separate processes. Following Furlan et al. (2016), we define $p_{i,j,k}^N$ as the probability of amplification of replicate k from sample j collected at time t_i conditional on the number of copies allocated to each replicate. We assume each copy in a replicate amplifies independently with probability p^{amp}, implying the probability that at least one copy amplifies is

$$p_{i,j,k}^N = 1 - (1 - p^{amp})^{N_{i,j,k}}$$

and the binary, partially latent amplification states are assumed to arise following

$$[y_{i,j,k}^{amp} | N_{i,j,k}] \sim \text{Bernoulli}(p_{i,j,k}^N).$$

We assume quantitative measurements for amplified replicates, $y_{i,j,k}$, have lognormal measurement error with expected value $\log(N_{i,j,k})$ and standard deviation σ^y:

$$[\log(y_{i,j,k})|N_{i,j,k}, y_{i,j,k}^{amp} = 1] \sim \text{Normal}(\log(N_{i,j,k}), \sigma^y).$$

This model assumes that the measurement error distribution on the quantification cycle (Cq) scale is normal for quantitative PCR (qPCR) data sets (e.g., McCall et al. 2014; Sherina et al. 2020; Espe et al. 2022), and for ddPCR this assumption can be justified in part by the fact that the observations are positive and continuous. By conditioning quantitative measurements on the realized number of allocated copies and on amplification, this formulation accounts for the selection bias induced by nondetection discussed in the Introduction. Note that a literal interpretation of the amplification process above implies that the expected log measurement should be the log of the number of allocated and amplified copies, which is less than or equal to the number of allocated copies. We chose to ignore this implication because most prior literature has assumed that there is no negative bias in the measurements of detected and measured replicates due to imperfect amplification (e.g., McCall et al. 2014; Sherina et al. 2020). We comment further on this in the Discussion.

Finally, we consider that quantitative measurements may be censored from below by η, a minimum possible copy number measurement. Lower censoring could result from only running a limited number of qPCR cycles (e.g., 40; McCall et al. 2014; Sherina et al. 2020; Espe et al. 2022) or removing detections with measurements less than a limit of blank to reduce the potential for false positives. The observed quantitative data after amplification and censoring is $\mathbf{y}^q$, and the probability density of an observed measurement marginalizing over $\mathbf{y}^{amp}$ is:

$$\Pr(y_{i,j,k}^q|y_{i,j,k}) = \begin{cases} 1 - p_{i,j,k}^N + p_{i,j,k}^N \Phi\left(\dfrac{\log(\eta) - \log(N_{i,j,k})}{\sigma^y}\right), & \text{if } y_{i,j,k}^q = 0 \\ p_{i,j,k}^N \times \text{Normal}(\log(N_{i,j,k}), \sigma^y), & \text{if } y_{i,j,k}^q > 0 \end{cases}$$

reflecting an observed zero may arise either from amplification failure or from successful amplification followed by censoring, whereas positive measurements arise only from amplified, uncensored replicates. Note that Φ is the standard normal cumulative distribution function.

Extended model with stochastic undermeasurement. We consider a second observation model using finite mixtures (McLachlan, Lee, and Rathnayake 2019) to address concerns about the under-measurement of replicate copy numbers in qPCR (Sepulveda et al. 2020; Augustine et al. 2024). This model includes latent states corresponding to "unbiased" and "undermeasured" replicates. We assume that under-measurement is a replicate-level stochastic process with $w_{i,j,k}$ being a latent indicator variable for replicate k of sample j collected at time t_i, taking the value 1 if the replicate is under-measured and 0 otherwise. We assume

$$w_{i,j,k} \sim \text{Bernoulli}(p^{mix})$$

where p^{mix} is the probability that a replicate is undermeasured. Next, we assume that the magnitude of undermeasurement depends on the number of copies in a replicate:

$$\text{logit}(\theta_{i,j,k}) = \psi_0 + \psi_1(N_{i,j,k} - 1))$$

where ψ_0 is the thinning rate for undermeasured replicates that contain one copy, and ψ_1, constrained to be positive by its prior distribution (see Appendix S1), determines how quickly the thinning rate increases as the number of copies in a replicate increase. Finally, we introduce $\mathbf{N}^{measure}$, to determine the expected values of the quantitative measurements using the relationship

$$N_{i,j,k}^{measure} = (1 - w_{i,j,k})N_{i,j,k} + w_{i,j,k}N_{i,j,k}\theta_{i,j,k}.$$

We assume that the expected log measurement is $\log(N_{i,j,k})$ and $\log(N_{i,j,k}\theta_{i,j,k})$ for unbiased and under-measured replicates, respectively. Observation Model 1 is recovered as a special case of this formulation when $p^{mix} = 0$ and $\theta_{i,j,k} = 1$ for all replicates.

Empirical Analysis

Priors. Here, we describe our prior distributions. We generally preferred uninformative priors so that parameter estimates were closer to unbiased, coverage closer to nominal, and Type I error rates closer to nominal when models were properly specified. For the temporal correlation parameter ϕ, we restricted the range of the uniform prior to maintain identifiability of both ϕ and σ^{time} using the minimum and maximum lag times in the data set (Banerjee, Carlin, and Gelfand 2015). We also used a uniform prior for σ^{time}, which deviates from general guidance to use an inverse gamma prior (Banerjee, Carlin, and Gelfand 2015), because we found the uniform prior to produce less biased point estimates and interval estimates with closer to nominal coverage. Finally, for the undermeasurement observation model, we used moderately informative priors for ψ_1 due to weak identifiability and/or potential separation (e.g., Clipp et al. 2021).

Table S1. *A list of prior distributions used for the unbiased and undermeasurement observation models (Model 1 and 2, respectively). "U" indicates "Uniform," and "Norm" indicates "Normal"." "T(a,b)" indicates lower truncation at a and upper truncation at b. For ϕ, a and b, corresponding to the maximum and minimum lag time, respectively, varied by data set: MT (a=528,b=3), WI (a=416,b=3), FL (a=580,b=3), and ID (a=408,b=3). Finally, for ψ_1 in the undermeasurement model, we set the the prior standard deviation c to 2.5 for the MT and FL sites and 1.0 for the WI site.*

	Model	
Par	Model 1	Model 2
C_0	U(0,10000)	U(0,10000)
β_1	Norm(0,10)	Norm(0,10)
σ^{time}	U(0,4)	U(0,4)
ϕ	U(3/a,3/b)	U(3/a,3/b)
σ^{samp}	U(0,4)	U(0,4)
logit(p^{amp})	U(0,1)	U(0,1)
σ^{y}	U(0,2)	U(0,2)

$\text{logit}(p^{mix})$	.	Logistic(0,1)
ψ_0	.	Logistic(0,1)
ψ_1	.	T(Norm(0,c),0,∞)

Model Parameterizations. The ability to estimate model parameters with random effects using Markov chain Monte Carlo (MCMC) can depend on the parameterization (centered vs. noncentered) used, particularly with sparse data or weakly informed parameters (Ogle and Barber 2020). For example, a centered parameterization of the model for site concentrations at each sampling time when there is no temporal correlation is:

$$\log(C_i^{time}) \sim \text{Normal}(\log(\mu_i^{time}), \sigma^{time}).$$

Then, the non-centered parameterization is:

$$\log(C_i^{time}) = \log(\mu_i^{time}) + \eta_i \sigma^{time}$$
$$\eta_i \sim \text{Normal}(0,1).$$

For these models with univariate random effects that we considered in the simulation study only, we used the centered parameterization and added a noncentered sampler recently introduced to the Nimble software in Program R (de Valpine et al. 2017), which allows the relevant parameters to be sampled on both scales on each iteration, removing the need to choose one parameterization over the other.

For the multivariate random effects in the models estimating temporal correlation, no analogous sampler exists in Nimble to sample on both scales, and the most appropriate parameterization can depend on the specifics of each data set, or simulation scenario. The centered parameterization is:

$$\log(\mathbf{C}^{time}) \sim \text{MVN}(\log(\boldsymbol{\mu}^{time}), \boldsymbol{\Sigma})$$

and the non-centered parameterization is:

$$\log(\mathbf{C}^{time}) = \log(\boldsymbol{\mu}^{time}) + \mathbf{U}^T\mathbf{z}$$

where $\mathbf{U}$ is a Cholesky factor for a covariance matrix $\boldsymbol{\Sigma}$ and $z_i \sim \text{Normal}(0,1)$ (Gentle 2009). For the empirical data sets, we used noncentered parameterizations for the MT, WI, and ID sites, and a centered parameterization for the FL site. For simulation scenarios where we did not model temporal correlation, we used the centered parameterization and added a noncentered sampler, whereas when we did model temporal correlation, we used the noncentered parameterization.

In addition to the parameterization of the model for temporal variation, the choice between centered and noncentered parameterizations of the sampling variation is also important. For all simulation scenarios, we used a centered parameterization, and for all empirical data sets, we used a noncentered parameterization. The decentered Nimble sampler could not be added to the centered parameterization for the sampling variance because it cannot be used when the distributions of the directly downstream nodes, $\mathbf{N}$, are Poisson. These parameterization choices were informed by preliminary runs to ensure stable and efficient sampling.

MCMC Details. . For each model and data set, we ran 3 chains with 100,000 iterations each, thinned by 5 iterations for parameters and 25 iterations for latent variables. We removed a burn in of 2500 pre-thinned iterations and assessed convergence using the Gelman-Rubin statistic (Brooks and Gelman 1998), ensuring the 95% CI upper bound was less than 1.1 for all parameters.

Variance Partitioning. For analyses of our empirical data sets where we will assume that the expected site concentration does not change over time, we partition the temporal, sampling, and replication variances to compute estimates of the variance proportion coefficients (VPCs), the proportion of variance explained at these three levels, ignoring the variance due to imperfect detection and copy number measurement error. We excluded the variation due to imperfect detection and measurement error because, to our knowledge, the VPCs for this model have not been derived. Using results provided by Leckie et al. (2020) for a 3-level multilevel Poisson model, the marginal expectation is $\mu^{M}_{i,j,k} = \exp(\beta_0 + \log(v_{i,j}) + (\sigma^{time})^2/2 + (\sigma^{samp})^2)/2)$. The level 1 variance is equal to the marginal expectation, the level 2 variance is $(\mu^{M}_{i,j,k})^2\exp((\sigma^{time})^2)\exp((\sigma^{samp})^2 - 1)$, and the level 3 variance is $(\mu^{M}_{i,j,k})^2\exp((\sigma^{time})^2 - 1)$. Then, the VPCs at each level are the level variances divided by the total variance summed across levels. Note, for a given sampling and temporal variance, the VPC at the replicate level increases as the mean concentration decreases.

Posterior Predictive Checks. We compared observation models at the MT, WI, and FL sites using conditional Watanabe Information Criteria (WAIC; Watanabe and Opper 2010), and assessed lack of fit with posterior predictive checks (Gelman et al. 1996) using the observation model deviance as the discrepancy function (King et al. 2009; Conn et al. 2018). We computed Bayesian p-values at both the overall and sample levels. When simulating new data on each MCMC iteration, we first simulated new values for all stochastic parent nodes of the observation model ($\mathbf{N}$ for both models and $\mathbf{w}$ for the undermeasurement observation model). We considered more extreme Bayesian p-values as providing additional evidence of lack of fit (Conn et al. 2018) and used them at the sample level to identify the most extreme data points under each model. As a graphical goodness-of-fit check (Gelman 2003), we plotted the posterior modes and HPD intervals of the expected quantitative measurements versus the observed data. The expected quantitative measurements were $\log(\mathbf{N})$ and $\log(\mathbf{N}^{measure})$ in the unbiased and undermeasurement observation models, respectively. Positive observations (excluding nondetections) in $\log(\mathbf{y}^{time})$ were assumed to be linearly related to these expected measurements, so we interpreted visual deviations from linearity as evidence for lack of fit. We expected more evidence of lack of fit using the unbiased measurement model in the two qPCR data sets (MT and WI sites) compared to the ddPCR data set (FL site).

References

Banerjee, Sudipto, Bradley P Carlin, and Alan E Gelfand. 2015. *Hierarchical Modeling and Analysis for Spatial Data*. Chapman; Hall/CRC.

Brooks, Stephen P, and Andrew Gelman. 1998. "General Methods for Monitoring Convergence of Iterative Simulations." *Journal of Computational and Graphical Statistics* 7 (4): 434–55.

Bylemans, J., T. Everts, R. Brys, and R. P. Duncan. 2025. "From Anarchy to Clarity, Data Pre-Processing and Statistical Choices Influence Quantitative Environmental DNA (eDNA) Analyses." *Methods in Ecology and Evolution* 16, no. 7: 1322–1333

Clipp, Hannah L, Amber L Evans, Brin E Kessinger, Kenneth Kellner, and Christopher T Rota. 2021. "A Penalized Likelihood for Multispecies Occupancy Models Improves Predictions of Species Interactions." *Ecology* 102 (12): e03520.

Conn, Paul B, Devin S Johnson, Perry J Williams, Sharon R Melin, and Mevin B Hooten. 2018. "A Guide to Bayesian Model Checking for Ecologists." *Ecological Monographs* 88 (4): 526–42.

de Valpine, Perry, Daniel Turek, Christopher J Paciorek, Clifford Anderson-Bergman, Duncan Temple Lang, and Rastislav Bodik. 2017. "Programming with Models: Writing Statistical Algorithms for General Model Structures with NIMBLE." *Journal of Computational and Graphical Statistics* 26 (2): 403–13.

Gelman, Andrew, Xiao-Li Meng, and Hal Stern. 1996. "Posterior Predictive Assessment of Model Fitness via Realized Discrepancies." *Statistica Sinica*, 733–60.

Gelman, Andrew. 2003. "A Bayesian Formulation of Exploratory Data Analysis and Goodness-of-Fit Testing." *International Statistical Review* 71 (2): 369–82.

Gotelli, Nicholas J. 1998. *A Primer of Ecology.* Second. Sunderland: Sinauer Associates.

Gentle, James E. 2009. *Computational Statistics*. Vol. 308. Springer.

King, Ruth, Byron Morgan, Olivier Gimenez, and Steve Brooks. 2009. *Bayesian Analysis for Population Ecology*. CRC press.

Leckie, George, William J Browne, Harvey Goldstein, Juan Merlo, and Peter C Austin. 2020. "Partitioning Variation in Multilevel Models for Count Data." *Psychological Methods* 25 (6): 787.

McLachlan, Geoffrey J, Sharon X Lee, and Suren I Rathnayake. 2019. "Finite Mixture Models." *Annual Review of Statistics and Its Application* 6: 355–78.

Ogle, Kiona, and Jarrett J Barber. 2020. "Ensuring Identifiability in Hierarchical Mixed Effects Bayesian Models." *Ecological Applications* 30 (7): e02159.

Royle, J Andrew, and Robert M Dorazio. 2008. *Hierarchical Modeling and Inference in Ecology: The Analysis of Data from Populations, Metapopulations and Communities*. Elsevier.

Sepulveda, Adam J, Patrick R Hutchins, Craig Jackson, Carl Ostberg, Matthew B Laramie, Jon Amberg, Tim Counihan, Andrew Hoegh, and David S Pilliod. 2020. "A Round-Robin Evaluation of the Repeatability and Reproducibility of Environmental DNA Assays for Dreissenid Mussels." *Environmental DNA* 2 (4): 446–59.

Shelton, Andrew Olaf, Ryan P. Kelly, James L. O'Donnell, Linda Park, Piper Schwenke, Correigh Greene, Richard A. Henderson, and Eric M. Beamer. "Environmental DNA provides

quantitative estimates of a threatened salmon species." *Biological Conservation* 237 (2019): 383-391.

Sherina, Valeriia, Helene R McMurray, Winslow Powers, Harmut Land, Tanzy MT Love, and Matthew N McCall. 2020. “Multiple Imputation and Direct Estimation for qPCR Data with Non-Detects.” *BMC Bioinformatics* 21: 1–15.

Watanabe, Sumio, and Manfred Opper. 2010. “Asymptotic Equivalence of Bayes Cross Validation and Widely Applicable Information Criterion in Singular Learning Theory.” *Journal of Machine Learning Research* 11 (12).

Wikle, Christopher K. 2003. “Hierarchical Bayesian Models for Predicting the Spread of Ecological Processes.” *Ecology* 84 (6): 1382–94.
