# Supplementary material for: Accounting for temporal variation and correlation in environmental DNA sampling can improve ecological inferences
Source: Ecol Appl. 2026 Aug 4;36(5):e70290. doi: 10.1002/eap.70290 (PMC13438698; doi:10.1002/eap.70290)
Supplement: Supplementary file 5 [file EAP-36-e70290-s005.pdf]

Appendix S1

Accounting for temporal variation and correlation in environmental DNA sampling can improve ecological inferences

Ben C. Augustine, Patrick R. Hutchins, Margaret E. Hunter, Christopher M. Merkes, David S. Pilliod, Stephen F. Spear, Caitlin E. Beaver, Scott D. George, Devin N. Jones-Slobodian, Adam J. Sepulveda

Ecological Applications

Disclaimer: Any use of trade, firm, or product names is for descriptive purposes only and does not imply endorsement by the U.S. Government

Supplementary Field and Lab Methods

Site #1: Montana (MT)

Field negative control. A 1-L negative field control was collected at the start of each sampling day by pouring 1 L of reverse-osmosis water into a sterile, single-use Whirl-Pak bag (Nasco, Fort Atkinson, Wisconsin, USA), exposing the bag to the field environmental for 10 s, and then closing the bag. Field negative controls were then transported with field samples to the Northern Rocky Mountain Science Center laboratory, where they were promptly filtered within an hour of collection.

DNA Extraction. Within 48 hours of receipt, eDNA sample filters were extracted in one batch with Qiagen DNeasy Blood and Tissue Kits (cat. #69504, Valencia, CA) using Qiagen DNeasy Lyse and Spin Baskets (cat. #19598) with minimal alterations of the manufacturer's protocol. DNA was eluted in 300 µL of Qiagen Buffer AE. One extraction negative control was produced using only extraction kit reagents. The extraction blank was otherwise handled and analyzed identically to samples.

qPCR. Within 48 hours of DNA extraction, the New Zealand mudsnail (NZMS) assay of Goldberg et al. (2013) was used to amplify DNA with a BioRad CFX 96-Touch or BioRad Opus thermal cycler (Hercules, CA). Reactions (five technical replicates per filter sample) took place in BioRad Hard-Shell®optical 96-well plates (cat. #HSP9601) sealed with BioRad

Microseal® optical adhesive film (cat. #MSC1001). The thermal cycle was: 95 °C for 15 minutes then 50 cycles of 94 °C for 15 seconds and 60 °C for 60 seconds. Reactions were multiplexed with an internal positive control (IPC; thermofisher.com cat. #4308323) and included 10 µL of Qiagen Quantitect Master Mix, 2 µL of IPC assay mix, 0.4 µL of IPC DNA, 0.4 µM of each of the forward and reverse primers, 0.25 µM FAM-labeled hydrolysis probe, 3 µL of DNA extract, and sterile water to reach 20 µL reaction volumes. Every 96-well plate run as part of this study had the same duplicated (two replicates) controls: six-point positive control synthetic DNA standard curve ranging from 1.2 to 3e4 target DNA copies, no-template control (NTC), and internally blocked negative control (IBC). FAM and VIC fluorescence values were ROX-normalized prior to baseline correction (including regressing the fluorescence curve such that the slope of baseline fluorescence was equal to zero) and amplification detection (Patrone et al. 2020) using R v4.0.3 statistical software (R Core Team 2021). Successful amplification of DNA was defined as any curve for which a feasible solution with less than 10% transformation error was found for the non-linear optimization transformation presented in Patrone et al. (2020).

Site #2: Wisconsin (WI)

Field negative control. A 1-L ultra-pure water field negative was also collected, randomly associated with one field sampling per day.

DNA Extraction. We extracted DNA from filters using the IBI gMax mini DNA kit (IBI Scientific, Dubuque, IA), following the manufacturer protocol with the exception that we conducted the initial lysis of the filter using the Qiagen Investigator Lyse and Spin basket kit (Qiagen Inc., Germantown, MD). Specifically, we added 380 µl of GST Buffer and 20 µl of Proteinase K from the IBI kit to the filter in the lyse and spin basket. We incubated samples at 60 °C for 30 minutes and then centrifuged at 16,000 x g for two minutes. We then moved the lysate to the next step using the standard IBI protocol.

qPCR. Northern pike eDNA detection was carried out using qPCR using the EluCOI assay published in Olsen et al. (2015). We ran 25 µL qPCR reactions in quadruplicate with final concentrations of: 1x Perfecta ToughMix qPCR master mix (Quantabio), 500 nanomolar (nM) forward and reverse primers, 250 nM hydrolysis probe, and 2 µl of sample. We ran reactions using CFX96 real-time thermocyclers with an initial step of 50 °C for 2 minutes and 95 for 10 minutes, followed by 50 cycles of amplification, with denaturation at 95 °C for 15 sec and annealing at 60 °C for 1 minute. We ran each sample in quadruplicate. We used BioRad CFX Manager Version 3.1 software for visual inspection and to estimate copy numbers based on the standard curve of qPCR data. The standard curve was based on serial dilutions (10000, 1000, 100, and 10 copies/µl) of synthetic target DNA sequence (gBlock) representing the amplified region. We tested for PCR inhibition using the approach described in Lor et al. (2020). Briefly, we added samples in triplicate to a reaction mix that was the same as described above with the exception that 200 copies of gBlock per reaction were spiked into the mix.

Site #3: Florida (FL)

Field negative control. A field negative control was taken each day before the last environmental sample by pouring ultra-pure water from a sterilized 1-liter Nalgene bottle, into an empty pre-sterilized 1-liter Nalgene bottle while at the site.

DNA Extraction. All filters were incubated in 2 mL of modified CTAB buffer (described in Hunter et al. 2019a) and were incubated at 4 ˚C for 4-7 days. After incubation, Proteinase K (200 µl, 20 mg/ml) was added, and samples were incubated at 55 ˚C for 1 hour. eDNA was isolated according to the phenol chloroform isoamyl alcohol (PCI) method described in experiment 2 by Hunter et al. (2019a) but with some modifications as follows. After the addition of Chloroform Isoamyl Alcohol (CI) and centrifugation, the 2 mL aqueous layer was moved to a new 5 mL tube and 2 mL PEG (30% Polyethylene Glycol/2.5 M NaCl) was added to each sample. The addition of the PEG was in response to heavy inhibitor loads found in southern Florida water samples (Hunter et al. 2019a; Hunter et al. 2019b). Samples were vortexed thoroughly and precipitated overnight at 4 ˚C. The next day, eDNA was pelleted by centrifugation at 10,000 x g for 10 minutes. All liquid was then removed via decantation and discarded. The eDNA pellet was washed with 1 mL of cold 70% ethanol, and centrifuged at 10,000 RPM for five minutes, then the liquid was discarded, and this was repeated for two total washes. Following the second wash, eDNA pellets were allowed to air dry until all residual ethanol had evaporated. The eDNA pellet was rehydrated with 100 µL 1x TE (10 mM Tris-HCl, 1 mM EDTA, pH 8.0).

ddPCR. Grass Carp (*Ctenopharyngodon idella*) eDNA detection was tested in a multiplex reaction with an internal positive control (IPC) on the Bio-Rad QX200 Droplet Digital (dd) PCR System (Bio-Rad, Hercules, CA). Samples were tested in reactions of 25 uL reactions with 4 uL of template eDNA, 12.5 uL of ddPCR Supermix for Probes no dUTP (Bio-Rad), 2 uL of each assay component (primers at 980 nM, VIC-labeled probe at 150 nM), and ultra-pure water. An IPC was run concurrently with the YWHAZ Rhesus Monkey PrimePCR assay and template (Bio-Rad) with 1 uL of the assay mix and 0.2 uL of the IPC DNA diluted to a final concentration of 10^{-2} copies/uL and added to the reaction. Plates included 2 replicates each of a positive control using Grass Carp isolated DNA from tissue, a no-template control (NTC), and five PCR replicates per sample. The PCR program started with the denaturing step at 95 ˚C for 10 minutes, then annealing with 40 cycles of 94 ˚C for 30 sec and 60 ˚C for 1 min, with a final incubation at 98 ˚C for 10 minutes and an infinite hold at 4 ˚C.

Site #4: Idaho (ID)

Field negative control. We collected one field blank per day of sampling using 0.5 L of distilled water pumped through a single filter using the same set up streamside as field samples.

DNA Extraction. We extracted DNA from the filters using the standard Goldberg protocol (Goldberg et al. 2011).

qPCR. All samples were tested for the presence and amount of *Anodonta nuttalliana DNA* using the assay described in Rodgers et al. (2020). Each sample included an internal positive control (EXO-IPC; Life Technologies, Corp.) in each well. We ran samples, PCR non-template controls, and standards in triplicate on 384-well plates. We calculated a standard curve with the serial dilutions of gBlock gene fragments for *Anodonta nuttalliana* (10-2 thru 105, 0.01 copy thru 100,000 copies) with each plate on a BioRad CFX384 Touch Real-Time PCR Detection System (Bio-Rad Laboratories, Inc., California, USA).

References

Goldberg, Caren S, David S Pilliod, Robert S Arkle, and Lisette P Waits. 2011. "Molecular Detection of Vertebrates in Stream Water: A Demonstration Using Rocky Mountain Tailed Frogs and Idaho Giant Salamanders." *PloS One* 6 (7): e22746.

Goldberg, Caren S, Adam Sepulveda, Andrew Ray, Jeremy Baumgardt, and Lisette P Waits. 2013. "Environmental DNA as a New Method for Early Detection of New Zealand Mudsnails (Potamopyrgus Antipodarum)." *Freshwater Science* 32 (3): 792–800.

Hunter, Margaret E, Jason A Ferrante, Gaia Meigs-Friend, and Amelia Ulmer. 2019a. "Improving eDNA Yield and Inhibitor Reduction Through Increased Water Volumes and Multi-Filter Isolation Techniques." *Scientific Reports* 9 (1): 5259.

Hunter, Margaret E, Gaia Meigs-Friend, Jason A Ferrante, Brian J Smith, and Kristen M Hart. 2019b. "Efficacy of eDNA as an Early Detection Indicator for Burmese Pythons in the ARM Loxahatchee National Wildlife Refuge in the Greater Everglades Ecosystem." *Ecological Indicators* 102: 617–22.

Lor, Yer, Theresa M Schreier, Diane L Waller, and Christopher M Merkes. 2020. "Using Environmental DNA (eDNA) to Detect the Endangered Spectaclecase Mussel (Margaritifera Monodonta)." *Freshwater Science* 39 (4): 837–47.

Olsen, Jeffrey B, Cara J Lewis, Robert L Massengill, Kristine J Dunker, and John K Wenburg. 2015. "An Evaluation of Target Specificity and Sensitivity of Three qPCR Assays for Detecting Environmental DNA from Northern Pike (Esox Lucius)." *Conservation Genetics Resources* 7 (3): 615–17.

Patrone, Paul N, Anthony J Kearsley, Jacob M Majikes, and J Alexander Liddle. 2020. "Analysis and Uncertainty Quantification of DNA Fluorescence Melt Data: Applications of Affine Transformations." *Analytical Biochemistry* 607: 113773.

R Core Team. 2021. *R: A Language and Environment for Statistical Computing*. Vienna, Austria: R Foundation for Statistical Computing. *https://www.R-project.org/*.

Rodgers, Torrey W, Joseph C Dysthe, Cynthia Tait, Thomas W Franklin, Michael K Schwartz, and Karen E Mock. 2020. "Detection of 4 Imperiled Western North American Freshwater Mussel Species from Environmental DNA with Multiplex qPCR Assays." *Freshwater Science* 39 (4): 762–72.
