# Supplementary material for: Accounting for temporal variation and correlation in environmental DNA sampling can improve ecological inferences
Source: Ecol Appl. 2026 Aug 4;36(5):e70290. doi: 10.1002/eap.70290 (PMC13438698; doi:10.1002/eap.70290)
Supplement: Supplementary file 6 [file EAP-36-e70290-s006.pdf]

Appendix S4

Accounting for temporal variation and correlation in environmental DNA sampling can improve ecological inferences

Ben C. Augustine, Patrick R. Hutchins, Margaret E. Hunter, Christopher M. Merkes, David S. Pilliod, Stephen F. Spear, Caitlin E. Beaver, Scott D. George, Devin N. Jones-Slobodian, Adam J. Sepulveda

Ecological Applications

Disclaimer: Any use of trade, firm, or product names is for descriptive purposes only and does not imply endorsement by the U.S. Government

Expanded Empirical Results

Table S1. *Parameter point and interval estimates for the MT, WI, and FL empirical data sets from the null model. Point estimates are posterior modes except for the variance partition coefficients (VPC), which are posterior means to ensure they add to one. Interval estimates are 95% HPD intervals. The effective range, d_0 is derived from ϕ and is in units of hours.*

	MT			WI			FL		
	Est	LB	UB	Est	LB	UB	Est	LB	UB
σ^y	1.58	1.42	1.79	1.10	0.97	1.24	0.08	0.06	0.10
β_0	5.22	4.75	5.86	4.21	3.60	4.76	6.90	6.27	7.67
p^{amp}	1.00	0.93	1.00	0.80	0.59	1.00	0.36	0.29	0.42
ϕ	0.017	0.006	0.864	0.197	0.093	0.958	0.013	0.005	0.869
d_0	176	3	500	15	3	32	231	3	600
σ^{samp}	0.62	0.14	1.04	0.61	0.22	1.02	0.93	0.74	1.21
σ^{time}	0.73	0.15	1.31	1.01	0.62	1.49	0.88	0.46	1.53
VPC Rep	0.22	0.01	0.46	0.20	0.00	0.42	0.01	0.00	0.02
VPC Samp	0.39	0.00	0.75	0.34	0.00	0.61	0.72	0.53	0.91
VPC Time	0.39	0.00	0.75	0.46	0.17	0.77	0.28	0.08	0.46

Table S2. *Parameter point and interval estimates for the three empirical data sets from the undermeasurement model. Point estimates are posterior modes except for the variance partition coefficients (VPC), which are posterior means to ensure they sum to one. Interval estimates are 95% HPD intervals. The effective range,* d_0 *is derived from* ϕ *and is in units of hours.*

	MT			WI			FL		
	Est	LB	UB	Est	LB	UB	Est	LB	UB
σ^y	0.27	0.20	0.38	0.37	0.30	0.47	0.08	0.06	0.10
β_0	6.10	5.52	6.86	4.48	3.96	4.99	6.89	6.24	7.60
p^{amp}	0.54	0.38	0.76	0.60	0.46	0.78	0.41	0.32	0.51
p^{mix}	1.00	0.97	1.00	0.03	0.01	0.07	1.00	0.66	1.00
ψ_0	-2.82	-3.00	-2.58	-6.46	-7.98	-5.37	-1.37	-3.05	-1.10
ψ_1	0.21	0.12	0.35	0.21	0.06	0.94	3.50	2.50	5.88
ϕ	0.011	0.006	0.811	0.233	0.127	0.982	0.023	0.005	0.875
d_0	273	4	500	13	3	24	130	3	600
σ^{samp}	0.79	0.51	1.16	0.53	0.22	0.84	0.94	0.75	1.20
σ^{time}	0.72	0.21	1.40	0.96	0.64	1.41	0.86	0.47	1.53
VPC Rep	0.06	0.00	0.14	0.20	0.01	0.40	0.01	0.00	0.02
VPC Samp	0.64	0.35	0.94	0.28	0.04	0.53	0.72	0.53	0.90
VPC Time	0.31	0.00	0.58	0.52	0.25	0.79	0.27	0.09	0.45

Table S3. *Parameter point and interval estimates for the ID site empirical data set from the null model. Point estimates are posterior modes except for the variance partition coefficients (VPC), which are posterior means to ensure they add to one. Interval estimates are 95% HPD intervals. The effective range,* d_0 *is derived from* ϕ *and is in units of hours.*

	Est	LB	UB
σ^y	0.51	0.38	0.72
β_0	0.60	-0.23	1.55
p^{amp}	0.36	0.18	0.93
ϕ	0.064	0.007	0.948
d_0	47	3	429
σ^{samp}	0.04	0.00	0.83
σ^{time}	0.06	0.00	0.72
VPC Rep	0.89	0.66	1.00
VPC Samp	0.06	0.00	0.24
VPC Time	0.04	0.00	0.17

Table S4. *Replicate-level copy number observations and Bayesian p-values for samples with sample-level Bayesian p-values from posterior predictive checks that are less than 0.1, for each site and model. Measurements are rounded to 4 digits and p-values are rounded to 2 digits. Nondetects are indicated by "0" and "." indicates that there was no fifth replicate at the WI site. No samples at the MT site had a p-value of less than 0.1 in the undermeasurement (UM) model.*

Site	Model	Rep 1	Rep 2	Rep 3	Rep 4	Rep 5	p-value
MT	Null	0	0.0812	0.0467	0.1000	0.0985	0.03
MT	Null	0.0363	0.1132	0.0597	0.2293	0.1768	0.01
MT	Null	0	0.5483	0.0506	0.0462	0.0570	0.02
MT	Null	0.4099	0.0392	0.4356	1.1240	0.0898	0.04
MT	Null	0.0523	0.0434	0.0459	0.4888	0.0539	0.02
MT	Null	0.0977	0.1009	0.1606	0.1261	0.7383	0.03
MT	Null	0.5112	0.5904	0.0734	0.5887	0.1170	0.09
MT	Null	1.4112	0.0717	1.2589	0.8639	0.0726	0.09
MT	Null	0.0634	0	0.1752	0.0569	0.0488	0.02
MT	Null	0.0374	0	0.0522	0.4178	0.4331	0.07
WI	Null	0.0041	0	0	2.1760	.	0.00
WI	Null	0	1.5225	0.0060	3.1500	.	0.00
WI	Null	0	0.0015	0.7872	0	.	0.00
FL	Null	40.6215	0	9.1486	5.0014	10.4748	0.02
WI	UM	1.1375	0.4625	1.5725	0.5225	.	0.02
FL	UM	40.6215	0	9.1486	5.0014	10.4748	0.01

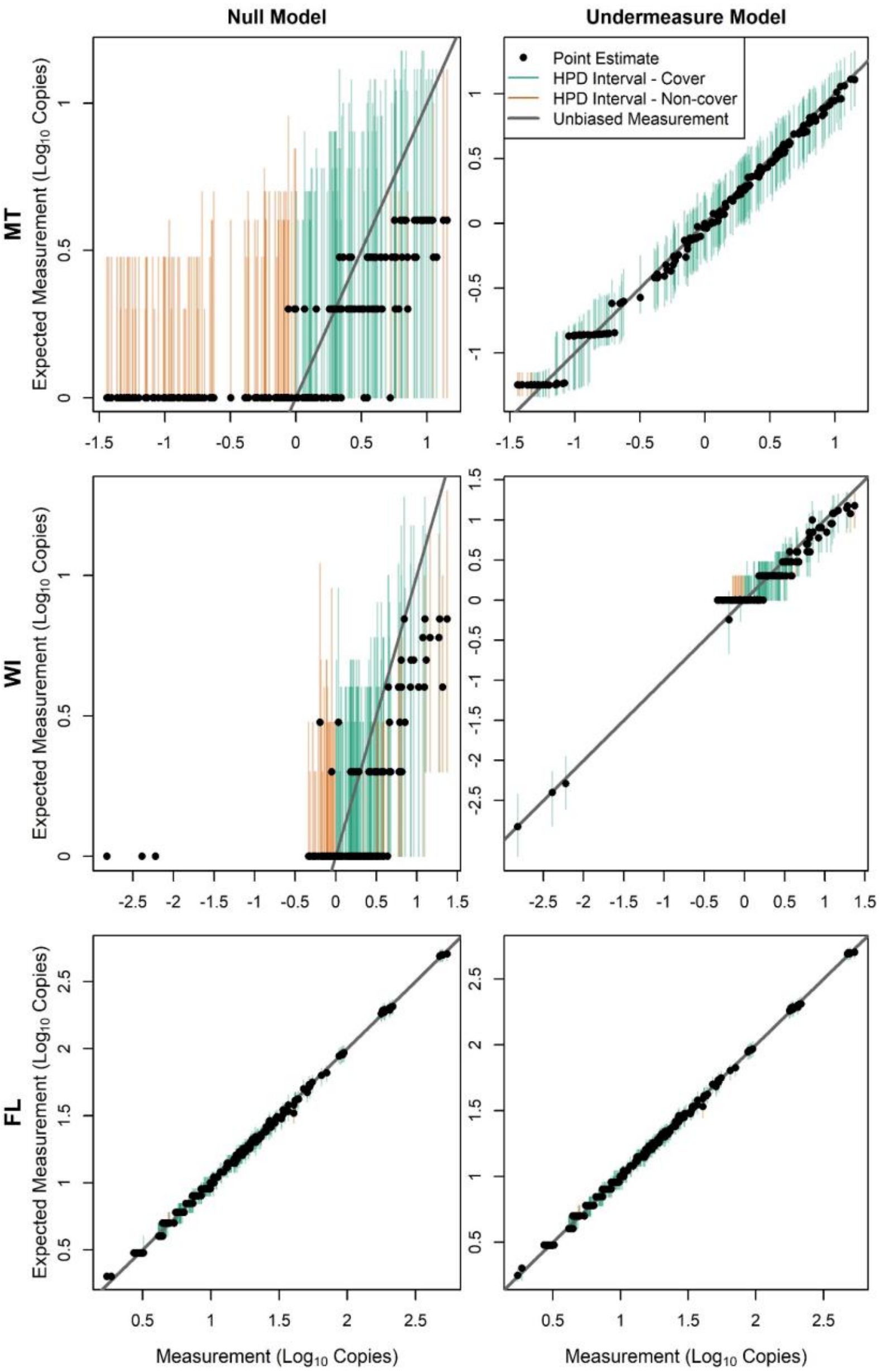

Figure S1. *Graphical goodness-of-fit plots showing the relationship between the measured log-copy numbers and the posterior modes of their expected measurement (Expected Measurement) for the null and undermeasurement model. Our null model assumes* $E[log(y)_{i,j,k}] = log(N_{i,j,k})$ *implying a linear 1:1 relationship between the two. In the undermeasurement model, the expected measurement is* $log(N_{i,j,k}\theta_{i,j,k})$ *for undermeasured replicates and* $log(N_{i,j,k})$ *for replicates that are not undermeasured. We provide 95% HPD intervals for the "inverse prediction" of the expected measurements to convey estimation uncertainty, and these intervals generally have roughly nominal coverage in simulated data sets that meet model assumptions exactly.*

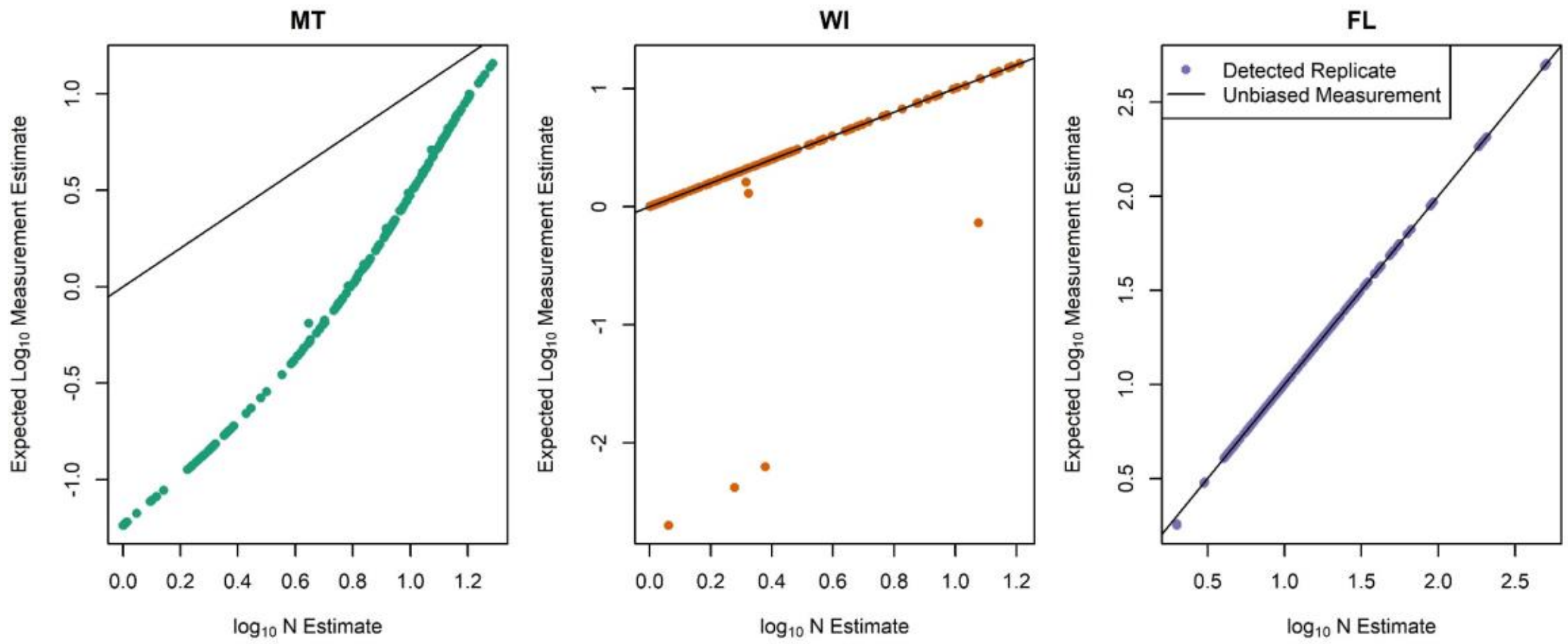

Figure S2: *Plots of the estimated* log_{10} *copy numbers in each replicate* ($N_{i,j,k}$) *vs. the estimated* log_{10} *expected value for measurement* ($N_{i,j,k}^{Measure}$) *from the undermeasurement models at all three sites. eDNA concentrations at the MT site appear systematically underestimated (below 1:1 line), the WI site appears to have 4-6 underestimated replicates, and FL site estimates appear effectively unbiased, with a very minor deviation below 0.5* $log10$ *copies.*

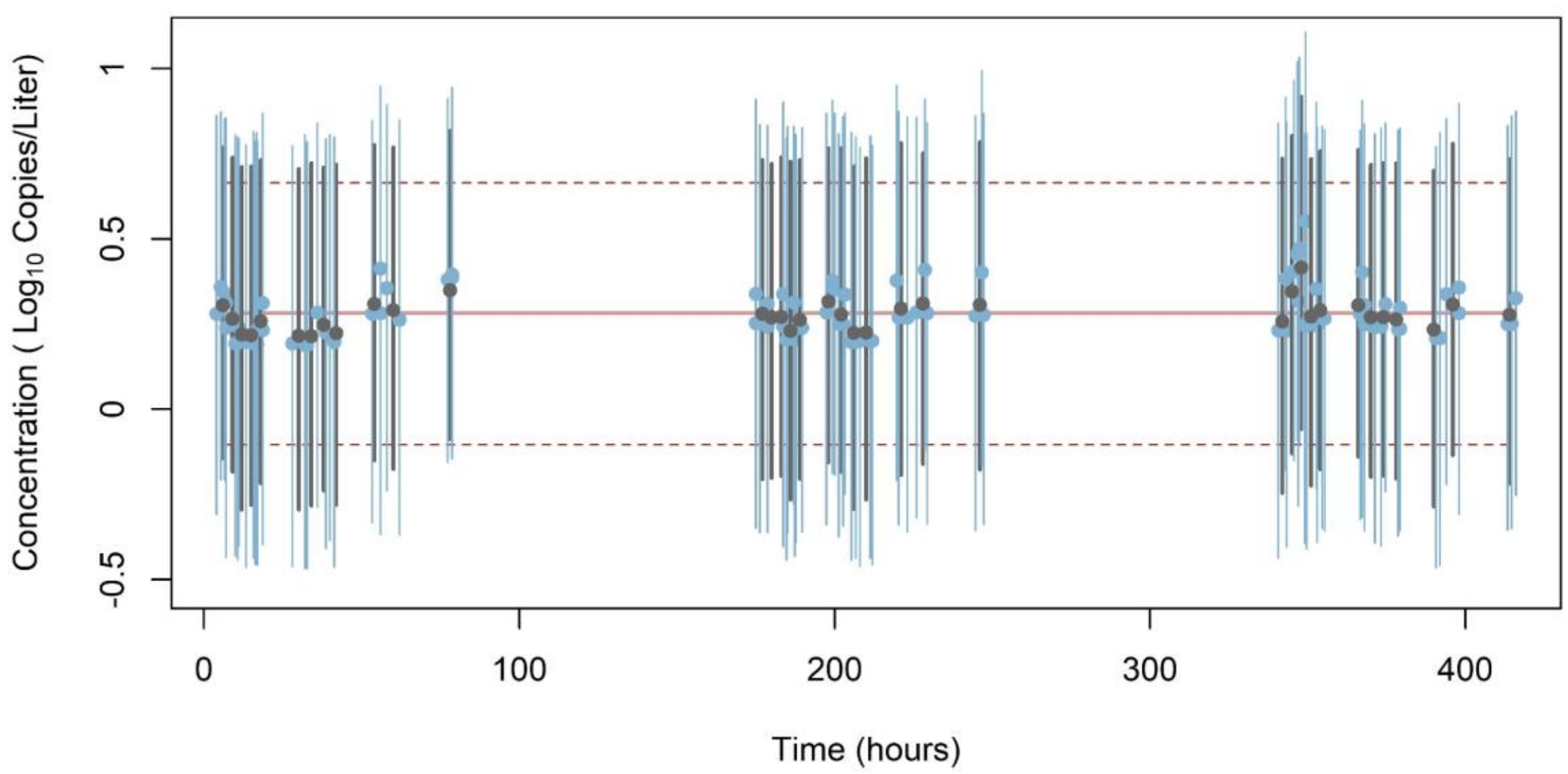

Figure S3. *Plots of the estimated expected site concentration (Mean C), the temporal concentration at sampled times (Temporal C), and the concentration of each sample (Sample C) for the empirical data set collected at the ID site. Point estimates are posterior modes and interval estimates are 95% HPD intervals.*
